# Dysregulated immunity in PID patients with low GARP expression on Tregs due to mutations in *LRRC32*

**DOI:** 10.1038/s41423-021-00701-z

**Published:** 2021-05-31

**Authors:** Peter Lehmkuhl, Magdalena Gentz, Andres Caballero Garcia de Otezya, Bodo Grimbacher, Hendrik Schulze-Koops, Alla Skapenko

**Affiliations:** 1 Division of Rheumatology and Clinical Immunology, Department of Internal Medicine IV, Ludwig-Maximilians-University of Munich, Munich, Germany; 2 Institute for Immunodeficiency, Centre of Chronic Immunodeficiency, Medical Centre, Faculty of Medicine, Albert-Ludwigs-University of Freiburg, Freiburg, Germany

**Keywords:** T cells, immune dysregulation, autoimmunity, Autoimmunity, Severe combined immunodeficiency

## Abstract

Immune dysregulation diseases are characterized by heterogeneous clinical manifestations and may have severe disease courses. The identification of the genetic causes of these diseases therefore has critical clinical implications. We performed whole-exome sequencing of patients with immune dysregulation disorders and identified two patients with previously undescribed mutations in *LRRC32*, which encodes glycoprotein A repetitions predominant (GARP). These patients were characterized by markedly reduced numbers and frequencies of regulatory T cells (Tregs). Tregs with mutated *LRRC32* exhibited strongly diminished cell-surface GARP expression and reduced suppressor function. In a model of conditional Garp deficiency in mice, we confirmed increased susceptibility to inflammatory diseases once GARP expression on Tregs was decreased. Garp deficiency led to an unstable Treg phenotype due to diminished Foxp3 protein acetylation and stability. Our study reinforces the understanding of the immunological mechanisms of immune dysregulation and expands the knowledge on the immunological function of GARP as an important regulator of Treg stability.

## Introduction

Primary immunodeficiency (PID) is a heterogeneous group of disorders characterized by impaired immune development and function due to single-gene mutations. The clinical manifestations of PID are variable and include severe infections, autoimmunity and malignancies.^1^ PID conditions that are associated with autoimmune diseases due to defects in the regulation of self-tolerance are referred to as immune dysregulation diseases.^2^ Currently, several mutations leading to immune dysregulation, such as *FOXP3* mutations causing immune dysregulation, polyendocrinopathy, enteropathy, or X-linked syndrome^3^ and cytotoxic T-lymphocyte-associated protein 4 (*CTLA4*) mutations resulting in CTLA4 haploinsufficiency,^4,5^ have been identified. Due to the heterogeneity of these diseases, it is highly likely that mutations in other genes may also cause immune dysregulation. The identification of these mutations has critical clinical implications.

Control of immunological self-tolerance is performed by regulatory T cells that coexpress CD4 and CD25 (Tregs).^6,7^ The function of Tregs is critically dependent on the Treg-specific transcription factor Foxp3.^8–10^ In addition to Foxp3 expression, the expression of glycoprotein A repetitions predominant (GARP), also known as leucine-rich repeat containing 32 (LRRC32)^11–13^ and encoded by *LRRC32* in humans, characterizes Tregs. GARP is an 80 kDa cell-surface protein that contains 20 leucine-rich repeats.^14^ It associates with inactive latent transforming growth factor β1 (TGFβ1),^11–13^ which consists of a TGFβ1 homodimer bound to latency-associated peptide (LAP). GARP releases active TGFβ1 upon the interaction of LAP with the integrin αVβ8 and thereby regulates the bioavailability of TGFβ1.^15,16^ Active TGFβ1 released by GARP facilitates the development of additional Tregs or T helper 17 (Th17) cells in a paracrine manner and mediates the immunosuppressive capacity of Tregs.^13,17^ In this study, we report two PID patients with previously undescribed *LRRC32* mutations suffering from severe immune dysregulation and exhibiting Treg defects. By using conditional Garp-deficient mice, we confirmed increased susceptibility to inflammatory diseases in the absence of GARP and deciphered the underlying molecular mechanism.

## Methods

### Ethics

All patients and healthy individuals provided written informed consent. The study was approved by the Ethics Committee of the Universities of Munich and Freiburg.

### Whole-exome sequencing

Genomic DNA was purified from human peripheral blood mononuclear cells (PBMCs) using QIAamp kits (Qiagen, Hilden, Germany) according to the manufacturer’s protocol. Whole-exome sequencing (WES) was performed using the custom SureSelect exome sequencing protocol from Agilent (Santa Clara, CA). Exomes were enriched by using SureSelect exome v5 probes. Libraries were sequenced twice (two flow cells) on a HiSeq 2500 v4 with a 2 × 76 bp protocol generating four raw sequence data files (FASTQ) per sample. Data preprocessing was performed according to the GATK best practices and involved the following steps: (1) conversion of FASTQ files into an unmapped BAM file (PICARD tool FastqToSam), (2) addition of tags to the Illumina adapter sequences of the unmapped BAM file (PICARD tool MarkIlluminaAdapters), (3) conversion of the unmapped tagged BAM file into a FASTQ file (PICARD tool SamToFastq), (4) alignment to the reference genome build UCSC hg38 (BWA MEM), (5) identification of duplicated reads (MarkDuplicates PICARD), (6) BAM recalibration, and (7) indel realignment. Variant calling was performed with three different variant callers: GATK Haplotype caller, FreeBayes, and SAMtools. BASH and R scripts were subsequently used to (1) merge the VCF files, (2) identify and unify dinucleotide changes, and (3) format the data sets for importation into an in-house specialized SQL database (GemmaDB) at the Centre of Chronic Immunodeficiency in Freiburg. Variant annotation was performed using Ensembl’s Variant Effect Predictor tool (https://www.ensembl.org/info/docs/tools/vep/index.html), and allele frequency (AF) data were extracted from the gnomAD exome (v2.1.1) and genome (v3) data sets (https://gnomad.broadinstitute.org/downloads). Individual frequencies were obtained by transforming the gnomAD AF data. Variant filtering was performed by selecting variants with (1) an individual frequency below 1% in both our internal cohort and the gnomAD (exomes or genomes) populations, which included control cohorts, such as those in the NHLBI-GO Exome Sequencing Project or the 1000 Genomes project, (2) a “high” or “moderate” predicted impact, (3) an alternative AF1 larger than 0.3 and read depth larger than 20, and (4) a zygosity matching an autosomal recessive or X-linked recessive mode of inheritance, since there was no family history of disease and de novo variants could not be identified without parents (variants in genes associated with an autosomal dominant condition were also assessed, to not exclude genes with incomplete penetrance, and the results were limited to only one transcript per variant (that with the highest score)). Resulting candidate variants were assessed individually considering gene function and disease role.

### Mice

Conditional Lrrc32 knockout mice (C57BL/6.Lrrc32^fl/fl^;Cd4-Cre) were generated by flanking the second exon of Lrrc32 with loxP sites (C57BL/6.Lrrc32^fl/fl^) and subsequently crossing homozygous Lrrc32-floxed mice with C57BL/6NTac-TgN(Cd4-Cre) mice (Taconic, Laven, Denmark) bearing the cre recombinase cassette under control of the mouse Cd4 promoter (genOway, Lyon, France). Control C57BL/6J and B6SJLF1/J mice expressing CD45.1 on T cells were purchased from Janvier (Le Genest-Saint-Isle, France). B6.129S7-Rag1^tm1Mom^/J mice were obtained from The Jackson Laboratory (TJL) (Bar Harbor, ME). Mice were housed under specific pathogen-free conditions. All animal experiments were approved by the Regierung von Oberbayern and performed in compliance with the guidelines of the German Tierschutzgesetz.

### Antibodies and reagents

The following antibodies were used for flow cytometry: fluorescein isothiocyanate (FITC)-labeled anti-mouse CD3 (17A2), phycoerythrin (PE)/cyanine 7 (PE/Cy7)-labeled anti-mouse CD4 (GK1.5), PE-labeled anti-mouse CD25 (PC61.5), allophycocyanin (APC)-labeled anti-mouse GARP (YGIC86), APC-labeled and unconjugated rat anti-mouse Foxp3 (FJK-16s), APC-labeled anti-human Foxp3 (PCH101), PE-labeled anti-human GARP (G14D9), polyclonal rabbit anti-SMAD3, rat IgG2aκ isotype control, PE/Cy7-labeled rat IgG2aκ isotype control, APC-labeled Armenian hamster IgG isotype control, Alexa Fluor 488-labeled goat anti-rat IgG, Alexa Fluor 488-labeled goat anti-rabbit IgG, and Alexa Fluor 594-labeled goat anti-rat IgG (Thermo Fisher Scientific, San Diego, CA); PE-labeled anti-mouse CD19 (1D3), PE-labeled anti-human CD25 (M-A251), FITC-labeled anti-human CD25 (M-A251), PE-labeled anti-human CD56 (MY31), FITC-labeled anti-human CD8 (SK1), FITC-labeled anti-human CD45RA (HI100), PE-labeled anti-human CD45RO (UCHL1), PE-labeled mouse IgG1κ isotype control, and PE-labeled rat IgMκ isotype control (BD Biosciences, San Diego, CA); FITC-labeled anti-human CD3 (UCHT1) and FITC-labeled anti-human CD14 (UCHM-1) (Sigma-Aldrich, St. Louis, MO); PE-labeled anti-mouse CD25 (7D4) (Miltenyi Biotec, Bergisch Gladbach, Germany); APC-labeled anti-human CD4 (RPA-T4), PE/Cy7-labeled anti-human LAP (TW4-2F8), APC-labeled anti-mouse LAP (TW7-16B4), APC-labeled anti-mouse CD45.1 (A20), PE-labeled anti-mouse CD45.2 (104), APC-labeled anti-mouse CD152 (CTLA4) (UC10-4B9), PE/Cy7-labeled anti-mouse CD304 (neuropilin 1 (NRP1)) (3DS304M), APC-labeled anti-mouse interleukin-10 (IL-10) (JES3-16E3), PE/Cy7-labeled anti-acetylated lysine (anti-AcK) (15G10), and PE/Cy7-labeled mouse IgG1κ (MOPC-21) isotype control (BioLegend, San Diego, CA); rabbit anti-AcK (RM101; Abcam, San Francisco, CA); rabbit anti-phospho-SMAD2 (Ser465/467)/SMAD3 (Ser423/425) (D27F4) and rabbit IgG isotype control (Cell Signaling, Danvers, MA); and rat anti-human SMAD3 (378611) and PE-labeled goat anti-rabbit IgG (R&D Systems, Minneapolis, MN). APC-labeled Annexin V (Thermo Fisher Scientific) and Fixable Viability Dye eFluor 780 (Life Technologies Invitrogen, San Diego, CA) were used to detect apoptotic cells. The following reagents were used in cell culture experiments: hamster anti-mouse CD3ε (145-2C11), hamster anti-mouse CD28 (37.51), and mouse anti-human CD28 (CD28.2) (Biosciences); mouse anti-human CD3 (OKT3) (produced in our laboratory); recombinant mouse TGFβ1 and mouse IL-2 (R&D Systems); human IL-2 (Novartis, Basel, Switzerland); carboxyfluorescein succinimidyl ester (CFSE) (Life Technologies Invitrogen); and cycloheximide (CHX) (Sigma-Aldrich). Chicken type II collagen (Chondrex, MD Biosciences, Oakdale, MN), complete Freund’s adjuvant and pertussis toxin (Sigma-Aldrich), myelin oligodendrocyte glycoprotein (MOG_35-55_, amino acids 35-55: MEVGWYRSPFSRVVHLYRNGK) (R&D Systems), and dextran sulfate sodium (DSS) salt (MP Biomedicals, Solon, OH) were used in in vivo experiments.

### T cell isolation

For human T cells, PBMCs were isolated by centrifugation of blood over a Ficoll (Biotest, Dreieich, Germany) layer. Overall, 10 ml of Ficoll was loaded under 20 ml blood diluted with 20 ml phosphate-buffered saline (PBS) and centrifuged at 400 × *g* for 20 min at room temperature. PBMCs were harvested and washed once with PBS. Total CD4^+^ T cells, naive CD4^+^ T cells, CD25^-^ CD4^+^ T cells and CD25^+^ CD4^+^ T cells were purified from human PBMCs by magnetic cell separation (MACS) using the Human CD4^+^ T Cell or CD4^+^ CD25^+^ Regulatory T Cell Isolation Kit (Miltenyi Biotec).

For murine T cells, spleens were homogenized into a single-cell suspension using gentleMACS™ C Tubes and a gentleMACS™ Dissociator (Miltenyi Biotec). Cell populations were purified from splenocytes either by MACS or by fluorescence-activated cell sorting (FACS) on a MoFlo (Beckman Coulter, Brea, CA). Total CD4^+^ T cells were purified using the Mouse CD4^+^ T Cell Isolation Kit (Miltenyi Biotec). CD25^+^ CD4^+^ and CD25^−^ CD4^+^ T cells were purified using the Mouse CD4^+^ CD25^+^ Regulatory T Cell Isolation Kit (Miltenyi Biotec). The purity of the cell populations was evaluated by flow cytometry.

### Flow cytometry

For extracellular staining, cells were incubated with antibodies for 15 min at 4 °C, washed with 2% fetal calf serum (FCS) in PBS (both from Life Technologies Invitrogen) and analyzed on a Cytomics FC 500 (Beckman Coulter).

For intracellular staining of Foxp3 and for staining of CD25, NRP1, and CTLA4, cells were fixed and permeabilized using the Foxp3 Fix/Perm buffer set (BioLegend). The True-Nuclear™ Transcription factor buffer set (BioLegend) was used for fluorescence resonance energy transfer (FRET)^18^ staining. Cytofix/Cytoperm™ Fixation and Permeabilization Solution with Phosflow™ Perm Buffer III (both from BD Bioscience) was used for SMAD staining. Afterwards, cells were incubated with antibodies for 30 min at 4 °C, washed with 2% FCS/PBS and analyzed on a Cytomics FC 500 (Beckman Coulter). For measurement of Foxp3 acetylation, a FRET antibody pair for Foxp3 detected with acceptor Alexa Fluor 594-labeled goat anti-rat IgG and AcK detected with donor Alexa Fluor 488-labeled goat anti-rabbit IgG (all from Thermo Fisher Scientific) was used. The FRET signal was recorded at 600–620 nm after excitation with a 488 nm laser.

For intracellular IL-10 staining, 1 × 10^6^ cells were stimulated in RPMI 1640 medium supplemented with 50 U/ml penicillin G, 50 µg/ml streptomycin, 2 mM L-glutamine, and 10% FCS (all from Life Technologies Invitrogen) with 1 ng/ml phorbol myristate acetate, 2 µM monensin (both from Sigma-Aldrich), and 0.5 µM ionomycin (Merck) for 5 h. The cells were fixed with 4% paraformaldehyde (PFA) (Roth, Karlsruhe, Germany) for 10 min at 37 °C and permeabilized using 2% FCS/PBS with 0.1% saponin (Sigma-Aldrich). The cells were incubated with antibodies for 30 min at 4 °C, washed with 2% FCS/PBS, and analyzed on a Cytomics FC 500 (Beckman Coulter).

### Collagen-induced arthritis

Collagen-induced arthritis (CIA) was induced in 12-week-old mice by intradermal injections of 200 µg chicken collagen (Chondrex) in complete Freund’s adjuvant (Sigma-Aldrich) followed by a booster injection of 200 µg collagen in incomplete Freund’s adjuvant (Sigma-Aldrich) on day 21. The mice were scored every 2–3 days to assess the redness and swelling of each limb (0–3, with a maximum score of 12 per mouse): 0, normal; 1, slight swelling and/or erythema; 2, pronounced edematous swelling; and 3, joint rigidity. Hind paw thickness was assessed on the day of the first injection (day 0) and at the experimental endpoint (day 38).

### Experimental autoimmune encephalomyelitis

Experimental autoimmune encephalomyelitis (EAE) was induced by subcutaneous immunization of 12-week-old mice with 200 µg MOG_35-55_ (R&D Systems) in complete Freund’s adjuvant and 400 ng pertussis toxin (both from Sigma-Aldrich). On day 2, an additional 400 ng pertussis toxin was administered by subcutaneous injection. The mice were scored daily for signs of paralysis beginning on day 7 after immunization until the experimental endpoint (day 19). The EAE clinical score was assessed as follows: 0, clinically normal; 1, limp tail; 2, weak hind limbs; 3, partially paralyzed hind limbs; 4, complete hind limb paralysis; and 5, death.

### DSS-induced colitis

Colitis was induced in 8-week-old mice by administration of 2% DSS (MP Biomedicals) in the drinking water. Body weight was monitored daily for 9 days. On day 9, the mice were sacrificed. Colon sections were stained and evaluated to determine colitis clinical scores using TJL scoring system (0–12): severity (0–3), ulceration (0–3), hyperplasia (0–3), and area involved (0–3).^19^ Lymphocyte patch numbers were counted in ten 100 µm sequential sections.

### Histological staining

Tissues were fixed in 4% PFA (Roth) for 8-12 h at room temperature and embedded in paraffin wax using standard histological procedures. Paws were decalcified in 10% EDTA (Sigma-Aldrich), pH 7.2, for 2 weeks at 4 °C and washed overnight in 70% ethanol prior to embedding. One-micrometer paraffin sections were stained with hematoxylin (Sigma-Aldrich) and eosin (Merck, Darmstadt, Germany) (H&E staining) for morphological assessment. Immunohistochemical staining for CD3 + cell infiltration was performed as previously described.^20^ Tartrate-resistant acid phosphatase (TRAP) staining was performed using the Acid Phosphatase, Leukocyte (TRAP) Kit (Sigma-Aldrich) following the manufacturer’s instructions.

### Adoptive transfer

CD45.1^+^ CD25^−^ CD4^+^ T cells (2 × 10^6^) purified from B6SJLF1/J mice were injected intravenously into 12-week-old B6.129S7-Rag1^tm1Mom^/J mice either alone or together with 5 × 10^5^ CD45.2^+^ Tregs purified from control (C57BL/6J) or Garp-deficient (C57BL/6.Lrrc32^fl/fl^;Cd4-Cre) animals. Splenocytes were analyzed by flow cytometry at 7 days post transplantation.

### Plasmid constructs

pcDNA3.1 expression vectors encoding the following human GARPcds constructs were generated by the GeneArt service (Thermo Fisher Scientific): (1) GARP_wt (wild-type (wt) sequence, wt protein), (2) GARP_c.741G>A (p.Trp247Ter), (3) GARP_c.934C>T (Arg312Cys), and (4) GARP_c.1262G>A (p.Arg421Gln).

### Transfection

Human embryonic kidney cells (HEK293) were obtained from American Type Culture Collection (No. CRL-1573). HEK293 cells were transfected with plasmid DNA using Lipofectamine^TM^ LTX (Life Technologies Invitrogen) according to the manufacturer’s instructions. Briefly, 0.25 × 10^6^ cells were seeded in 12-well plates and transfected on the following day with 0.4 μg pcDNA3.1-GARPcds variants GARP_wt, GARP_c.741G>A, GARP_c.934C>T, and GARP_c.1262G>A (Thermo Fisher Scientific) or the empty pcDNA3.1 vector as a control. Plasmid DNA was dissolved in 200 µl Opti-MEM and incubated with 3.5 µl Lipofectamine LTX (both from Life Technologies Invitrogen) for 30 min at room temperature. The DNA/Lipofectamine complexes were added to HEK293 cells cultured in 1 ml Dulbecco’s modified Eagle’s medium supplemented with 50 U/ml penicillin G, 50 µg/ml streptomycin, 2 mM l-glutamine, and 10% FCS (all from Life Technologies Invitrogen). The expression of GARP was analyzed 48 h after transfection by flow cytometry.

### T cell culture

Human T cells were stimulated with plate-bound anti-CD3 (1 µg/ml) and soluble anti-CD28 (1 µg/ml) (BD Bioscience) antibodies in RPMI 1640 medium supplemented with 50 U/ml penicillin G, 50 µg/ml streptomycin, 2 mM l-glutamine (all from Life Technologies Invitrogen), 10% normal human serum (NHS), and 10 U/ml IL-2 (Novartis) for the indicated amount of time.

Murine T cells were cultured without stimulation or stimulated with plate-bound anti-CD3 (2 µg/ml) and anti-CD28 (10 µg/ml) (BD Bioscience) antibodies in RPMI 1640 medium supplemented with 50 U/ml penicillin G, 50 µg/ml streptomycin, 2 mM l-glutamine, 10% FCS (all from Life Technologies Invitrogen), and 10 ng/ml IL-2 (R&D Systems) for the indicated amount of time. Mouse TGFβ1 (2 ng/ml, R&D Systems) was added when indicated. For serum starvation, cells were incubated with serum-free RPMI 1640 medium supplemented with 50 U/ml penicillin G, 50 µg/ml streptomycin, 2 mM l-glutamine (Life Technologies Invitrogen) and 10 ng/ml IL-2 (R&D Systems) for 20 h.

### Analysis of protein stability

CHX (Sigma-Aldrich) was dissolved in ethanol (40 mg/ml) and diluted in PBS to generate working solutions. CHX (6 µg/ml, Sigma-Aldrich) was used in cell culture experiments to determine the half-life of Foxp3. Murine T cells were stimulated as described above for 3, 6, 9, and 20 h. Human T cells were stimulated as described above for 6, 20, and 48 h. Foxp3+ cell frequencies were assessed by intracellular flow cytometry. The half-life was calculated by linear regression of normalized Foxp3+ cell frequencies.

### In vitro suppression assay

Freshly isolated human naive or total CD25^−^ CD4^+^ T cells were resuspended in PBS at a concentration of 100 × 10^6^/ml and labeled with 10 µM CFSE (Life Technologies Invitrogen) for 8 min at room temperature. After labeling, the cells were resuspended in 10% NHS/RPMI 1640 medium (Life Technologies Invitrogen). A total of 50 × 10^3^ CFSE-labeled CD25^−^ CD4^+^ T cells were stimulated in a 96-well round-bottom plate with 1 µg/ml soluble anti-CD3 antibody in the presence of 100 × 10^3^ CD4^−^ CD8^−^ PBMCs and increasing amounts of Tregs for 4 days.

### Real-time PCR analysis

Total RNA was isolated using the RNeasy Plus Mini Kit (Qiagen) according to the manufacturer’s instructions. Total RNA (0.1–1 μg) was reverse transcribed into cDNA using the Affinity Script QPCR cDNA Synthesis Kit (Agilent Technologies).

mRNA expression was detected using a TaqMan gene expression assay (Hs00973758_m1—*LRRC32*, Hs00206843_m1—*HDAC9*, Hs99999903_m1—*ACTB*, Mm02391771_g1—Hdac1, Mm005155108_m1—Hdac2, Mm00515916_m1—Hdac3, Mm01299557_m1—Hdac4, Mm01341125_m1—Hdac6, Mm01293999_m1—Hdac9, and Mm00607939_s1—Actb; all from Thermo Fisher Scientific). Relative expression was calculated as the difference in cross-threshold values (ΔCt) of the gene of interest and the beta actin gene according to the formula 2^−ΔCt^. All PCRs were performed on a 7500 fast real-time PCR system (Thermo Fisher Scientific).

*LRRC32* allelic expression was examined by allele-specific PCR analysis using assays-by-design for single-nucleotide polymorphism genotyping (Thermo Fisher Scientific). The assay consisted of two unlabeled PCR primers (forward and reverse), a VIC/minor groove binder (MGB)-labeled probe detecting the major allele sequence and a FAM/MGB-labeled probe for the minor allele sequence. The dye emission was measured after 40 cycles.

### Gene array

Transcriptome analysis was performed on a whole mouse genome oligo microarray (Mouse GE 4x44K v2) (Agilent Technologies) using mRNA purified from FACS-sorted Garp-deficient and control Tregs from five mice per group. The intensity data were subjected to quantile normalization. Fold changes in normalized data were calculated to identify genes with greater than twofold differential expression; log2 intensities of selected genes were median-centered and visualized by Mayday (version 2.30) using centering for display.

### Molecular imaging

Molecular images were generated with PyMOL (The PyMOL Molecular Graphics System, Schrödinger, LLC) using the PDB file 6GFF.

### Statistical analysis

Statistical analysis was performed by using Student’s *t*-test. The Kolmogorov–Smirnov test was used to test for a normal distribution. *p* values less than 0.05 were considered statistically significant and are indicated as *p < 0.05, **p < 0.01, and ***p < 0.001 (n.s.—not significant).

## Results

### Identification of PID patients with *LRRC32* mutations

**Fig. 1:**
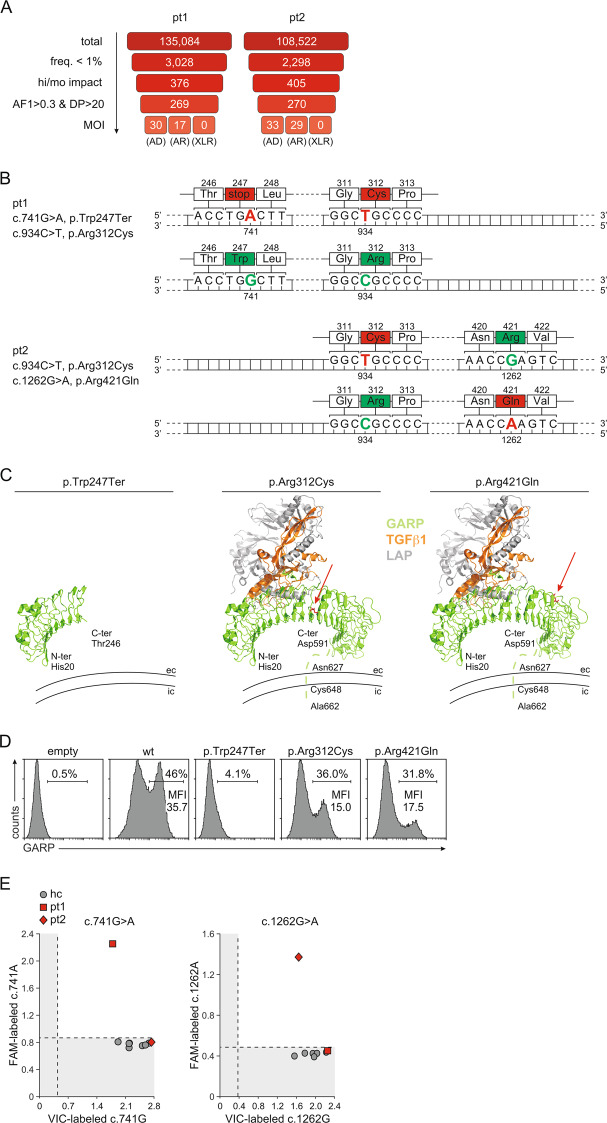
Identification of patients with mutant *LRRC32* variants. **A** The filtering strategy for exome sequencing data with indication of variant numbers at each step is demonstrated. freq., individual frequency in our internal cohort and in gnomAD exomes and genomes; hi/mo, predicted high or moderate impact of a mutation on gene function/structure based on the type of variant (e.g., a “stop_gained” variant is predicted as “high”, whereas a missense variant is predicted as “moderate”); AF1 frequency of reads with the alternative allele, DP read depth (number of reads covering a variant), MOI mode of inheritance, AD autosomal dominant, AR autosomal recessive, XLR X-linked recessive. **B** Schematic presentation of *LRRC32* alleles in patient 1 and patient 2. Affected nucleotides and amino acids and their positions are indicated and denoted by green and red for the wt and mutant variants, respectively. **C** The predicted protein structure of each mutant GARP proteins in complex with TGFβ1 and LAP is depicted. GARP is indicated in green, TGFβ1 is indicated in orange, and LAP is indicated in gray. Mutated amino acids are indicated in red and noted with arrows. The dashed line indicates part of GARP with a nonresolved crystal structure. N-ter N-terminus, C-ter C-terminus, er extracellular, ic intracellular. **D** Expression of mutated GARP proteins on the surface of transfected HEK293 cells after staining for GARP. Representative staining out of three independent experiments with similar results is shown. **E** Analysis of allelic *LRRC32* expression in patients. Total mRNA was purified from Tregs and analyzed by allele-specific PCRs for c.741G>A and c.1262G>A in triplicate. Mean values are indicated. Results from patients are indicated in red. Individual results from seven healthy controls are indicated in gray. The probes for the wt and mutant alleles were labeled with VIC and FAM, respectively, and duplexed in one reaction. Shaded areas indicate background fluorescence

By routine screening of our patients at the immunodeficiency clinic using WES, we identified two patients bearing previously undescribed mutations in *LRRC32* (Fig. 1A, Table 1, Supplementary Tables 1 and 2). Patient 1 had a total of 135,084 variants with respect to the reference genome (build hg38). Subsequent filtering steps resulted in 30 heterozygous candidate variants in genes previously associated with autosomal dominant conditions and 17 compound heterozygous or homozygous variants in genes not previously associated with disease or associated with autosomal recessive conditions. Of these, two heterozygous variants in the gene *LRRC32* were the most promising candidates: a novel nonsense mutation (p.Trp247Ter) and a rare missense variant (p.Arg312Cys) (Fig. 1B). Patient 2 had a total of 108,522 variants with respect to the reference genome. Subsequent filtering steps resulted in 33 heterozygous candidate variants in genes previously associated with autosomal dominant conditions and 29 compound heterozygous or homozygous variants in genes not previously associated with disease or associated with autosomal recessive conditions. Of these, two rare heterozygous missense variants in the gene *LRRC32* were the most promising candidates: p.Arg421Gln and p.Arg312Cys (Fig. 1B). Sanger sequencing of these patients proved that these variants were in the same allele in patient 1 and in different alleles in patient 2.

**Table 1:** *LRRC32* variants in PID patients

Pat.	Chromosomic change (hg38)	CDS change	Amino acid change	Variant type	dnSNP	AF gnomAD exomes	AC/AN gnomAD exomes
1	11-76660852-C-T	c.741G>A	p.Trp247Ter	stop_gained	–	–	–
	11-76660659-G-A	c.934C>T	p.Arg312Cys	missense_variant	rs143082901	0.00031237	78/249706
2	11-76660659-G-A	c.934C>T	p.Arg312Cys	missense_variant	rs143082901	0.00031237	78/249706
	11-76660331-C-T	c.1262G>A	p.Arg421Gln	missense_variant	rs200320285	0.00005036	12/238306

Nomenclature according to both *Ensembl*’s canonical transcript ENST00000407242.6 and to *RefSeq*’s canonical transcript NM_001128922.2

*AF* allele frequency, *AC* allele count, *AN* allele number

Based on the recently resolved crystal structure of the GARP/latent TGFβ1 complex,^21^ we predicted mutated protein structures (Fig. 1C). Prediction of the p.Trp247Ter protein structure resulted in a truncated protein missing the transmembrane region. The predicted p.Arg312Cys structure produced a full-length protein with the exchange of a hydrophilic, positively charged Arg at position 312 to uncharged Cys. The predicted p.Arg421Gln structure was also a full-length protein with the exchange of positively charged Arg to uncharged Gln. Both the Arg312Cys and Arg421Gln exchanges were found to be located at the convex side of the horseshoe and to not interfere with the binding faces for the latent TGFβ1 complex of GARP. However, the prediction of protein stability based on the free energy change (*∆∆G*)^22^ for both mutants resulted in destabilizing *∆∆G* values much below −0.5 Kcal/mol, −2.93 Kcal/mol for p.Arg312Cys and −2.79 Kcal/mol for p.Arg421Gln, suggesting lower protein stability and therefore lower protein expression. Indeed, analysis of mutated GARP protein expression using corresponding plasmids revealed no expression for the p.Trp247Ter-encoding construct and strongly diminished expression of the p.Arg312Cys and p.Arg421Gln variants (Fig. 1D).

We next analyzed the allelic expression of *LRRC32* in Tregs from the patients (Fig. 1E). Allele-specific PCR assays for c.741G>A and c.1262G>A were generated and used for the analysis of *LRRC32* mRNA expression. Unfortunately, because of the nucleotide sequence, no assay could be generated for c.934C>T. However, because of duplexing of probes for mutant and wt alleles, we were able to completely analyze allelic *LRRC32* expression: as both FAM and VIC emissions were detected in the c.741G>A assay performed with Tregs from patient 1 and in the c.1262G>A assay performed with Tregs from patient 2, we concluded that *LRRC32* transcription from both alleles occurred in both patients. Tregs from patient 1 therefore expressed wt GARP on the surface, while Tregs from patient 2 expressed the p.Arg312Cys and p.Arg421Gln GARP variants on the surface.

### Clinical and immunologic characteristics of the patients

At the time of investigation, patient 1 was a 65-year-old male suffering from diminished IgG levels and subsequent recurrent sepsis and life-threatening intestinal obstructions. He was diagnosed with common variable immunodeficiency (CVID) at age 54. Lymphocyte infiltration was observed in the gut mucosa but was not as profound as that usually seen with enterocolitis associated with CVID. At the time of blood collection, the patient was receiving intravenous immunoglobulin substitution and prophylactic antibiotics. Patient 2 was a 49-year-old female suffering from recurrent upper respiratory tract infections and diarrhea due to chronic autoimmune granulocytopenia with autoantibodies against granulocytes. Although prescribed, at the time of blood collection, she was not taking prophylactic antibiotics.

**Fig. 2:**
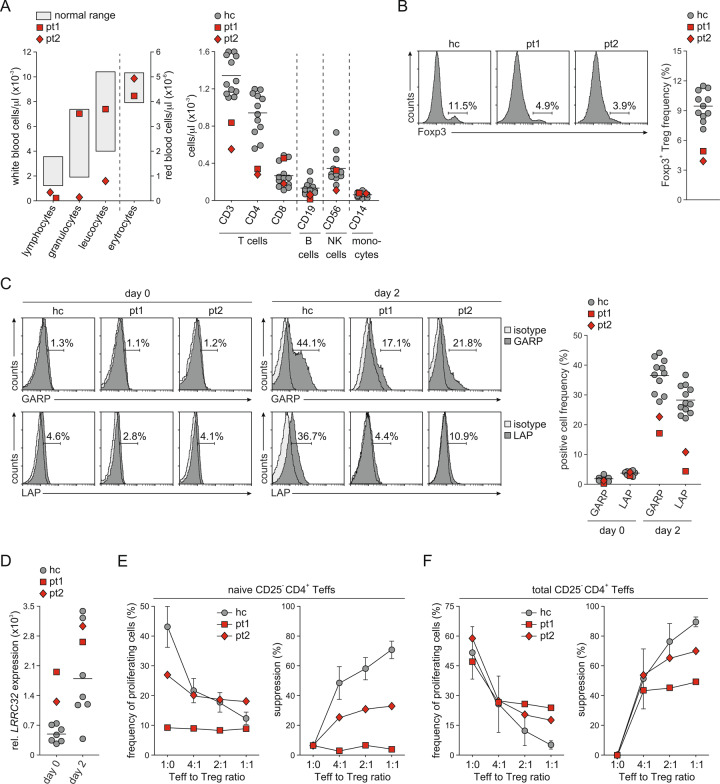
Characterization of Tregs from patients with *LRRC32* mutations. **A** Blood cell analysis is depicted. Left panel: White and red blood cell counts in the peripheral blood of patients analyzed by a central laboratory. Normal ranges defined by the central laboratory for clinical tests are included. Right panel: Cell counts of T cells, B cells, natural killer (NK) cells, and monocytes in the peripheral blood were analyzed by flow cytometry. Results from patients are indicated in red. Individual results and the mean value for 12 healthy controls are indicated in gray. **B** The frequency of Tregs was determined by intracellular flow cytometry for Foxp3 in CD4^+^ T cells. Representative staining of a healthy control (hc)  and staining of both patients are shown. The graph demonstrates the results from the patients in red and the individual results and mean value from 11 healthy controls in gray. **C** The expression of GARP and LAP on the surface of Tregs was assessed by flow cytometry. Tregs were stained directly after purification (day 0) and after stimulation with anti-CD3 and anti-CD28 antibodies for 2 days. Representative staining histograms of a healthy control and both patients are shown. The graph demonstrates the results from the patients in red and the individual results and mean value from 11 healthy controls in gray. **D** *LRRC32* mRNA expression was assessed in freshly purified Tregs and Tregs stimulated for 2 days by real-time PCR in duplicate. Relative *LRRC32* mRNA expression in relation to the mRNA expression of *ACTB* is shown. The graph demonstrates the results from the patients in red and the individual results and mean value from seven healthy controls in gray. The suppressive effect of Tregs on the proliferation of CFSE-labeled naive (**E**) and total (**F**) CD25^−^ CD4^+^ Teffs in response to anti-CD3 was assessed by flow cytometry at different Teff-to-Treg ratios. The frequency of proliferating Teffs and the calculated suppressive capacity of Tregs are depicted. Gray circles indicate data from healthy controls demonstrated as the mean ± SD of five or eight individuals, respectively

Both patients were lymphopenic with particularly reductions in CD4^+^ T cells (Fig. 2A). Moreover, Treg frequencies were strongly diminished in both patients (Fig. 2B). Surface GARP protein expression is upregulated in response to T cell receptor (TCR) stimulation.^23^ We therefore measured GARP expression on Tregs activated for 2 days (Fig. 2C). As a control, we measured the surface LAP expression. Tregs from both patients demonstrated markedly low GARP levels on the surface. Correspondingly, LAP expression was also diminished. Interestingly, however, *LRRC32* mRNA expression was increased in freshly isolated cells (day 0) or at the upper limit of the normal range on day 2 (Fig. 2D). Next, we analyzed the suppressor function of Tregs from these patients (Fig. 2E, F). We used either naive or total CD25^−^ CD4^+^ T cells as the effector T cell (Teff) population. Independent of the effector population used, proportionally more Teffs entered proliferation in the presence of Tregs from patients than in the presence of those from healthy donors, indicating that Tregs with lower GARP expression exhibited a reduced suppressor function. We therefore concluded that mutations in *LRRC32* might lead to diminished GARP levels on the surface of Tregs and consequently to a reduction in Treg numbers and moreover to Treg dysfunction.

### Garp deficiency in mice leads to an inflammatory phenotype

**Fig. 3:**
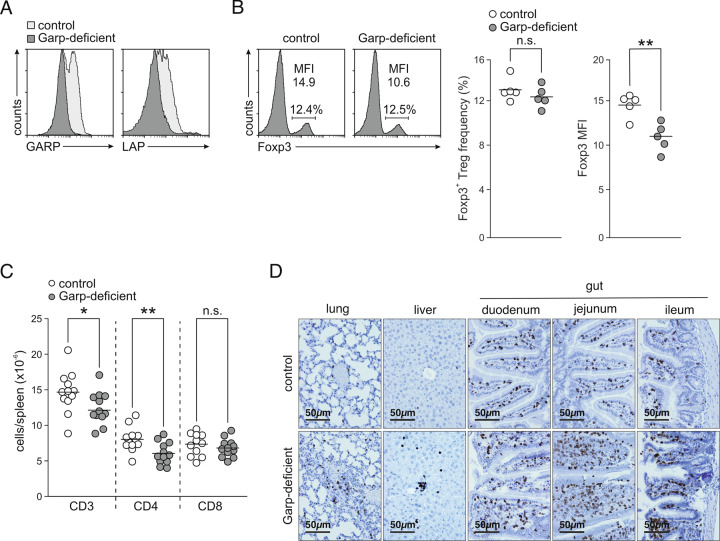
Phenotype of Garp-deficient mice. **A** GARP and LAP levels on the surface of Tregs were assessed by flow cytometry. Representative staining histograms after stimulation with anti-CD3 and anti-CD28 antibodies for 2 days are shown. **B** The Foxp3 level was assessed by intracellular flow cytometry. Representative staining histograms and data for one representative experiment out of four independent experiments with five mice per group are shown. Horizontal lines indicate mean values. MFI mean fluorescence intensity. **C** Counts of T cell populations in spleens from 12 control and 12 Garp-deficient 12-week-old mice. Horizontal lines indicate mean values. **D** Lungs, livers, and intestines from 10- to 12-week-old control and Garp-deficient mice were immunohistochemically stained for CD3. Representative sections are shown

Because of the markedly diminished expression of GARP on the surface of patient Tregs, we analyzed Garp knockout mice (C57BL/6.Lrrc32^fl/fl^;Cd4-Cre) to gain deeper insights into molecular mechanisms beyond Treg dysfunction. Tregs from these animals did not express GARP on the surface (Fig. 3A). Interestingly, in contrast to the patients, the frequencies of Foxp3^+^ Tregs were not diminished in these mice (Fig. 3B). However, knockout mouse Tregs expressed reduced levels of Foxp3. Consistent with the patient findings, Garp-deficient mice demonstrated reduced T cell counts and lymphocyte infiltration in various tissues (Fig. 3C, D).

**Fig. 4:**
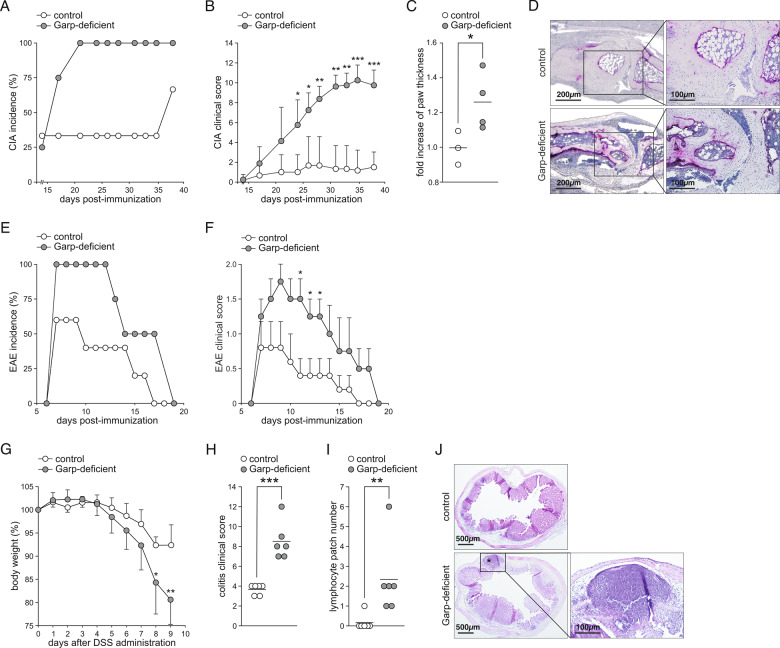
Immune diseases in Garp-deficient mice. **A**–**D** CIA. Incidence of arthritis (**A**), arthritis clinical score reported as the mean ± SEM (**B**), increase in paw thickness from the first day to the last day postimmunization (**C**), and histologic TRAP staining of paws collected on day 38 (**D**). The results of one representative out of three independent experiments with three to five mice per group are shown. **E**, **F** EAE. Incidence of paralysis (**E**) and the EAE clinical score shown as the mean ± SEM (**F**). The results of one representative experiment out of three independent experiments with three to five mice per group are shown. **G**–**J** DSS-induced colitis. Body weight over a period of 9 days shown as the mean ± SEM (**G**), clinical score assessed on the last day of analysis (**H**), number of colonic lymphocyte patches in ten 100 µm sequential sections (**I**), and representative H&E staining of the colon with a lymphocyte patch (**J**). The results of one representative experiment out of five independent experiments with five to six mice per group are shown. Horizontal lines indicate mean values

We next investigated the susceptibility of these animals to autoimmune disorders. Garp-deficient mice, in contrast to control wt animals, were highly susceptible to CIA (Fig. 4A). They developed severe aggravated arthritis (Fig. 4B) with markedly increased paw thickness (Fig. 4C). The severe inflammation in Garp-deficient mice was confirmed by histological analysis, displaying a high degree of lymphocyte infiltration into the joint cleft, increased osteoclast differentiation, and severe cartilage damage (Fig. 4D). Similar to the results for CIA mice, Garp-deficient mice were 100% susceptible to EAE and developed severe paralyzing disease (Fig. 4E, F). Furthermore, Garp-deficient mice developed rapid and devastating colitis in response to DSS administration, characterized by a high clinical score and lymphocyte infiltration into the mucosa (Fig. 4G–J). Thus, in mice, low GARP expression on the surface of Tregs leads to a phenotype characterized by immune dysregulation.

### GARP controls Treg phenotype stability

**Fig. 5:**
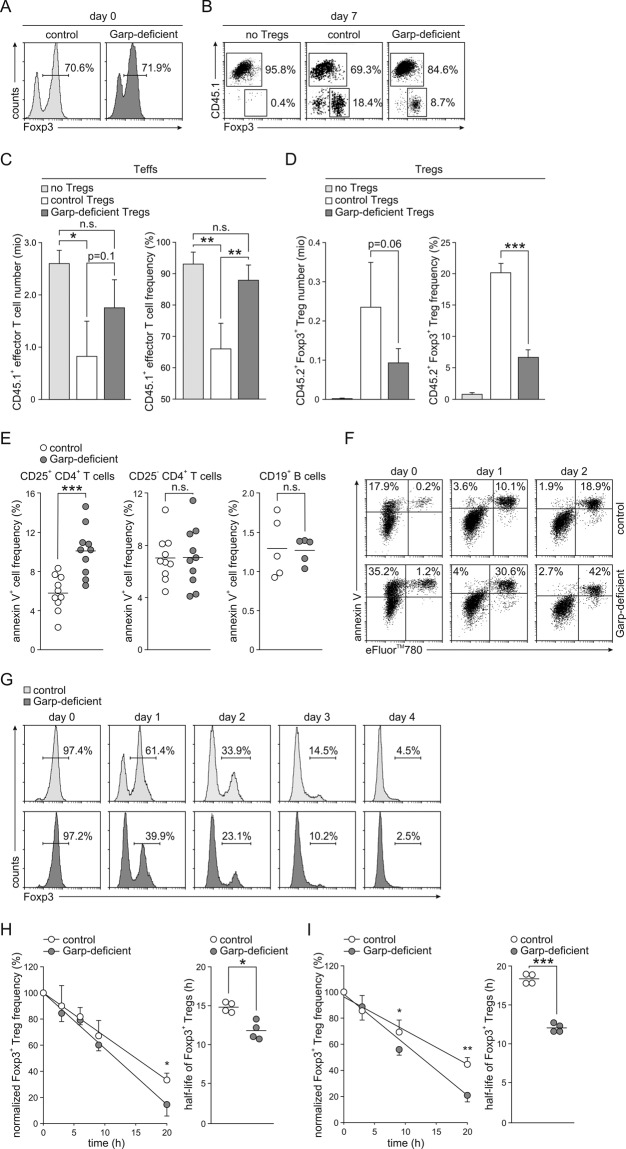
Phenotype of Garp-deficient Tregs. **A**–**D** Adoptive transfer of CD45.1^+^ CD25^−^ Teffs into Rag1-deficient mice alone or together with control or Garp-deficient CD45.2^+^ Tregs. The data from one representative experiment out of four independent experiments with four to five mice per group are shown. **A** Representative Foxp3 staining histograms of MACS-purified Tregs before transfection. **B** Representative staining of recovered CD4^+^ T cells for CD45.1 and Foxp3. **C** Numbers and frequencies of CD45.1^+^ CD4^+^ T cells in splenocytes harvested 7 days after adoptive transfer. Data are shown as the mean ± SD. **D** Numbers and frequencies of CD45.2^+^ Foxp3^+^ CD4^+^ T cells in splenocytes harvested 7 days after adoptive transfer. Data are shown as the mean ± SD. **E** Frequencies of annexin V^+^ cells in CD25^+^ CD4^+^ T cells, CD25^−^ CD4^+^ T cells, and CD19^+^ B cells in freshly isolated splenocytes from five to ten mice. Horizontal lines indicate mean values. Apoptosis of FACS-sorted Tregs pooled from five to ten animals and stimulated with anti-CD3 and anti-CD28 antibodies for 2–4 days determined by staining with annexin V and a cell viability dye (**F**) or an anti-Foxp3 antibody (**G**) and analysis by flow cytometry. One representative experiment out of two independent experiments is shown. **H**, **I** Analysis of Treg stability. Frequency of Foxp3^+^ Tregs within CD4^+^ T cells cultured without stimulation (**H**) or stimulated with anti-CD3 and anti-CD28 antibodies for 20 h (**I**) in the presence of CHX assessed by intracellular flow cytometry. The results of four independent experiments are summarized and shown as the mean ± SD for Treg frequency and as the results of individual experiments with the calculated mean value indicated by the line for the Treg half-life. Linear regression calculated on normalized frequencies of Foxp3^+^ Tregs is shown on the left, and the half-life of Foxp3^+^ Tregs calculated based on linear regression is shown on the right

We then analyzed the regulatory capacity of Garp-deficient Tregs. Tregs were purified from either Garp-deficient or control mice (Fig. 5A) and transferred together with CD45.1^+^ CD25^−^ CD4^+^ Teffs into Rag1-deficient mice. One group of animals received Teffs only. The expansion of CD45.1^+^ CD25^−^ CD4^+^ Teffs in the spleen was analyzed after 7 days (Fig. 5B). As shown in Fig. 5C, strong Teff expansion was detected in animals without Treg cotransfer. The addition of Tregs from control animals significantly inhibited the proliferation of Teffs, whereas the addition of Garp-deficient Tregs had little effect. Remarkably, the insufficient control of Teff expansion by Garp-deficient Tregs correlated with the markedly lower recovery of Garp-deficient Tregs (Fig. 5D). Both the number and frequency of recovered Garp-deficient Tregs were markedly diminished compared to those of control Tregs.

Correspondingly, in in vitro experiments, we found higher frequencies of early apoptotic cells in CD25^+^ CD4^+^ T cells but not in CD25^−^ CD4^+^ T cells or CD19^+^ B cells from Garp-deficient mice (Fig. 5E). Moreover, significantly higher numbers of Garp-deficient Tregs than control Tregs entered late apoptosis in culture (Fig. 5F). Moreover, Garp-deficient Tregs lost Foxp3 expression much faster than control Tregs (Fig. 5G). When we determined the half-life of Foxp3 by using CHX, we found that the half-life of Foxp3 was 11.9 ± 1.0 h in nonstimulated Garp-deficient Tregs and 14.9 ± 0.56 h in nonstimulated control Tregs (*p* = 0.02) (Fig. 4H). Stimulation of Tregs with anti-CD3 and anti-CD28 antibodies did not stabilize Foxp3 in Garp-deficient Tregs but rather accentuated the difference between Garp-deficient and control Tregs. The half-life of Foxp3 in activated Garp-deficient Tregs was 12.1 ± 0.47 h compared to 18.4 ± 0.56 h in activated control Tregs (*p* < 0.005) (Fig. 5I). Thus, as Foxp3 is the key transcription factor of Tregs, these data indicate a critical function for GARP in Foxp3 stability and therefore in Treg stability.

### GARP regulates the expression of Hdac9 and the stability of Foxp3 by acetylation

**Fig. 6:**
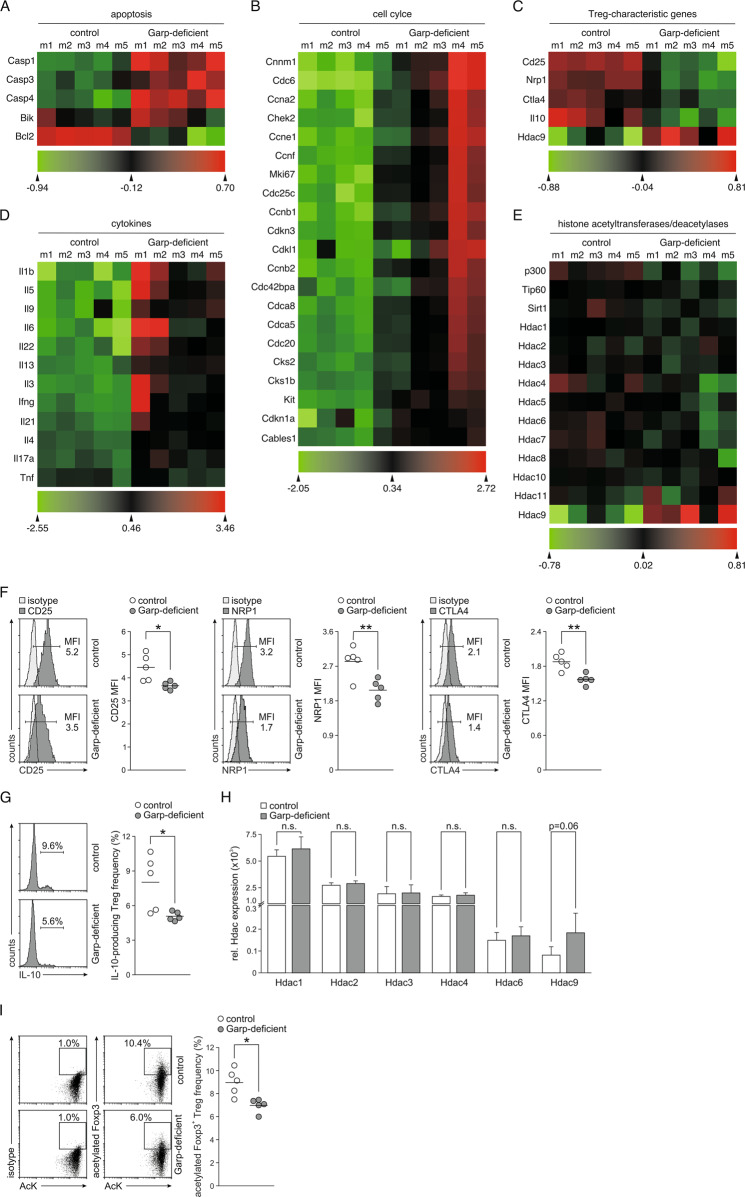
Analysis of Hdac9 expression and Foxp3 acetylation. Heat maps of statistically significant differentially expressed genes involved in **A** apoptosis or **B** cell cycle regulation, **C** characteristic of the Treg phenotype, or **D** encoding cytokines in Garp-deficient and control Tregs. **E** Heat maps of genes encoding histone acetyltransferases and deacetylases. **F**, **G** Expression of CD25, NRP1, CTLA4, and IL-10. The expression of CD25, NRP1, and CTLA4 was measured in freshly isolated Tregs by intracellular flow cytometry after gating for Foxp3 (**F**). The expression of IL-10 was measured in freshly isolated Tregs by intracellular flow cytometry after stimulation with PMA and ionomycin (**G**). Representative staining histograms and a summary of one representative experiment out of two independent experiments with five mice per group are depicted. Horizontal lines indicate mean values. MFI mean fluorescence intensity. **H** mRNA levels of the indicated histone deacetylases in relation to the Actb mRNA level were determined by real-time PCR analysis. The summarized data of four independent experiments performed in triplicate are shown as the mean ± SD. **I** Analysis of Foxp3 acetylation. Representative staining results with a FRET antibody pair specific for Foxp3 and AcK in control and Garp-deficient CD4^+^ T cells and a summary of the results from one representative experiment out of four independent experiments with five mice per group. Horizontal lines indicate mean values. AcK acetylated lysin

To gain insights into how GARP might regulate Treg phenotypic stability, we compared the transcriptomes of Garp-deficient and control Tregs (Fig. 6A–E, Supplementary Table 3, Supplementary Fig. 1). Consistent with our findings on apoptosis, significantly diminished expression of Bcl2, encoding an antiapoptotic molecule, but increased expression of Bik, encoding a proapoptotic factor, and several caspase genes were observed in Garp-deficient Tregs (Fig. 6A). The expression of multiple genes involved in regulating the cell cycle was also deregulated in Garp-deficient Tregs (Fig. 6B). Moreover, the expression of the Treg-specific genes Cd25, Nrp1, Ctla4, Il-10, and Hdac9 was diminished, while the expression of several effector cytokines was increased in Garp-deficient Tregs (Fig. 6C, D). Most intriguingly, however, the expression of the Treg-characteristic histone deacetylase Hdac9 was increased, whereas the expression of other histone deacetylases and histone acetyltransferases was not significantly different (Fig. 6E). Hdac9 is a histone deacetylase highly expressed in Tregs. It regulates Treg function and stability by deacetylating Foxp3.^24^ Augmented expression of Hdac9 in Garp-deficient Tregs might therefore lead to Foxp3 instability due to increased Foxp3 deacetylation. The results of a transcriptome analysis for Cd25, Nrp1, Ctla4, and Il-10 were confirmed at the protein level by flow cytometry (Fig. 6F, G), and those for Hdac9 and several other Hdacs were confirmed by PCR analyses (Fig. 6H). We then analyzed the acetylation level of Foxp3 by using a FRET antibody pair specific for Foxp3 and AcK (Fig. 6I). Remarkably, the frequency of Tregs that expressed acetylated Foxp3 was significantly decreased in Garp-deficient Tregs compared to control Tregs, strongly suggesting that the upregulation of Hdac9 results in diminished Foxp3 acetylation.

### GARP controls autocrine TGFβ1 homeostasis in Tregs

**Fig. 7:**
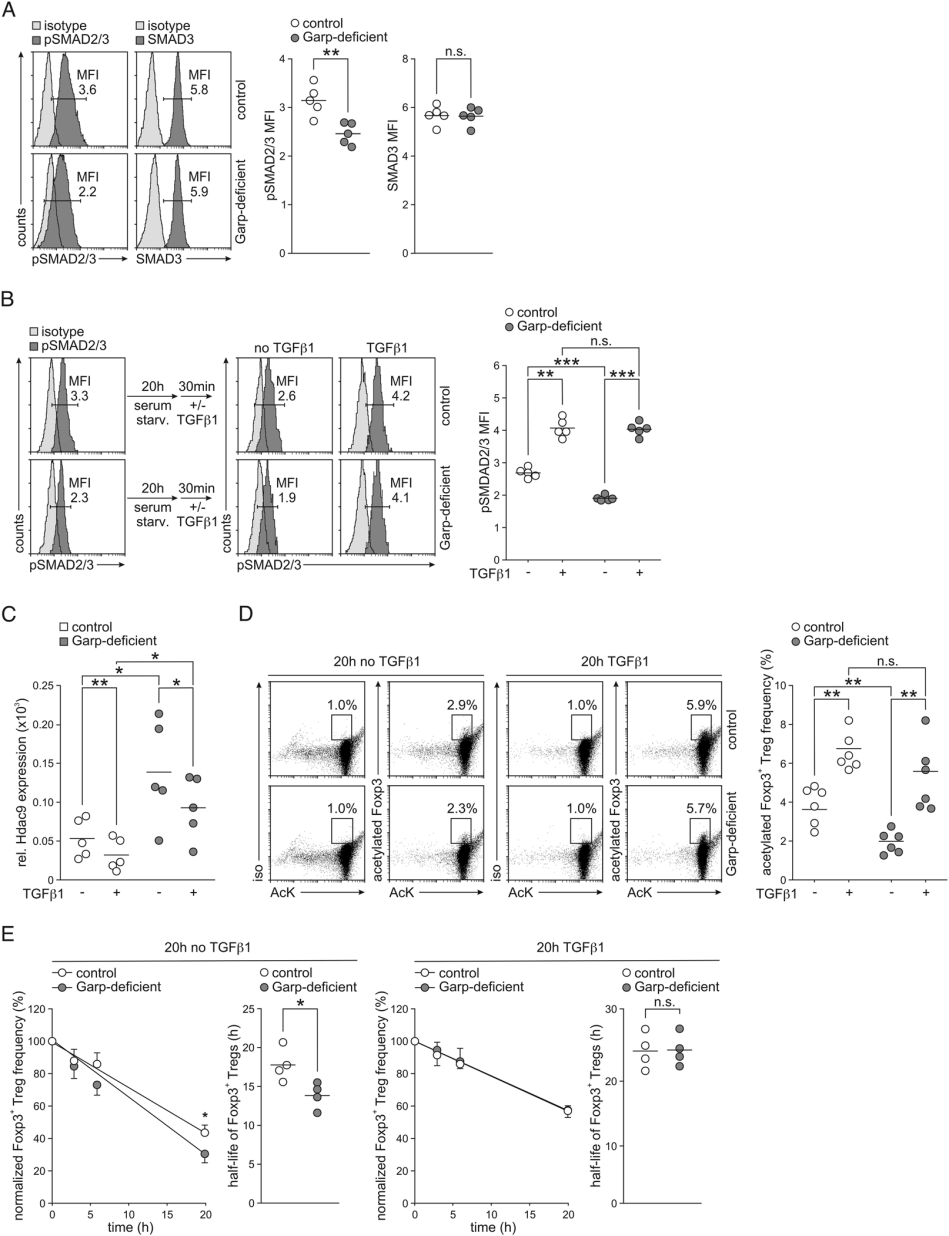
Effect of TGFβ1 on Garp-deficient Tregs. **A** Basal SMAD2/3 phosphorylation and expression in freshly isolated Tregs were determined by intracellular flow cytometry. Representative staining for phosphorylated SMAD2 and SMAD3 (pSMAD2/3) and total SMAD3 in control and Garp-deficient Tregs and a summary of the results from one representative experiment out of two independent experiments with five mice per group. Horizontal lines indicate mean values. MFI mean fluorescence intensity. **B** The pSMAD2/3 level in purified control and Garp-deficient Tregs in response to exogenous TGFβ1 determined by intracellular flow cytometry. The results from one representative experiment and a summary of the results from one representative experiment out of two independent experiments with five mice per group are shown. Horizontal lines indicate mean values. Serum starv. serum starvation, MFI mean fluorescence intensity. **C** The Hdac9 mRNA level in relation to the Actb mRNA level in purified Tregs stimulated with anti-CD3 and anti-CD28 antibodies in the presence or absence of TGFβ1 for 20 h analyzed by real-time PCR. The results of five independent experiments are demonstrated. Horizontal lines indicate mean values. **D**, **E** CD4^+^ T cells from control and Garp-deficient mice were stimulated with anti-CD3 and anti-CD28 antibodies in the presence or absence of TGFβ1 for 20 h. **D** Analysis of Foxp3 acetylation. Representative staining results with a FRET antibody pair specific for Foxp3 and AcK in control and Garp-deficient CD4^+^ T cells and a summary of the results of one representative experiment out of six independent FRET assay experiments with cells from two mice pooled per group. Horizontal lines indicate mean values. **E** Analysis of Treg stability. The frequency of Foxp3^+^ Tregs within CD4^+^ T cells cultured in the absence (left panel) or presence (right panel) of TGFβ1 was assessed by intracellular flow cytometry. CHX was added to T cell cultures after 20 h of incubation and incubated for an additional 20 h. The results of five independent experiments are summarized and shown as the mean ± SD for Treg frequency and as the results of individual experiments with the calculated mean value indicated by the line for the Treg half-life. Linear regression calculated based on normalized frequencies of Foxp3^+^ Tregs cells is shown on the left, and the half-life of Foxp3^+^ Tregs calculated based on linear regression is shown on the right

Given that GARP functions as a receptor of latent TGFβ1, we hypothesized that the decreases in Foxp3 acetylation and stability in Garp-deficient Tregs resulted from the deregulation of TGFβ1 signaling. Indeed, basal phosphorylation of SMAD2 and SMAD3 was significantly diminished in Garp-deficient Tregs compared to control Tregs (Fig. 7A). However, the response of Garp-deficient Tregs to exogenous TGFβ1 was not altered (Fig. 7B). Thus, we treated Garp-deficient Tregs with TGFβ1 for 20 h and analyzed Hdac9 expression and Foxp3 acetylation. Hdac9 expression in Garp-deficient Tregs was significantly reduced in response to TGFβ1 (Fig. 7C). Moreover, treatment with TGFβ1 elevated the frequency of Tregs expressing acetylated Foxp3 in Garp-deficient Tregs to the frequency seen in control Tregs (Fig. 7D). Furthermore, the half-life of Foxp3 in Garp-deficient Tregs was normalized (23.83 ± 2.26 h in Garp-deficient Tregs vs. 23.59 ± 2.31 h in control Tregs, *p* = 0.77) (Fig. 7E, right panel) when cultured in the presence of TGFβ1 prior to CHX incubation, whereas the half-life of Foxp3 in Garp-deficient Tregs cultured in the absence of TGFβ1 remained significantly diminished (17.95 ± 1.28 h vs. 14.33 ± 1.36 h, *p* = 0.016) (Fig. 7E, left panel). We therefore concluded that the lack of GARP expression in Garp-deficient Tregs reduces the TGFβ1 signaling necessary for the formation of a stable Treg phenotype.

### Tregs from patients demonstrate signs of TGFβ1 deprivation

**Fig. 8:**
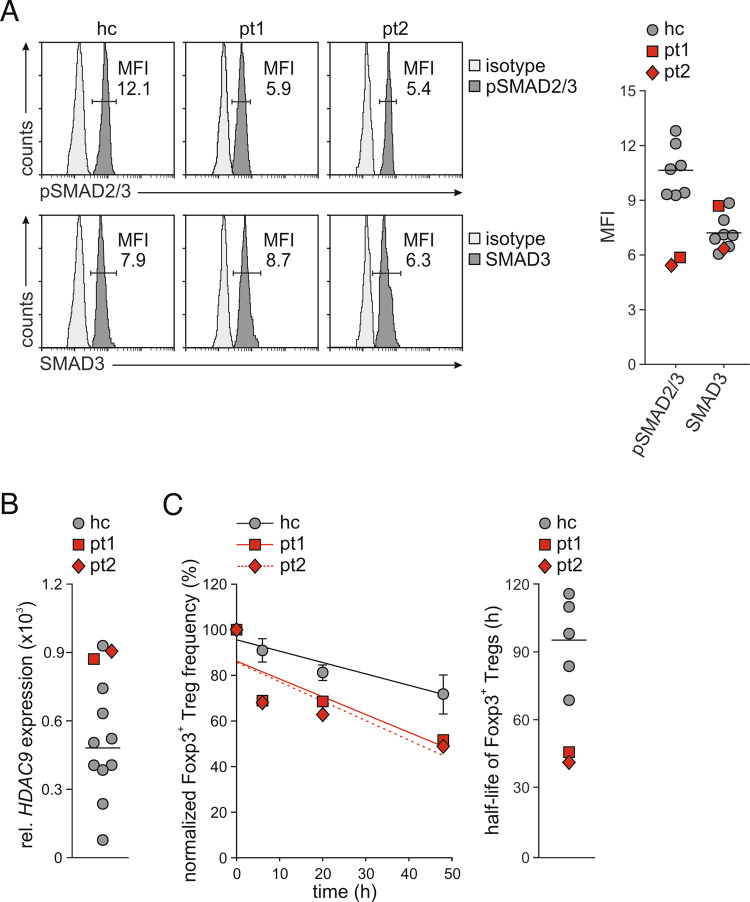
Analysis of SMAD2/3 phosphorylation, *HDAC9* expression, and Treg stability in human Tregs with *LRRC32* mutations. **A** Basal SMAD2/3 phosphorylation and expression in freshly isolated Tregs were determined by intracellular flow cytometry. Representative staining histograms of a healthy control (hc) and both patients are shown. The graph demonstrates the results from the patients in red and the individual results and mean value from seven healthy controls in gray. MFI mean fluorescence intensity. **B** *HDAC9* mRNA expression was assessed in freshly purified Tregs by real-time PCR in duplicate. Relative mRNA expression in relation to the mRNA expression of *ACTB* is shown. The results from the patients are shown in red and those from ten healthy individuals are shown in gray. The horizontal line indicates the mean value of healthy controls. **C** Analysis of Treg stability. The frequency of Foxp3^+^ Tregs within CD4^+^ T cells was assessed in the presence of CHX by intracellular flow cytometry. In the analysis of Treg frequency, gray circles indicate mean values demonstrated as the mean ± SD of five individuals. In the analysis of the Foxp3 half-life, gray circles indicate the individual results of healthy controls, and the horizontal line indicates the mean value of healthy controls. Linear regression calculated based on normalized frequencies of Foxp3^+^ Tregs cells is shown on the left, and the half-life of Foxp3^+^ Tregs calculated based on linear regression is shown on the right

Finally, we analyzed SMAD2/3 phosphorylation in Tregs from the PID patients with mutated *LRRC32*. Similar to Garp-deficient mice, the patients showed a diminished basal level of SMAD2/3 phosphorylation despite normal SMAD3 amounts in Tregs compared to healthy controls (Fig. 8A). Furthermore, Tregs from both patients expressed elevated levels of *HDAC9* (Fig. 8B). Moreover, analysis of Treg stability using CHX revealed that the half-life of Foxp3 in Tregs from pt 1 was 46.28 h and that in Tregs from pt 2 was 41.64 h compared to 95.46 ± 19.13 h in Tregs from healthy controls. Thus, the Foxp3 half-life in Tregs from patients with mutated *LRRC32* was markedly diminished. These observations are consistent with the findings from Garp-deficient mice. They confirmed that diminished GARP expression on the surface of Tregs causes disrupted TGFβ1 homeostasis, leading to Foxp3 instability and Treg dysfunction.

## Discussion

In this study, we demonstrate that diminished GARP expression contributes to immune dysregulation. It is apparent that the immune system of both patients with *LRRC32* mutations cannot properly resolve an immune response. This deficiency led to chronic inflammation, collapse of peripheral tolerance, and disease manifestation. We observed persistent inflammation of the gut mucosa in patient 1 and the development of anti-neutrophilic antibodies and complete elimination of neutrophils in patient 2. Remarkably, in our cohort of PID patients, we identified two patients with mutated *LRRC32*. Whereas c.934C>T and c.1262G>A were previously detected in the general population (Table 1), c.741G>A is a novel mutation. It is also remarkable that the presence of the mutations was found to contribute to a strong decrease in or a complete lack of GARP expression. It is therefore believable that a distinct amount of GARP on the surface of Tregs is necessary to ensure the proper biological function of GARP. In this regard, Nasrallah et al.^25^ reported that the risk locus 11q13.5 associated with immune-mediated diseases contains an enhancer of *LRRC32*. The authors showed that in Tregs, this enhancer forms conformational interactions with the promoter of *LRRC32* and upregulates GARP expression. Several enhancer risk variants were associated with reduced GARP expression. Similar to our finding, the authors concluded that a distinct level of GARP expression is critical for proper Treg function. This conclusion is further supported by our previous observations of a decreased level of GARP on Tregs from patients with rheumatoid arthritis.^26^ Moreover, for atopic dermatitis, a significant excess of low-frequency missense mutations in *LRRC32* leading to low GARP expression has been reported.^27^ Finally, it has been shown that Garp-deficient Tregs facilitate antitumor immunity but are unable to suppress pathogenic T cell responses in a T cell transfer model of colitis, pointing once again to impaired suppressive Treg function in the absence of GARP.^28^

Interestingly, both patients developed immune dysregulation disorders not in childhood but rather at ages resembling the age of onset of many autoinflammatory diseases. In a pathologic adult autoinflammatory situation, it is supposed that an environmental factor triggers disease development in individuals with a genetic susceptibility to the disorder. Similar situations are conceivable for both PID patients. In this regard, naive T cells from both patients appeared to be less proliferative than naive T cells from healthy controls (Fig. 4E). We conclude that this might be an indication of disease chronification, where perpetuated autoinflammation during life impacts the activation of bystander naive T cells. This assumption is supported by the findings from Garp-deficient mice. Garp-deficient mice did not exhibit a spontaneous phenotype but developed vigorous inflammatory disorders in response to a trigger. Our data therefore identify *LRRC32* as a genetic factor determining susceptibility to inflammatory disorders and point to its important function in the integrity of the immune system.

Our data show that the immunological phenotype observed in the absence of GARP occurred due to a reduced availability of TGFβ1 for Tregs. We therefore report for the first time evidence for an autocrine mechanism of action of GARP-bound TGFβ1. Based on previous investigations, it was concluded that GARP-bound TGFβ1 facilitates the development of other Tregs or Th17 cells in a paracrine manner rather than being utilized by Tregs in an autocrine manner.^13,17^ This conclusion was generated based on the observations that Garp-deficient mice have a normal frequency of Tregs and that Garp-deficient Tregs exhibit normal suppressive function in vitro.^29^ Moreover, a normal suppressive capacity was observed for Tgfb1-deficient Tregs in vitro.^29,30^ In contrast, analysis of the suppressive capacity of Tgfb1- and Garp-deficient Tregs in vivo revealed their inability to inhibit colitis in a transfer model of inflammatory bowel disease.^28,31^ Consistently, we observed an apparently normal suppressive capacity for Garp-deficient Tregs in vitro but insufficient control of effector T cell expansion after adoptive transfer in vivo.

Marked alterations in the gene expression profile are the most impressive indication of how important GARP, and consequently GARP-associated TGFβ1, is for Tregs. Among genes with altered expression, Hdac9 is especially notable. HDAC9 is distinctively expressed in Tregs and regulates Foxp3 acetylation.^24^ Acetylation of Foxp3 prevents its polyubiquitination and proteasomal degradation, thus maintaining Foxp3 protein stability.^32^ In addition, acetylation of Foxp3 is necessary for its transcriptional suppressor function.^33^ We propose therefore that in Garp-deficient Tregs, in the presence of abundant HDAC9, Foxp3 becomes less acetylated and therefore less stable. Using FRET analysis, in which the fluorescence signal for Foxp3 could be detected by flow cytometry only if an acetyl group-binding antibody and an anti-Foxp3 antibody bound to their targets in immediate proximity, we observed diminished Foxp3 acetylation in Garp-deficient Tregs. In experiments with CHX, the diminished half-life of Foxp3 in Garp-deficient Tregs was confirmed. Both the diminished level of Foxp3 acetylation and the diminished Foxp3 half-life could be restored by exogenous TGFβ1. Furthermore, the abundant Hdac9 mRNA expression was markedly reduced by exogenous TGFβ1.

Destabilization of Garp-deficient Tregs through reduced Foxp3 acetylation is probably the central event determining the unstable phenotype of Garp-deficient Tregs. The development of Tregs occurs during thymopoiesis and relies on the recognition of self-antigens presented by thymic medullary antigen-presenting cells in the context of MHC class II molecules.^34–36^ Considering that Tregs express potentially self-reactive TCRs, it is conceivable that reduced Foxp3 expression might have harmful consequences for the host. Indeed, several studies have demonstrated that the downregulation or loss of Foxp3 is associated with a loss of the regulatory phenotype and acquisition of an effector phenotype in Tregs.^37–39^ In Garp-deficient Tregs, the expression of all cytokine types, including the Th1, Th2, and Th17 types, was increased, indicating a general deregulation of cytokine expression resulting from diminished Foxp3-mediated transcriptional repression. Interestingly, the phenotype of Garp-deficient Tregs resembles the phenotype of Tregs from mice with genetically engineered attenuated Foxp3 expression.^40^ Due to decreased Foxp3 expression, Tregs from these animals exhibit a reduced regulatory function and express effector cytokines. Our findings are therefore in line with the hypothesis that a certain level of Foxp3 is necessary for the maturation of Tregs with a stable phenotype. It has been shown that mice prone to lupus nephritis exhibit Treg instability due to diminished Foxp3 expression.^41^ Moreover, associations between diminished Foxp3 expression and human immune disorders have been shown.^42,43^ Thus, taken together, our data demonstrate that in the absence of GARP, Tregs develop signs of TGFβ1 deprivation characterized by increased expression of Hdac9 and subsequent destabilization of Foxp3, resulting from reduced acetylation. GARP therefore plays a central role in the stabilization of the immune system.

## Supplementary information

Suppl. Figure 1

Suppl. Table 1

Suppl. Table 2

Suppl. Table 3

## References

[1] Bousfiha A (2018). The 2017 IUIS phenotypic classification for primary immunodeficiencies. J. Clin. Immunol..

[2] Lehman HK (2015). Autoimmunity and immune dysregulation in primary immune deficiency disorders. Curr. Allergy Asthma Rep..

[3] Bennett CL (2001). The immune dysregulation, polyendocrinopathy, enteropathy, X-linked syndrome (IPEX) is caused by mutations of FOXP3. Nat. Genet..

[4] Kuehn HS (2014). Immune dysregulation in human subjects with heterozygous germline mutations in CTLA4. Science.

[5] Schubert D (2014). Autosomal dominant immune dysregulation syndrome in humans with CTLA4 mutations. Nat. Med..

[6] Sakaguchi S (2001). Immunologic tolerance maintained by CD25+ CD4+ regulatory T cells: their common role in controlling autoimmunity, tumor immunity, and transplantation tolerance. Immunol. Rev..

[7] Shevach EM (2002). CD4+ CD25+ suppressor T cells: more questions than answers. Nat. Rev. Immunol..

[8] Brunkow ME (2001). Disruption of a new forkhead/winged-helix protein, scurfin, results in the fatal lymphoproliferative disorder of the scurfy mouse. Nat. Genet.

[9] Hori S, Nomura T, Sakaguchi S (2003). Control of regulatory T cell development by the transcription factor Foxp3. Science.

[10] Fontenot JD, Gavin MA, Rudensky AY (2003). Foxp3 programs the development and function of CD4+CD25+ regulatory T cells. Nat. Immunol..

[11] Tran DQ (2009). GARP (LRRC32) is essential for the surface expression of latent TGF-beta on platelets and activated FOXP3+ regulatory T cells. Proc. Natl Acad. Sci. USA.

[12] Wang R (2009). Expression of GARP selectively identifies activated human FOXP3+ regulatory T cells. Proc. Natl Acad. Sci. USA.

[13] Stockis J, Colau D, Coulie PG, Lucas S (2009). Membrane protein GARP is a receptor for latent TGF-beta on the surface of activated human Treg. Eur. J. Immunol..

[14] Ollendorff V, Noguchi T, deLapeyriere O, Birnbaum D (1994). The GARP gene encodes a new member of the family of leucine-rich repeat-containing proteins. Cell Growth Differ..

[15] Edwards JP, Thornton AM, Shevach EM (2014). Release of active TGF-beta1 from the latent TGF-beta1/GARP complex on T regulatory cells is mediated by integrin beta8. J. Immunol..

[16] Wang R (2012). GARP regulates the bioavailability and activation of TGFbeta. Mol. Biol. Cell.

[17] Cuende J (2015). Monoclonal antibodies against GARP/TGF-beta1 complexes inhibit the immunosuppressive activity of human regulatory T cells in vivo. Sci. Transl. Med..

[18] Vereb G, Nagy P, Szollosi J (2011). Flow cytometric FRET analysis of protein interaction. Methods Mol. Biol..

[19] Bleich A (2004). Refined histopathologic scoring system improves power to detect colitis QTL in mice. Mamm. Genome.

[20] Skapenko A (2004). Generation and regulation of human Th1-biased immune responses in vivo: a critical role for IL-4 and IL-10. J. Immunol..

[21] Lienart S (2018). Structural basis of latent TGF-beta1 presentation and activation by GARP on human regulatory T cells. Science.

[22] Quan L, Lv Q, Zhang Y (2016). STRUM: structure-based prediction of protein stability changes upon single-point mutation. Bioinformatics.

[23] Zhou AX, Kozhaya L, Fujii H, Unutmaz D (2013). GARP-TGF-beta complexes negatively regulate regulatory T cell development and maintenance of peripheral CD4+ T cells in vivo. J. Immunol..

[24] Tao R (2007). Deacetylase inhibition promotes the generation and function of regulatory T cells. Nat. Med..

[25] Nasrallah R (2020). A distal enhancer at risk locus 11q13.5 promotes suppression of colitis by Treg cells. Nature.

[26] Haupt S (2016). Methylation of an intragenic alternative promoter regulates transcription of GARP. Biochim. Biophys. Acta.

[27] Manz J (2016). Targeted resequencing and functional testing identifies low-frequency missense variants in the gene encoding GARP as significant contributors to atopic dermatitis risk. J. Investig. Dermatol..

[28] Salem M (2019). GARP dampens cancer immunity by sustaining function and accumulation of regulatory T cells in the colon. Cancer Res.

[29] Edwards JP (2013). Regulation of the expression of GARP/latent TGF-beta1 complexes on mouse T cells and their role in regulatory T cell and Th17 differentiation. J. Immunol..

[30] Piccirillo CA (2002). CD4(+)CD25(+) regulatory T cells can mediate suppressor function in the absence of transforming growth factor beta1 production and responsiveness. J. Exp. Med..

[31] Li MO, Wan YY, Flavell RA (2007). T cell-produced transforming growth factor-beta1 controls T cell tolerance and regulates Th1- and Th17-cell differentiation. Immunity.

[32] van Loosdregt J (2010). Regulation of Treg functionality by acetylation-mediated Foxp3 protein stabilization. Blood.

[33] Li B (2007). FOXP3 interactions with histone acetyltransferase and class II histone deacetylases are required for repression. Proc. Natl Acad. Sci. USA.

[34] Apostolou I, Sarukhan A, Klein L, von Boehmer H (2002). Origin of regulatory T cells with known specificity for antigen. Nat. Immunol..

[35] Jordan MS (2001). Thymic selection of CD4+CD25+ regulatory T cells induced by an agonist self-peptide. Nat. Immunol..

[36] Aschenbrenner K (2007). Selection of Foxp3+ regulatory T cells specific for self antigen expressed and presented by Aire+ medullary thymic epithelial cells. Nat. Immunol..

[37] Komatsu N (2009). Heterogeneity of natural Foxp3+ T cells: a committed regulatory T-cell lineage and an uncommitted minor population retaining plasticity. Proc. Natl Acad. Sci. USA.

[38] Tsuji M (2009). Preferential generation of follicular B helper T cells from Foxp3+ T cells in gut Peyer’s patches. Science.

[39] Miyara M (2009). Functional delineation and differentiation dynamics of human CD4+ T cells expressing the FoxP3 transcription factor. Immunity.

[40] Wan YY, Flavell RA (2007). Regulatory T-cell functions are subverted and converted owing to attenuated Foxp3 expression. Nature.

[41] Depis F, Kwon HK, Mathis D, Benoist C (2016). Unstable FoxP3+ T regulatory cells in NZW mice. Proc. Natl Acad. Sci. USA.

[42] Balandina A (2005). Functional defect of regulatory CD4(+)CD25+ T cells in the thymus of patients with autoimmune myasthenia gravis. Blood.

[43] Huan J (2005). Decreased FOXP3 levels in multiple sclerosis patients. J. Neurosci. Res..

