# Supplementary figures and images for: Dysregulated immunity in PID patients with low GARP expression on Tregs due to mutations in LRRC32
Source: Cell Mol Immunol. 2021 May 31;18(7):1677–91. doi: 10.1038/s41423-021-00701-z (PMC8245512; doi:10.1038/s41423-021-00701-z)

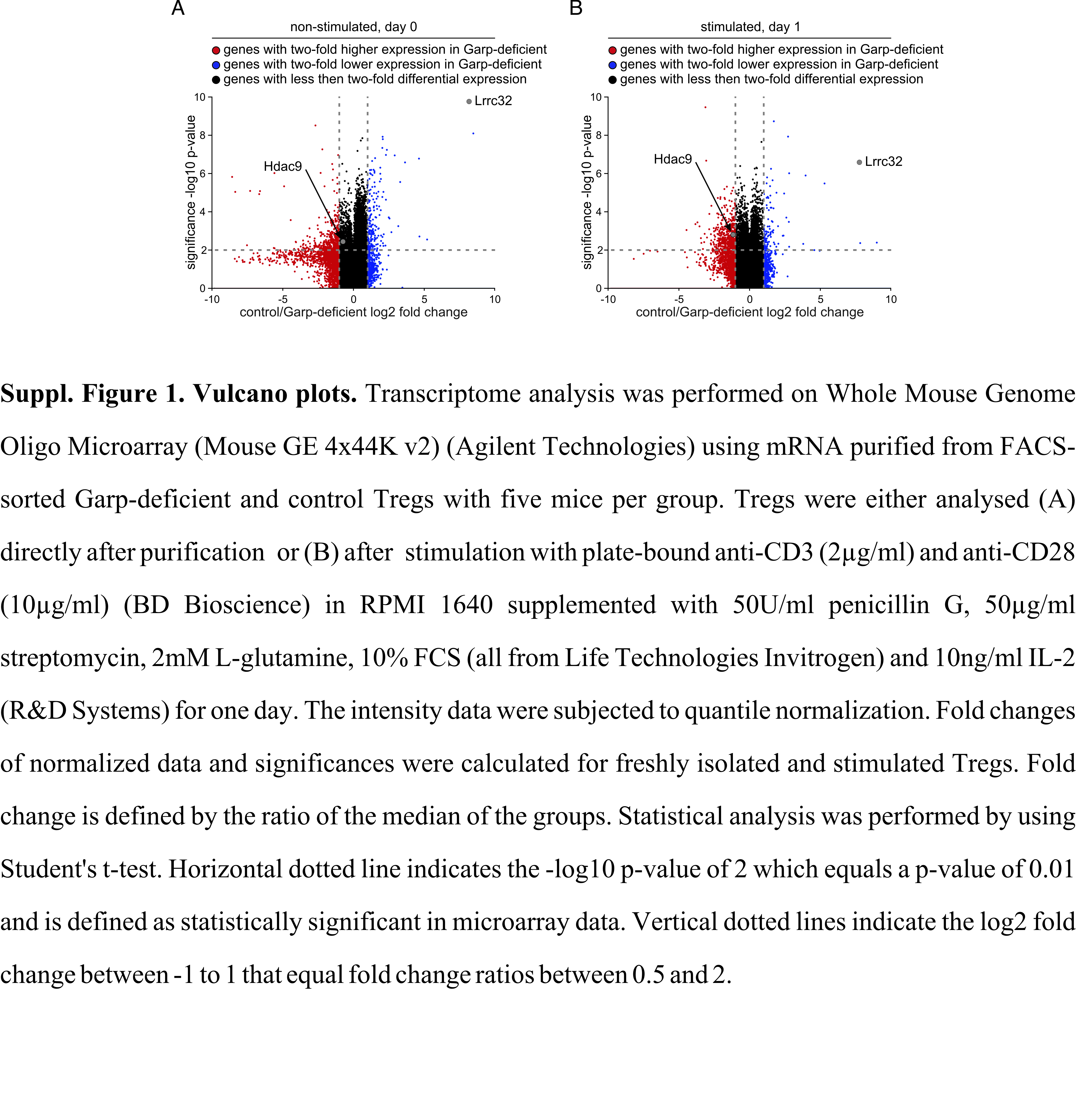

Supplement: Supplementary file 1 — Suppl. Figure 1 [file 41423_2021_701_MOESM1_ESM.tif]
