# Supplementary material for: Dysregulated immunity in PID patients with low GARP expression on Tregs due to mutations in LRRC32
Source: Cell Mol Immunol. 2021 May 31;18(7):1677–91. doi: 10.1038/s41423-021-00701-z (PMC8245512; doi:10.1038/s41423-021-00701-z)
Supplement: Supplementary file 4 — Suppl. Table 3 [file 41423_2021_701_MOESM4_ESM.pdf]

SeqID	ProbeID	GeneName	Description
2271329	A_55_P2031466	Lrrc32	Mus musculus leucine rich repeat containing 3
2271288	A_55_P1985422	LOC100044227	Q811B0_MOUSE (Q811B0) Erythroid differen
2292890	A_55_P2034481	Erdr1	Mus musculus erythroid differentiation regulat
2290610	A_51_P349213	Fcrl1	Mus musculus Fc receptor-like 1 (Fcrl1), transc
2298425	A_51_P501844	Cyp26b1	Mus musculus cytochrome P450, family 26, su
2264815	A_55_P2062133	Etv3	Mus musculus ets variant gene 3 (Etv3), trans
2295558	A_30_P01027087	A_30_P01027087	lincRNA:chr3:79069438-79073704 reverse str
2285809	A_55_P2016166	Wfdc2	Mus musculus WAP four-disulfide core domain
2306135	A_30_P01021230	A_30_P01021230	lincRNA:chr3:37659958-37665945 forward st
2264323	A_52_P512427	2510049J12Rik	Mus musculus RIKEN cDNA 2510049J12 gen
2310774	A_55_P2107775	Apol9a	Mus musculus apolipoprotein L 9a (Apol9a), t
2286413	A_66_P128761	E430029J22Rik	Mus musculus pyrin domain containing 3 (Pyd
2276525	A_55_P1953341	Wfdc2	Mus musculus WAP four-disulfide core domain
2261113	A_55_P1967286	Gm1409	immunoglobulin kappa variable 5-39 [Source:
2286414	A_30_P01025525	A_30_P01025525	lincRNA:chr3:37659958-37665945 forward st
2275136	A_55_P2144686	Dmbt1	Mus musculus deleted in malignant brain tum
2259608	A_55_P2023637	Prg4	Mus musculus proteoglycan 4 (megakaryocyte
2282501	A_55_P2110136	Arhgap29	Mus musculus Rho GTPase activating protein
2263575	A_55_P2043367	Apol9a	Mus musculus apolipoprotein L 9a (Apol9a), t
2306577	A_66_P110633	Apol9b	Mus musculus apolipoprotein L 9b (Apol9b), t
2304380	A_30_P01024788	A_30_P01024788	lincRNA:chr3:79069438-79073704 reverse st
2307570	A_55_P2181222	E430029J22Rik	Mus musculus pyrin domain containing 3 (Pyd
2283521	A_52_P481279	Gm1060	Mus musculus coiled-coil domain containing 1
2300377	A_55_P1958018	LOC100047337	Q6EE68_PRODO (Q6EE68) Ribosomal prote
2292666	A_55_P1960894	Cabp1	Mus musculus calcium binding protein 1 (Cab
2286467	A_55_P2056192	NAP111293-1	Unknown
2296377	A_55_P2137828	Gprc5b	Mus musculus G protein-coupled receptor, fa
2295324	A_55_P2163098	Akr1c18	Mus musculus aldo-keto reductase family 1, m
2273936	A_55_P2022971	Tspan9	Mus musculus tetraspanin 9 (Tspan9), mRNA
2273566	A_30_P01025989	A_30_P01025989	lincRNA:chr3:84609788-84618062 reverse st
2310092	A_52_P880457	Ampd1	Mus musculus adenosine monophosphate de
2293698	A_55_P1973451	Stra6	Mus musculus stimulated by retinoic acid gen
2304572	A_30_P01030239	A_30_P01030239	lincRNA:chr13:28506971-28511732 reverse s
2264160	A_51_P466685	Fbxl22	Mus musculus F-box and leucine-rich repeat p
2289880	A_30_P01028589	A_30_P01028589	lincRNA:chr7:134835448-134842613 forward
2288947	A_55_P2050426	Ankrd50	Mus musculus ankyrin repeat domain 50 (Ank
2294793	A_55_P1962603	Cplx2	Mus musculus complexin 2 (Cplx2), mRNA [N
2259768	A_55_P2024634	Gm13546	predicted gene 13546 [Source:MGI Symbol;A
2286112	A_52_P588483	Fbln1	Mus musculus fibulin 1 (Fbln1), mRNA [NM_01
2295753	A_66_P105075	Mmp11	Mus musculus matrix metallopeptidase 11 (Mr
2259463	A_51_P268069	Six1	Mus musculus sine oculis-related homeobox 1
2287192	A_52_P382802	1700102P08Rik	Mus musculus RIKEN cDNA 1700102P08 ge
2267405	A_52_P257502	Igfbp4	Mus musculus insulin-like growth factor bindin
2285998	A_55_P2148400	Gm2264	Unknown
2296525	A_30_P01022835	A_30_P01022835	lincRNA:chr3:106858397-106896812 reverse
2290988	A_55_P2058297	Sgip1	Mus musculus SH3-domain GRB2-like (endop

2263864	A_52_P646783	Fbxl22	Mus musculus F-box and leucine-rich repeat p
2286408	A_30_P01024831	A_30_P01024831	lincRNA:chr8:19678750-20038750 forward st
2284827	A_51_P218682	4930588N13Rik	Mus musculus RIKEN cDNA 4930588N13 ger
2309983	A_55_P2183914	Gm7120	Mus musculus predicted gene 7120 (Gm7120
2276254	A_51_P114826	Cdh13	Mus musculus cadherin 13 (Cdh13), mRNA [N
2297654	A_55_P1962602	Gm2690	Mus musculus 2 days neonate sympathetic ga
2266301	A_55_P1989185	ENSMUST0000010	PREDICTED: Mus musculus predicted gene 6
2295189	A_52_P227267	Atp1a2	Mus musculus ATPase, Na+/K+ transporting,
2287554	A_51_P398766	Gbp1	Mus musculus guanylate binding protein 1 (G
2289922	A_55_P2070441	Sp6	Mus musculus trans-acting transcription factor
2264259	A_55_P2001233	E430029J22Rik	Mus musculus pyrin domain containing 3 (Pyd
2272018	A_55_P1966383	Gm2936	Unknown
2292116	A_55_P1964433	Zmynd12	Mus musculus zinc finger, MYND domain cont
2297550	A_55_P2044582	Iglon5	Mus musculus IgLON family member 5 (Iglon5
2292336	A_55_P2072995	ENSMUST0000010	immunoglobulin kappa variable 11-125 [Sourc
2268697	A_66_P121480	Sardh	Mus musculus sarcosine dehydrogenase (Sar
2290219	A_55_P2024669	Myo6	Mus musculus myosin VI (Myo6), mRNA [NM_
2283443	A_51_P290576	Plk2	Mus musculus polo-like kinase 2 (Drosophila)
2261730	A_55_P2176280	Prkar1b	Mus musculus protein kinase, cAMP depende
2267880	A_55_P2040775	Gm6837	Mus musculus 921-L mRNA for presynaptic pr
2298126	A_55_P2074736	Prkar1b	Mus musculus protein kinase, cAMP depende
2301495	A_52_P52946	4922501L14Rik	Mus musculus RIKEN cDNA 4922501L14 ger
2311237	A_51_P211854	Selp	Mus musculus selectin, platelet (Selp), mRNA
2289499	A_51_P258848	4933427G17Rik	Mus musculus RIKEN cDNA 4933427G17 ger
2303022	A_30_P01020912	A_30_P01020912	lincRNA:chr10:3231337-3231802 forward stra
2297898	A_52_P344152	Pik3ip1	phosphoinositide-3-kinase interacting protein
2312005	A_55_P1991770	Pdlim4	Mus musculus PDZ and LIM domain 4 (Pdlim4
2257498	A_51_P131408	Tnfrsf12a	Mus musculus tumor necrosis factor receptor s
2260729	A_55_P2148402	Cplx2	Mus musculus complexin 2 (Cplx2), mRNA [NM
2302158	A_66_P121965	1110032F04Rik	Mus musculus RIKEN cDNA 1110032F04 ger
2263644	A_52_P325443	Arhgap29	Mus musculus Rho GTPase activating protein
2278358	A_51_P214470	2610019F03Rik	Mus musculus RIKEN cDNA 2610019F03 ger
2281640	A_30_P01020388	A_30_P01020388	lincRNA:chrX:47890565-47915775 forward st
2303536	A_55_P2028576	Gm7057	Unknown
2303879	A_55_P1989698	Mmp11	Mus musculus matrix metallopeptidase 11 (Mr
2281996	A_55_P2026982	2610019F03Rik	Mus musculus RIKEN cDNA 2610019F03 ger
2265778	A_51_P258409	Hey1	Mus musculus hairy/enhancer-of-split related w
2270098	A_55_P2064351	Vipr1	Mus musculus vasoactive intestinal peptide re
2310090	A_52_P900300	Gm6213	Mus musculus predicted gene 6213 (Gm6213
2272724	A_51_P293938	Rasl11b	Mus musculus RAS-like, family 11, member B
2289457	A_55_P2180445	Gm2742	Unknown
2298235	A_55_P2056533	Ntrk3	Mus musculus neurotrophic tyrosine kinase, re
2270946	A_66_P122621	ENSMUST0000006	Mus musculus 16 days neonate thymus cDNA
2303029	A_55_P2120866	LOC100044171	PREDICTED: Mus musculus hypothetical LOC
2300864	A_30_P01018048	A_30_P01018048	lincRNA:chr3:37659958-37665945 forward st
2302642	A_52_P202142	Sv2a	Mus musculus synaptic vesicle glycoprotein 2
2300019	A_52_P316933	Sh3bgrl2	Mus musculus SH3 domain binding glutamic a
2271862	A_55_P1968085	A_55_P1968085	Unknown
2257317	A_51_P391955	Dapl1	Mus musculus death associated protein-like 1
2271589	A_51_P349413	ENSMUST0000010	ADP-ribosylation factor-like 5C [Source:MGI S
2285704	A_55_P1997651	Espn	Mus musculus espin (Espn), transcript variant
2282251	A_55_P2384671	AK033123	predicted gene, 17641 [Source:MGI Symbol;A
2300919	A_55_P1960043	Sec31b	Mus musculus Sec31 homolog B (S. cerevisia
2299369	A_55_P1992571	Gm2952	predicted gene, 17684 [Source:MGI Symbol;A
2296596	A_55_P2103249	Cxcr1	Mus musculus chemokine (C-X-C motif) recept
2300416	A_55_P2391881	Gm12108	predicted gene 12108 [Source:MGI Symbol;A

2259114	A_55_P2172086	Gm10640	Mus musculus adult male testis cDNA, RIKEN
2280536	A_55_P2115044	LOC100047266	BY110436 RIKEN full-length enriched, 15 day
2269038	A_55_P2416464	4921511I17Rik	Mus musculus adult male testis cDNA, RIKEN
2295625	A_30_P01030370	A_30_P01030370	lincRNA:chr18:35967988-35968812 forward s
2295422	A_55_P2043627	Fam89a	Mus musculus family with sequence similarity 8
2304442	A_66_P138298	Gm4371	Mus musculus predicted gene 4371 (Gm4371
2275740	A_51_P104418	Dusp10	Mus musculus dual specificity phosphatase 10
2256887	A_30_P01027107	A_30_P01027107	lincRNA:chr11:99048975-99062425 reverse s
2297016	A_30_P01020593	A_30_P01020593	lincRNA:chr12:81217572-81233910 forward s
2296384	A_55_P1979003	LOC100044493	Unknown
2272958	A_55_P2041728	Gm590	Mus musculus predicted gene 590 (Gm590), r
2291699	A_30_P01017850	A_30_P01017850	lincRNA:chr5:54053328-54053870 forward st
2270383	A_52_P173442	Wscd2	Mus musculus WSC domain containing 2 (Wsc
2266643	A_55_P2145356	Thada	Mus musculus mRNA for mKIAA1767 protein.
2295557	A_55_P2151521	ENSMUST0000010	microfibrillar-associated protein 3 [Source:MGI
2292718	A_55_P1959639	LOC100045061	Unknown
2307321	A_55_P2054743	Sh3bgrl2	Mus musculus SH3 domain binding glutamic a
2303379	A_55_P2056973	Treml2	Mus musculus triggering receptor expressed o
2303803	A_55_P1999691	Usp27x	ubiquitin specific peptidase 27, X chromosome
2261528	A_55_P2007233	ENSMUST0000006	PREDICTED: Mus musculus Riken cDNA A53
2274870	A_55_P2167521	Gm15503	Mus musculus 16 days neonate thymus cDNA
2306017	A_30_P01025698	A_30_P01025698	lincRNA:chr10:122410471-122422759 revers
2260520	A_55_P1964332	LOC100048239	Unknown
2285616	A_55_P2120650	LOC100047155	Unknown
2297554	A_55_P2003309	Gm2073	Unknown
2298365	A_52_P565575	Arhgap20	Mus musculus Rho GTPase activating protein
2259438	A_55_P1981929	Scg5	Mus musculus secretogranin V (Scg5), mRNA
2302035	A_55_P1953508	Mobkl2c	Mus musculus MOB kinase activator 3C (Mob3
2257397	A_52_P104091	Slc25a27	Mus musculus solute carrier family 25, membe
2300886	A_66_P107483	Treml2	Mus musculus triggering receptor expressed o
2281872	A_51_P305532	Eif2s3x	Mus musculus eukaryotic translation initiation
2260440	A_55_P2170225	Sh3bgrl2	Mus musculus SH3 domain binding glutamic a
2303472	A_66_P111090	Sctr	Mus musculus secretin receptor (Sctr), mRNA
2297274	A_55_P2007234	ENSMUST0000006	PREDICTED: Mus musculus Riken cDNA A53
2289855	A_51_P182728	Stx1a	Mus musculus syntaxin 1A (brain) (Stx1a), mR
2301960	A_30_P01027042	A_30_P01027042	lincRNA:chr11:120041124-120052299 revers
2288869	A_52_P509965	Ager	Mus musculus advanced glycosylation end pr
2270548	A_52_P108850	St8sia1	Mus musculus ST8 alpha-N-acetyl-neuraminid
2281384	A_51_P412225	Tbc1d16	Mus musculus TBC1 domain family, member 1
2291057	A_55_P2045129	Gm4361	Mus musculus adult male thymus cDNA, RIKE
2265157	A_52_P590535	Fbln2	Mus musculus fibulin 2 (Fbln2), transcript varia
2270323	A_30_P01030905	A_30_P01030905	lincRNA:chr15:85300937-85301522 forward s
2291523	A_55_P2022519	Tmem108	Mus musculus transmembrane protein 108 (Tr
2268971	A_55_P1985890	Tiparp	Mus musculus TCDD-inducible poly(ADP-ribos
2280739	A_52_P64687	Camk2n1	Mus musculus calcium/calmodulin-dependent
2261093	A_51_P518051	Dusp4	Mus musculus dual specificity phosphatase 4
2292111	A_30_P01022122	A_30_P01022122	lincRNA:chr8:19678750-20038750 reverse st
2301761	A_52_P283524	Rcan3	Mus musculus regulator of calcineurin 3 (Rcan
2306669	A_30_P01022858	A_30_P01022858	lincRNA:chr4:131737612-131738100 reverse
2264610	A_55_P2286493	4933405E24Rik	Mus musculus RIKEN cDNA 4933405E24 ger
2291561	A_55_P2159850	Gm4191	603275854F1 NCI_CGAP_Mam3 Mus muscu
2264814	A_52_P478394	Dleu7	Mus musculus deleted in lymphocytic leukemia
2309359	A_66_P108003	Espn	Mus musculus espin (Espn), transcript variant
2268077	A_55_P1994309	Ptgir	Mus musculus prostaglandin I receptor (IP) (P
2265319	A_55_P2091601	Col11a2	Mus musculus collagen, type XI, alpha 2 (Col1
2261343	A_55_P2024046	Slc16a5	Mus musculus solute carrier family 16 (monoca

2312367	A_55_P2007205	ENSMUST0000006	predicted pseudogene 10749 [Source:MGI S
2299634	A_55_P2025755	Syce1l	Mus musculus synaptonemal complex central
2296586	A_55_P2113738	Gm4102	AV573893 Abe mouse ES cell Mus musculus
2284432	A_55_P2156905	Sox5	Mus musculus SRY-box containing gene 5 (S
2258300	A_55_P2096121	Gm2528	Unknown
2261843	A_51_P443819	2610034M16Rik	Mus musculus RIKEN cDNA 2610034M16 ge
2292229	A_55_P2004168	LOC100046325	Unknown
2306039	A_55_P2017789	Il4ra	Mus musculus interleukin 4 receptor, alpha (Il
2299042	A_55_P2084890	C2cd2l	Mus musculus C2 calcium-dependent domain
2259334	A_55_P1993858	Rmrp	Mus musculus RNA component of mitochondr
2283668	A_55_P2092156	Gm2911	Unknown
2282383	A_55_P2040774	Gm6837	Unknown
2281140	A_55_P2135526	Gzmc	Mus musculus granzyme C (Gzmc), mRNA [N
2281039	A_30_P01025873	A_30_P01025873	lincRNA:chr10:44707451-44707693 reverse s
2311127	A_55_P2075449	Ttll3	Mus musculus tubulin tyrosine ligase-like famil
2310150	A_55_P1993483	Gm7462	Unknown
2289783	A_55_P2004881	D18Ertd653e	Mus musculus DNA segment, Chr 18, ERATO
2266365	A_55_P2159149	Nsg2	neuron specific gene family member 2 [Sourc
2307063	A_55_P2005720	Gm4253	Unknown
2263518	A_55_P2021476	XM_001474821	Q6TXJ2_RAT (Q6TXJ2) LRRGT00007, partia
2269832	A_55_P2044917	Gpr83	Mus musculus G protein-coupled receptor 83
2305092	A_66_P121583	Gm7120	Mus musculus predicted gene 7120 (Gm7120
2270938	A_30_P01028003	A_30_P01028003	lincRNA:chrX:6950444-6952293 reverse stra
2262314	A_55_P2097072	Gm7219	Unknown
2296484	A_30_P01024498	A_30_P01024498	lincRNA:chr13:28506971-28511732 reverse s
2274282	A_30_P01030979	A_30_P01030979	lincRNA:chr10:120336296-120354471 revers
2260042	A_51_P434758	Fam83e	Mus musculus family with sequence similarity
2288082	A_30_P01027441	A_30_P01027441	lincRNA:chr13:28506971-28511732 reverse s
2279738	A_51_P471791	St8sia6	Mus musculus ST8 alpha-N-acetyl-neuraminid
2276748	A_30_P01021556	A_30_P01021556	lincRNA:chr8:19678750-20038750 forward st
2291650	A_55_P2097219	Gbp1	Mus musculus guanylate binding protein 1 (G
2275270	A_55_P1986680	Gm2334	Unknown
2289249	A_52_P629895	Adh1	Mus musculus alcohol dehydrogenase 1 (clas
2300284	A_30_P01021222	A_30_P01021222	lincRNA:chr17:8718223-8949898 forward stra
2309177	A_55_P2179604	Vash1	Mus musculus vasohibin 1 (Vash1), mRNA [N
2266013	A_30_P01021493	A_30_P01021493	lincRNA:chr3:93564825-93576725 forward st
2294141	A_55_P1954276	Emid1	Mus musculus EMI domain containing 1 (Emid
2280892	A_51_P382369	Zfp608	Mus musculus zinc finger protein 608 (Zfp608
2292220	A_55_P2000354	Rgs11	Mus musculus regulator of G-protein signaling
2275135	A_51_P419389	Bmpr2	Mus musculus bone morphogenic protein rece
2257193	A_55_P2024366	Raver2	Mus musculus ribonucleoprotein, PTB-binding
2278795	A_30_P01031078	A_30_P01031078	lincRNA:chr4:45079022-45096897 reverse st
2292045	A_55_P2007630	Sez6l2	Mus musculus seizure related 6 homolog like
2289113	A_51_P480013	Car11	Mus musculus carbonic anhydrase 11 (Car11)
2286929	A_30_P01020222	A_30_P01020222	lincRNA:chr4:62367014-62386800 forward st
2310472	A_55_P2074045	Gm3655	Q8VKJ6_MYCTU (Q8VKJ6) PE_PGRS family
2258681	A_65_P16059	Tgfbr3	Mus musculus transforming growth factor, bet
2275961	A_52_P546459	Ube2d1	Mus musculus ubiquitin-conjugating enzyme E
2265884	A_30_P01032768	A_30_P01032768	lincRNA:chr11:99048975-99062425 reverse s
2267983	A_55_P2105452	Foxp1	Mus musculus forkhead box P1 (Foxp1), trans
2262856	A_30_P01030720	A_30_P01030720	lincRNA:chr10:62267317-62269871 reverse s
2304915	A_30_P01032149	A_30_P01032149	lincRNA:chr14:73729977-73751877 reverse s
2261471	A_55_P2161400	Sctr	Mus musculus secretin receptor (Sctr), mRNA
2267790	A_51_P363749	Irf6	Mus musculus interferon regulatory factor 6 (I
2309282	A_51_P418809	Airn	Mus musculus antisense Igf2r RNA (Airn), tra
2288204	A_55_P1964004	Gm3521	BY456555 RIKEN full-length enriched, adult

ANOVA p-value	Adjusted ANOVA p-value	Tukey p-value	Fold change	Log2 ratio	Median	wt_non-stim_1	wt_non-stim_2	wt_non-stim_3
1,26E-19	4,38E-16	2,26E-14	-1619,127	-10,661	10,425	4,432	4,248	4,223
4,28E-04	1,39E-03	3,50E-03	-38,479	-5,266	13,324	0,193	-0,022	1,055
4,09E-04	1,34E-03	3,46E-03	-26,373	-4,721	13,815	0,315	-0,030	1,024
1,48E-17	8,83E-15	2,34E-14	-12,915	-3,691	3,846	1,876	1,563	1,550
1,93E-12	5,27E-11	1,73E-10	-9,292	-3,216	2,221	3,218	3,987	3,684
3,93E-16	7,93E-14	4,35E-14	-7,803	-2,964	9,485	1,584	1,704	1,810
7,70E-07	4,13E-06	7,00E-06	-6,648	-2,733	2,511	2,254	2,209	2,529
3,97E-12	9,31E-11	7,09E-08	-6,021	-2,590	3,594	3,414	3,092	3,213
1,62E-08	1,24E-07	1,72E-05	-5,812	-2,539	5,013	0,072	-0,265	-0,566
4,68E-06	2,16E-05	2,25E-03	-5,724	-2,517	4,800	-0,144	-1,212	-0,286
1,84E-11	3,36E-10	4,44E-09	-5,307	-2,408	6,844	1,812	2,027	1,884
8,36E-17	2,89E-14	9,71E-09	-5,077	-2,344	6,113	3,829	3,835	3,883
9,68E-14	4,65E-12	5,84E-09	-5,049	-2,336	4,732	2,696	2,732	2,887
1,59E-07	9,82E-07	1,85E-02	-4,928	-2,301	8,962	4,887	5,658	4,321
1,39E-07	8,68E-07	2,51E-05	-4,510	-2,173	3,768	0,072	-0,483	
1,72E-04	6,02E-04	5,47E-04	-4,478	-2,163	2,008	2,029	2,470	0,058
2,20E-12	5,81E-11	5,26E-07	-4,463	-2,158	3,934	3,659	3,580	4,095
2,50E-04	8,48E-04	1,96E-03	-4,365	-2,126	3,462	1,313	-0,075	1,023
1,19E-11	2,32E-10	3,11E-09	-4,353	-2,122	9,361	2,081	2,086	2,083
1,19E-11	2,32E-10	3,75E-09	-4,296	-2,103	9,841	2,002	2,060	2,088
1,10E-05	4,74E-05	3,14E-04	-4,272	-2,095	2,907	1,495	2,006	2,407
2,63E-16	6,03E-14	2,67E-06	-4,243	-2,085	9,146	4,039	3,987	4,053
1,74E-14	1,27E-12	1,35E-08	-4,228	-2,080	8,074	2,634	2,887	2,792
1,45E-07	9,02E-07	3,53E-04	-3,992	-1,997	2,679	2,779	2,217	2,694
1,00E-03	3,06E-03	9,18E-04	-3,972	-1,990	3,412	0,945	0,036	1,155
1,46E-10	1,98E-09	1,63E-09	-3,945	-1,980	7,163	0,475	0,705	0,782
1,58E-06	7,97E-06	3,32E-02	-3,942	-1,979	3,321	1,848	3,695	3,095
3,71E-17	1,73E-14	1,89E-09	-3,910	-1,967	8,436	-1,877	-2,071	-2,055
7,50E-08	4,96E-07	1,81E-06	-3,834	-1,939	4,084	0,228	0,487	0,607
8,36E-06	3,68E-05	3,49E-03	-3,832	-1,938	4,015	0,340	0,343	-0,219
1,26E-13	5,75E-12	2,60E-06	-3,821	-1,934	4,243	3,759	3,667	3,630
2,14E-06	1,05E-05	3,19E-06	-3,750	-1,907	6,794	0,818	0,680	0,557
2,57E-06	1,25E-05	1,50E-04	-3,696	-1,886	3,097	2,156	2,245	2,131
2,43E-07	1,45E-06	8,32E-05	-3,638	-1,863	2,880	2,528	2,192	1,948
1,96E-02	4,66E-02	1,92E-02	-3,613	-1,853	2,982	4,146	1,849	0,024
4,24E-03	1,15E-02	2,74E-02	-3,543	-1,825	8,481	0,113	0,106	-0,022
7,27E-06	3,24E-05	2,54E-03	-3,543	-1,825	10,029	-1,074	-0,353	-1,383
3,78E-07	2,16E-06	4,85E-06	-3,489	-1,803	4,748	0,311	0,390	0,774
1,41E-06	7,18E-06	8,01E-04	-3,485	-1,801	4,788	-0,539	-0,171	-0,258
8,84E-10	9,45E-09	1,91E-07	-3,392	-1,762	5,941	1,881	1,358	1,877
2,94E-10	3,63E-09	9,36E-04	-3,313	-1,728	5,290	-1,369	-1,225	-1,270
3,91E-05	1,53E-04	1,13E-03	-3,310	-1,727	2,032	1,808	1,980	2,248
1,98E-09	1,92E-08	2,08E-05	-3,294	-1,720	6,800	2,729	2,581	2,278
1,44E-05	6,06E-05	3,24E-03	-3,272	-1,710	9,924	-0,956	-0,182	-1,445
5,77E-06	2,62E-05	4,27E-03	-3,245	-1,698	2,346	1,780	1,025	2,005
1,75E-03	5,10E-03	2,89E-02	-3,227	-1,690	3,277	1,515	1,412	1,119

1,12E-09	1,16E-08	2,12E-05	-3,215	-1,685	4,386	2,639	2,319	2,347
2,46E-03	6,99E-03	1,80E-03	-3,198	-1,677	3,530	0,821	0,819	0,099
1,86E-05	7,68E-05	8,25E-04	-3,191	-1,674	4,025	1,682	1,888	1,748
1,52E-06	7,70E-06	3,93E-03	-3,149	-1,655	5,676	0,025	0,155	0,232
1,16E-08	9,24E-08	3,33E-03	-3,130	-1,646	3,827	2,447	2,435	2,661
1,80E-06	8,97E-06	2,86E-03	-3,125	-1,644	8,996	-1,415	-0,701	-1,711
2,78E-05	1,11E-04	4,10E-04	-3,084	-1,625	2,227	2,668	0,588	1,389
5,41E-08	3,68E-07	4,33E-05	-3,065	-1,616	3,016	1,533	0,196	0,764
6,44E-13	2,15E-11	2,84E-05	-3,021	-1,595	3,290	2,587	2,808	2,435
8,60E-05	3,17E-04	8,20E-04	-3,017	-1,593	6,715	1,437	1,130	1,494
7,51E-16	1,24E-13	6,62E-08	-2,971	-1,571	9,641	3,474	3,498	3,579
1,53E-07	9,48E-07	2,01E-03	-2,949	-1,560	8,837	-1,217	-0,421	-1,451
4,71E-05	1,82E-04	4,09E-04	-2,944	-1,558	4,599	0,976	1,737	1,094
3,55E-05	1,40E-04	2,04E-03	-2,940	-1,556	4,840	1,659	1,691	1,440
1,85E-07	1,13E-06	9,67E-03	-2,934	-1,553	4,147	2,259	2,794	2,207
2,01E-09	1,94E-08	2,55E-05	-2,928	-1,550	6,039	1,145	1,349	1,207
2,68E-03	7,56E-03	6,66E-03	-2,906	-1,539	4,888	1,321	1,532	1,824
3,32E-06	1,58E-05	1,16E-04	-2,902	-1,537	6,898	0,217	0,070	0,207
1,38E-03	4,10E-03	3,36E-03	-2,890	-1,531	3,292	1,572	2,781	1,242
2,43E-07	1,45E-06	3,59E-04	-2,890	-1,531	12,796	-0,988	-0,510	-1,448
5,19E-06	2,38E-05	1,68E-04	-2,888	-1,530	7,097	1,176	0,993	0,986
5,00E-08	3,43E-07	1,62E-06	-2,888	-1,530	2,004	1,991	1,679	1,957
2,88E-08	2,08E-07	9,11E-07	-2,886	-1,529	4,472	1,493	1,330	1,345
1,43E-03	4,23E-03	4,72E-02	-2,860	-1,516	3,008	1,462	0,982	1,296
1,35E-04	4,80E-04	1,03E-02	-2,850	-1,511	2,103	1,182	1,902	2,146
6,96E-14	3,67E-12	1,74E-05	-2,823	-1,497	6,044	3,405	3,507	3,360
2,08E-09	2,00E-08	2,81E-04	-2,795	-1,483	8,519	2,968	3,089	2,930
1,03E-06	5,40E-06	7,58E-07	-2,766	-1,468	9,298	1,154	1,093	1,018
5,21E-07	2,89E-06	8,41E-04	-2,749	-1,459	12,123	-1,073	-0,508	-1,528
1,70E-12	4,76E-11	1,11E-07	-2,747	-1,458	10,810	-0,604	-0,594	-0,504
7,42E-03	1,92E-02	9,17E-03	-2,732	-1,450	3,334	1,213	0,289	0,848
6,54E-06	2,94E-05	1,68E-03	-2,732	-1,450	5,541	1,800	1,622	1,641
3,51E-03	9,70E-03	2,02E-02	-2,730	-1,449	4,947	0,262	-0,087	0,087
4,82E-07	2,70E-06	8,60E-04	-2,709	-1,438	10,977	-0,928	-0,339	-1,384
2,47E-08	1,81E-07	2,08E-06	-2,700	-1,433	7,365	1,622	1,331	1,780
1,39E-08	1,08E-07	1,22E-04	-2,691	-1,428	9,089	1,922	2,056	2,136
1,36E-10	1,86E-09	9,28E-03	-2,685	-1,425	5,423	3,104	3,056	2,865
5,17E-09	4,48E-08	2,39E-04	-2,680	-1,422	10,036	2,238	2,135	2,234
1,11E-08	8,88E-08	3,91E-04	-2,678	-1,421	2,311	3,464	3,021	3,313
1,13E-06	5,87E-06	2,75E-04	-2,676	-1,420	7,832	1,676	1,529	1,785
2,29E-07	1,37E-06	7,85E-04	-2,674	-1,419	9,061	-1,056	-0,388	-1,423
3,09E-04	1,03E-03	1,31E-02	-2,648	-1,405	3,147	1,085	1,644	1,105
3,55E-15	3,84E-13	2,60E-03	-2,646	-1,404	4,713	3,851	3,829	3,492
1,75E-08	1,33E-07	1,19E-04	-2,635	-1,398	6,496	0,013	0,001	0,310
2,96E-12	7,36E-11	6,39E-08	-2,603	-1,380	5,990	-0,179	-0,124	-0,031
5,25E-03	1,40E-02	4,87E-03	-2,603	-1,380	4,244	2,637	0,013	0,703
1,25E-13	5,71E-12	3,23E-09	-2,588	-1,372	6,752	1,765	1,695	1,865
4,51E-11	7,21E-10	1,29E-06	-2,565	-1,359	6,841	1,940	1,881	1,934
6,26E-04	1,98E-03	3,11E-03	-2,558	-1,355	12,639	0,614	0,807	0,672
7,22E-13	2,36E-11	4,10E-03	-2,539	-1,344	7,258	2,460	2,874	2,862
2,21E-05	9,01E-05	1,57E-05	-2,537	-1,343	8,023	0,425	0,450	0,437
7,63E-13	2,48E-11	2,95E-04	-2,535	-1,342	6,822	-0,986	-1,383	-1,750
2,11E-05	8,63E-05	3,01E-02	-2,530	-1,339	2,800	1,507	1,277	1,378
5,90E-07	3,23E-06	4,96E-04	-2,516	-1,331	11,861	-0,781	-0,304	-1,112
9,24E-05	3,39E-04	1,03E-02	-2,509	-1,327	2,280	1,311	1,359	1,694
6,05E-08	4,08E-07	2,50E-02	-2,502	-1,323	2,344	2,212	0,770	2,098

1,71E-07	1,05E-06	1,98E-02	-2,495	-1,319	2,981	1,565	1,783	1,994
2,42E-12	6,26E-11	6,64E-04	-2,491	-1,317	4,078	2,278	2,778	2,546
7,70E-05	2,86E-04	5,09E-03	-2,490	-1,316	2,294	1,905	3,259	2,080
1,20E-02	2,98E-02	1,30E-02	-2,486	-1,314	3,236	0,185	0,859	0,993
4,08E-08	2,85E-07	4,19E-05	-2,481	-1,311	8,078	1,598	1,172	1,544
6,45E-04	2,04E-03	1,13E-02	-2,474	-1,307	4,564	0,251	0,251	0,184
2,81E-08	2,04E-07	1,52E-04	-2,471	-1,305	7,445	1,990	1,881	1,646
2,10E-10	2,71E-09	4,20E-03	-2,467	-1,303	3,774	2,237	2,039	1,898
5,12E-05	1,96E-04	8,75E-03	-2,461	-1,299	3,021	1,359	1,517	1,292
1,52E-03	4,48E-03	2,86E-02	-2,455	-1,296	3,082	0,950	0,598	0,680
2,09E-04	7,20E-04	1,11E-03	-2,454	-1,295	5,591	0,796	0,875	0,860
4,76E-05	1,83E-04	4,05E-02	-2,454	-1,295	4,163	-1,217	-0,838	0,012
4,77E-13	1,68E-11	2,27E-07	-2,450	-1,293	8,661	2,008	2,004	1,968
5,60E-07	3,08E-06	2,61E-02	-2,432	-1,282	3,212	2,315	1,886	2,704
1,70E-03	4,98E-03	8,74E-03	-2,427	-1,279	3,752	1,198	1,354	1,291
3,62E-07	2,08E-06	4,73E-04	-2,422	-1,276	13,561	-0,858	-0,505	-1,329
8,40E-03	2,15E-02	1,51E-02	-2,412	-1,270	3,330	0,642	0,732	1,543
1,74E-11	3,21E-10	9,81E-09	-2,408	-1,268	8,602	1,716	1,646	1,595
6,78E-05	2,54E-04	2,98E-03	-2,402	-1,264	5,009	0,217	0,028	0,144
7,75E-11	1,15E-09	1,95E-02	-2,398	-1,262	6,307	3,594	3,528	3,676
3,00E-11	5,09E-10	3,58E-07	-2,385	-1,254	8,004	2,010	2,092	1,868
1,48E-03	4,37E-03	6,69E-03	-2,382	-1,252	4,473	0,120	0,401	0,258
8,44E-08	5,53E-07	1,17E-02	-2,378	-1,250	8,036	-1,374	-0,826	-1,867
1,62E-03	4,75E-03	7,26E-03	-2,375	-1,248	3,243	1,127	0,607	1,211
1,00E-06	5,25E-06	6,07E-04	-2,359	-1,238	13,481	-0,803	-0,297	-1,240
1,31E-09	1,33E-08	2,71E-05	-2,355	-1,236	8,030	1,662	1,356	1,531
5,07E-14	2,86E-12	9,74E-04	-2,331	-1,221	6,225	3,073	2,927	3,129
6,58E-03	1,72E-02	5,32E-03	-2,326	-1,218	4,164	0,740	-0,006	0,604
2,85E-04	9,57E-04	4,81E-02	-2,325	-1,217	4,332	1,309	1,184	1,172
2,94E-09	2,72E-08	1,64E-08	-2,320	-1,214	6,650	0,350	0,589	0,469
5,26E-14	2,94E-12	3,39E-10	-2,315	-1,211	10,697	-0,311	-0,422	-0,208
1,12E-05	4,82E-05	1,35E-04	-2,313	-1,210	4,142	0,712	1,019	1,221
2,59E-10	3,24E-09	1,39E-03	-2,313	-1,210	3,174	2,015	1,959	2,153
3,74E-13	1,37E-11	1,96E-04	-2,312	-1,209	5,873	3,304	3,352	3,406
7,21E-07	3,89E-06	1,84E-05	-2,309	-1,207	7,747	1,187	1,196	1,217
1,24E-03	3,73E-03	1,20E-02	-2,301	-1,202	3,883	1,597	1,134	0,933
6,71E-03	1,75E-02	1,81E-02	-2,297	-1,200	3,677	1,315	1,020	0,793
7,76E-09	6,45E-08	4,30E-04	-2,294	-1,198	7,973	1,653	1,627	1,676
4,54E-09	4,00E-08	1,56E-05	-2,291	-1,196	6,939	1,490	1,672	1,490
1,49E-12	4,30E-11	4,99E-05	-2,282	-1,190	6,474	-0,886	-0,644	-0,995
6,78E-09	5,71E-08	1,04E-02	-2,277	-1,187	5,932	-1,532	-0,956	-0,327
3,87E-05	1,51E-04	3,65E-03	-2,274	-1,185	2,508	1,310	1,466	1,329
2,50E-11	4,36E-10	3,49E-06	-2,272	-1,184	5,678	1,541	1,288	1,501
1,36E-07	8,50E-07	5,97E-06	-2,270	-1,183	8,569	1,176	1,278	1,327
4,53E-07	2,55E-06	3,75E-04	-2,267	-1,181	5,559	1,661	1,255	1,577
1,08E-06	5,63E-06	3,59E-06	-2,266	-1,180	11,910	0,552	0,340	0,101
7,74E-04	2,41E-03	2,28E-03	-2,264	-1,179	3,450	0,549	-0,098	0,295
3,44E-10	4,15E-09	3,58E-08	-2,255	-1,173	9,314	-0,025	-0,056	-0,005
3,17E-03	8,84E-03	1,15E-02	-2,255	-1,173	2,895	1,404	1,451	1,167
4,96E-08	3,40E-07	9,20E-04	-2,244	-1,166	5,838	-1,038	-0,719	-1,214
2,03E-07	1,23E-06	9,51E-04	-2,236	-1,161	13,117	-1,041	-0,744	-1,570
6,38E-07	3,47E-06	4,05E-02	-2,231	-1,158	5,298	-0,796	-0,868	-0,261
1,72E-05	7,14E-05	1,12E-05	-2,224	-1,153	8,012	0,523	0,394	0,374
1,62E-08	1,24E-07	7,51E-06	-2,221	-1,151	8,638	-0,098	-0,200	-0,134
3,57E-07	2,05E-06	1,05E-02	-2,211	-1,145	4,749	1,377	1,335	1,463
3,72E-14	2,24E-12	2,89E-03	-2,207	-1,142	6,017	3,157	3,253	3,267

8,16E-10	8,82E-09	3,13E-06	-2,205	-1,141	7,580	1,621	1,916	1,896
7,30E-04	2,29E-03	1,14E-02	-2,201	-1,138	3,889	0,844	1,182	1,087
1,65E-10	2,20E-09	2,21E-04	-2,193	-1,133	6,178	1,723	1,862	1,776
4,57E-07	2,57E-06	9,33E-04	-2,190	-1,131	13,821	-0,936	-0,543	-1,376
3,02E-06	1,45E-05	1,43E-03	-2,187	-1,129	14,182	-0,778	-0,344	-1,147
2,48E-06	1,21E-05	2,79E-03	-2,187	-1,129	1,970	*1,643*	*1,620*	1,985
1,27E-06	6,53E-06	8,28E-03	-2,184	-1,127	7,976	-0,932	-0,761	-1,785
9,77E-07	5,14E-06	1,60E-06	-2,181	-1,125	11,679	0,293	0,110	0,221
5,58E-04	1,78E-03	8,12E-03	-2,175	-1,121	3,620	1,315	1,245	1,081
1,91E-03	5,54E-03	6,28E-03	-2,166	-1,115	10,265	0,644	0,704	0,710
4,40E-06	2,04E-05	5,25E-04	-2,164	-1,114	14,013	-0,462	-0,028	-0,713
1,19E-06	6,16E-06	7,20E-04	-2,160	-1,111	13,810	-0,699	-0,345	-1,094
7,89E-06	3,49E-05	1,39E-02	-2,151	-1,105	8,294	0,514	0,383	0,563
5,71E-04	1,82E-03	1,04E-02	-2,147	-1,102	4,346	1,108	0,576	1,669
1,49E-06	7,56E-06	2,91E-03	-2,135	-1,094	6,039	1,133	0,886	1,156
3,71E-06	1,75E-05	1,02E-02	-2,132	-1,092	7,953	-0,632	-0,217	-1,098
6,78E-05	2,54E-04	4,64E-03	-2,129	-1,090	5,044	1,347	0,972	1,548
4,99E-08	3,42E-07	2,86E-02	-2,127	-1,089	3,368	1,920	1,666	1,867
6,58E-08	4,40E-07	3,69E-03	-2,126	-1,088	7,861	-1,161	-0,609	-1,374
1,24E-05	5,28E-05	4,26E-03	-2,124	-1,087	13,431	-0,538	-0,328	-0,894
8,47E-06	3,73E-05	1,15E-04	-2,124	-1,087	6,700	0,271	-0,185	0,061
1,78E-06	8,88E-06	8,71E-03	-2,124	-1,087	6,953	-0,045	-0,047	0,332
1,14E-04	4,11E-04	1,69E-02	-2,121	-1,085	4,579	0,296	-0,646	0,038
3,42E-03	9,47E-03	2,98E-02	-2,116	-1,081	4,099	0,305	0,121	-0,233
2,53E-06	1,23E-05	3,35E-03	-2,114	-1,080	2,676	1,683	2,154	2,320
3,66E-08	2,58E-07	5,54E-03	-2,101	-1,071	2,475	1,827	1,644	1,931
6,67E-05	2,50E-04	2,46E-03	-2,099	-1,070	5,328	0,022	-0,058	-0,222
1,11E-04	4,00E-04	3,55E-02	-2,085	-1,060	2,852	1,538	1,663	1,100
1,27E-11	2,45E-10	5,92E-05	-2,083	-1,059	8,404	1,970	1,911	2,172
2,41E-04	8,20E-04	5,87E-04	-2,081	-1,057	4,299	0,230	0,783	0,421
1,54E-10	2,07E-09	1,01E-02	-2,076	-1,054	3,788	2,658	2,723	2,851
9,46E-04	2,90E-03	7,98E-03	-2,069	-1,049	4,605	-0,527	0,005	1,407
9,81E-11	1,41E-09	2,13E-04	-2,069	-1,049	7,215	1,910	1,786	1,815
9,91E-07	5,21E-06	1,26E-03	-2,066	-1,047	5,088	1,182	1,265	1,265
1,03E-05	4,46E-05	1,12E-03	-2,065	-1,046	14,018	-0,440	-0,054	-0,704
3,52E-05	1,39E-04	7,74E-05	-2,061	-1,043	5,108	0,265	0,133	0,061
2,02E-04	6,97E-04	4,05E-02	-2,056	-1,040	4,401	1,366	0,829	1,156
4,11E-09	3,67E-08	1,44E-06	-2,052	-1,037	6,910	1,220	1,191	1,183
2,52E-06	1,22E-05	2,73E-03	-2,051	-1,036	5,799	0,899	1,202	1,346
4,32E-05	1,67E-04	1,71E-03	-2,049	-1,035	7,398	-0,143	0,333	-0,013
6,82E-09	5,74E-08	1,31E-03	-2,046	-1,033	6,199	1,641	1,685	1,570
2,68E-08	1,95E-07	8,55E-05	-2,043	-1,031	5,206	1,264	1,292	0,904
4,01E-10	4,74E-09	1,01E-03	-2,043	-1,031	6,840	1,810	1,767	1,707
6,29E-08	4,22E-07	2,52E-04	-2,038	-1,027	6,071	1,129	1,236	1,268
6,49E-09	5,49E-08	2,52E-02	-2,034	-1,024	3,588	1,568	1,264	1,589
8,43E-03	2,16E-02	1,52E-02	-2,031	-1,022	5,135	0,665	0,062	0,379
2,93E-08	2,11E-07	2,46E-06	-2,029	-1,021	8,675	0,925	1,098	1,052
3,65E-03	1,01E-02	3,06E-03	-2,028	-1,020	5,374	0,576	0,507	0,660
1,30E-10	1,79E-09	1,44E-03	-2,022	-1,016	4,533	2,113	1,656	1,565
9,89E-06	4,30E-05	3,04E-04	-2,022	-1,016	8,692	0,901	0,704	0,738
2,56E-04	8,67E-04	4,99E-02	-2,011	-1,008	4,785	3,421	0,725	1,109
8,05E-12	1,68E-10	8,40E-04	-2,008	-1,006	4,057	3,012	2,427	3,040
2,65E-07	1,56E-06	2,57E-02	-2,006	-1,004	2,796	1,657	1,290	2,094
4,79E-08	3,30E-07	3,20E-07	-2,006	-1,004	8,962	0,914	0,899	0,955
1,86E-04	6,47E-04	5,77E-04	-2,006	-1,004	4,939	0,964	0,619	0,642
2,41E-13	9,67E-12	1,57E-04	-2,000	-1,000	4,059	2,267	1,841	2,285

wt_non-stim_4	wt_non-stim_21	CD4creGARPfl-fl_non-stim_22	CD4creGARPfl-fl_non-stim_23	CD4creGARPfl-fl_non-stim_8	CD4creGARPfl-fl_non-stim_9	CD4creGARPfl-fl_non-stim_10	wt_stim_11	wt_stim_12	wt_stim_13	wt_stim_14
4,092	4,351	-6,339	-6,465	-6,413	-6,052	-7,275	4,042	3,853	4,000	3,538
0,844	0,308	-4,485	-5,691	0,022	-4,958	-6,331	0,064	-0,054	0,931	0,553
0,832	0,409	-3,932	-5,281	0,030	-4,312	-5,653	0,081	-0,155	0,926	0,518
1,784	1,972	-1,923	-1,935	-1,907	-1,892	-1,550	1,874	2,156	1,935	2,452
4,028	2,627	0,242	0,552	0,467	0,397	0,611	-0,253	-0,267	-0,260	-0,248
1,794	1,430	-1,335	-0,987	-1,259	-0,897	-1,426	1,137	1,210	1,141	1,156
1,225	2,752	-0,022	1,007	-0,526	-0,522	-0,479	0,644	-0,034	0,022	0,184
3,011	3,948	1,204	0,624	0,597	1,257	0,167	-1,022	-0,565	-0,603	-1,225
-0,591	0,139	-2,946	-1,366	-2,840	-2,591	-2,804	1,511	1,483	1,509	1,756
-1,535	0,174	-1,819	-0,968	-2,807	-2,803	-2,822	-0,117	0,318	0,641	0,228
1,718	1,894	-0,576	0,103	-0,351	-0,524	-0,529	0,077	0,066	-0,066	0,259
3,789	3,532	0,765	1,485	1,691	1,654	1,357	-1,443	-1,490	-1,256	-0,765
2,584	3,298	0,577	0,451	0,397	0,288	0,204	-0,779	-0,884	-0,899	-1,323
4,228	4,674	1,924	3,557	3,253	2,373	0,157	-1,263	-0,157	-0,762	-0,815
0,722	0,415	-1,614	-1,438	-1,950	-1,929	-1,937	0,951	1,348	1,198	1,481
2,577	2,158	-0,075	0,132	-0,033	0,019	-0,005	-0,036	-0,063	-0,036	-0,076
3,623	4,016	1,500	1,536	1,179	1,918	0,398	-1,188	-1,890	-0,398	-0,692
1,003	0,075	-0,758	-1,178	-1,122	-0,580	-1,487	0,127	0,518	0,438	0,277
1,882	2,041	-0,220	0,366	0,083	-0,040	-0,333	0,018	-0,018	-0,123	0,299
1,909	2,117	-0,341	0,339	-0,028	-0,043	-0,322	0,028	0,060	-0,168	0,273
1,816	2,740	-0,727	1,052	-0,088	0,576	-1,002	0,583	0,098	-0,292	-0,114
4,158	3,919	1,090	1,955	2,197	2,176	1,665	-1,142	-1,266	-1,090	-1,120
2,868	3,293	0,992	1,189	0,788	0,672	0,321	-0,896	-0,849	-0,777	-0,973
2,504	1,651	-0,489	0,507	1,474	1,365	0,472	-0,744	-0,734	-0,718	-0,734
1,410	0,775	-1,045	-1,256	-0,993	0,305	-1,149	-0,203	0,280	-1,122	-1,181
0,941	0,568	-1,077	-1,592	-0,932	-1,274	-1,279	0,822	0,977	0,720	0,917
3,595	1,086	-0,228	1,115	1,014	2,195	1,521	-0,858	-0,012	0,012	-1,517
-1,860	-1,345	-3,845	-3,525	-4,185	-3,625	-4,058	1,760	1,734	1,871	2,043
0,458	0,514	-2,118	-1,236	-1,773	-1,452	-0,953	0,673	0,516	0,889	1,105
0,548	-0,357	-1,040	-1,598	-2,029	-2,054	-0,109	1,561	1,544	1,508	1,247
3,839	3,536	0,953	1,829	2,189	1,732	1,087	-2,348	-2,363	-2,352	-2,368
0,711	0,527	-1,226	-0,502	-1,676	-0,815	-1,615	0,678	0,258	0,720	-0,130
1,983	1,307	-0,244	0,409	0,244	0,529	-0,908	-0,966	-0,933	-0,863	-0,941
2,273	2,430	1,087	1,015	0,410	0,183	-0,856	-0,858	-0,204	-0,624	-0,858
0,778	-0,024	-1,097	-1,091	-1,076	0,086	-1,067	-0,507	-0,353	-0,571	-0,930
0,035	0,022	-1,789	-5,049	-1,402	-0,364	-4,872	0,737	0,838	0,699	0,961
-0,567	0,380	-2,393	-1,879	-1,534	-2,852	-3,588	0,290	0,352	0,350	0,100
0,758	0,566	-1,237	-1,531	-0,964	-0,849	-1,626	0,613	1,102	0,386	0,383
-0,366	0,461	-1,186	-1,203	-2,191	-2,058	-2,284	0,427	0,849	1,301	0,919
1,768	1,539	-0,146	0,285	0,250	0,006	-0,639	-0,155	0,172	0,489	-0,006
-0,896	-1,720	-3,071	-2,692	-2,998	-1,330	-3,154	0,896	1,309	0,970	1,205
1,911	0,496	0,185	0,526	-0,057	1,201	-0,073	0,027	-0,050	-0,054	0,378
2,888	2,388	0,015	0,722	1,522	1,040	0,861	-0,491	-0,182	-0,360	-0,015
-0,496	0,513	-2,205	-1,799	-1,495	-2,793	-3,385	0,230	0,346	0,384	0,032
2,243	1,982	0,218	-0,536	1,059	0,284	1,557	-0,538	-0,530	-0,173	-0,537
1,519	0,191	-0,278	-0,442	0,779	0,502	-0,974	-0,103	-0,932	0,136	-1,097

2,709	2,027	0,467	1,109	0,790	0,662	0,153	-0,734	-1,448	-0,153	-1,587
1,969	0,206	-0,304	-0,415	-1,714	-1,693	-0,858	-0,214	-0,025	1,063	0,496
1,338	1,656	0,009	-0,009	0,622	-0,569	0,246	0,203	-0,293	-2,029	-0,135
-0,636	-0,817	-0,676	-1,630	-1,519	-1,952	-2,339	1,842	1,820	2,081	1,040
2,657	2,665	0,603	1,011	1,096	2,078	0,079	-1,854	-1,875	-0,102	-1,641
-0,994	0,095	-2,638	-2,283	-1,978	-3,006	-4,020	0,264	0,352	0,347	0,151
2,714	1,639	-0,003	0,403	0,661	0,014	-0,121	-0,135	-0,150	-0,063	-0,171
0,638	1,085	-0,853	-1,026	-1,213	-0,271	-0,849	1,860	1,492	1,660	1,455
2,891	3,051	2,330	1,666	1,158	1,213	0,977	-1,185	-1,150	-1,183	-1,200
1,444	0,698	-0,477	-0,057	0,057	-0,156	-0,820	0,266	0,102	0,216	-0,253
3,590	3,412	1,039	1,974	2,056	1,927	1,472	-1,039	-1,119	-1,140	-1,126
-0,656	-0,006	-2,216	-1,737	-1,820	-2,326	-2,559	0,752	0,654	0,787	0,478
0,893	1,736	-0,464	-0,637	-0,621	0,371	0,106	-0,051	-0,053	0,306	0,223
0,992	1,142	-1,422	-0,116	-0,016	-0,724	0,621	0,185	0,094	0,137	-0,552
1,480	2,807	0,186	1,055	1,057	-0,099	0,706	-2,359	-2,349	-2,357	-1,137
1,542	1,301	-0,571	0,016	-0,248	0,186	-0,472	0,162	-0,217	0,124	-0,016
1,812	1,307	-0,654	0,007	-0,007	0,135	-0,393	-0,468	0,140	0,018	-0,580
-0,268	-0,959	-1,642	-1,483	-0,957	-1,176	-1,467	0,364	0,118	0,294	0,215
0,927	1,149	-0,023	0,448	-0,290	-1,147	-1,238	0,734	0,854	0,821	-0,212
-0,585	0,021	-2,116	-1,727	-1,687	-2,450	-3,005	0,362	0,333	0,235	0,198
1,128	1,170	-0,254	0,053	-0,455	-0,401	-0,796	0,200	1,695	0,381	-0,053
1,901	1,764	-0,120	0,371	0,719	0,554	-0,110	-0,089	-0,105	-0,096	-0,095
1,367	1,382	0,140	0,425	-0,162	-0,337	-0,399	-0,734	-0,217	-0,355	-0,596
1,841	0,511	-0,307	0,766	-0,220	1,161	-0,991	0,060	0,154	0,565	-0,577
2,196	2,740	0,153	1,852	-0,220	0,673	0,636	-0,232	-0,230	-0,215	-0,229
3,700	3,028	1,066	1,909	2,393	2,117	1,554	-1,532	-1,463	-1,066	-1,644
3,580	2,648	0,032	1,486	2,179	1,999	1,471	-0,456	-0,261	-0,395	-0,032
1,125	0,449	-0,587	-0,375	-0,288	-0,333	-0,702	0,199	0,067	0,084	-0,067
-0,677	0,088	-2,135	-1,749	-1,767	-2,510	-3,149	0,360	0,355	0,364	0,165
-0,528	-0,491	-2,501	-1,985	-1,781	-1,793	-2,839	1,224	1,218	1,187	1,254
1,152	0,333	-0,430	-1,259	-0,502	-0,603	-1,564	-0,701	0,687	1,179	1,233
2,216	1,140	-0,987	0,103	0,556	0,584	0,191	-0,363	-0,348	-0,103	0,504
0,813	0,385	-1,187	-1,194	-1,770	0,299	-0,889	0,354	0,419	0,980	0,900
-0,546	0,151	-1,983	-1,583	-1,565	-2,210	-2,849	0,506	0,415	0,552	0,294
1,317	1,420	-0,508	0,103	0,246	-0,013	-1,009	0,013	-0,152	0,204	-0,255
2,590	1,540	-0,204	0,628	1,144	1,018	0,371	-0,291	-0,141	-0,257	0,141
2,418	2,117	1,440	1,526	1,436	1,503	1,255	-1,255	-1,816	-2,233	-2,350
2,714	2,021	-0,030	0,812	1,281	1,086	0,625	-0,388	-0,201	-0,195	0,030
3,558	2,960	1,336	1,918	1,893	2,542	-0,019	-0,419	-0,435	-0,423	0,019
1,716	0,886	-0,467	0,256	0,666	0,618	0,056	-0,056	-0,277	-0,298	0,176
-0,634	0,041	-2,053	-1,936	-1,756	-2,384	-3,090	0,562	0,611	0,536	0,382
1,456	1,229	0,370	-1,320	-1,324	1,146	-0,176	-1,306	-0,901	-1,313	-1,308
4,034	3,114	1,996	2,385	3,033	3,360	2,425	-2,670	-1,996	-2,622	-2,668
-0,606	-0,176	-0,743	-1,723	-1,532	-1,397	-1,278	1,470	1,541	1,616	0,641
0,025	0,050	-1,414	-0,949	-1,162	-1,546	-1,411	1,335	1,316	1,368	1,509
0,512	0,214	-0,888	-0,256	-1,088	-0,583	-0,868	0,332	0,477	0,213	0,837
1,475	1,938	0,085	0,393	0,460	0,519	0,053	-0,053	-0,342	-0,237	-0,409
1,981	1,751	0,186	0,681	0,686	0,576	0,231	-0,799	-0,880	-0,778	-0,822
1,201	0,578	-1,823	-0,823	0,039	0,088	-0,683	-0,272	-0,039	0,069	0,322
3,160	1,852	1,518	1,828	1,488	2,112	1,385	-1,652	-2,047	-1,817	-1,631
0,426	0,099	-0,928	-0,357	-0,917	-0,734	-1,024	-0,007	0,139	0,112	-0,345
-1,383	-1,741	-2,748	-2,725	-2,365	-2,128	-3,317	1,895	2,106	2,026	2,511
1,530	0,655	0,636	1,116	-0,039	-0,232	0,039	-0,712	-0,940	-0,928	-0,927
-0,397	0,193	-1,728	-1,314	-1,470	-2,101	-2,642	0,378	0,448	0,420	0,238
1,502	0,199	0,032	-0,032	0,964	0,365	-0,316	-0,510	0,141	-0,510	-0,511
2,055	2,121	1,530	1,658	0,506	0,725	0,775	-0,506	-0,522	-0,513	-0,518

2,070	1,081	0,464	-0,030	1,380	1,565	0,334	0,030	-0,945	-0,908	-0,966
2,670	1,826	1,229	1,409	1,143	1,576	0,910	-1,192	-2,076	-2,058	-0,910
1,764	2,230	-0,264	1,446	0,764	1,498	-0,250	-0,259	-0,268	-0,263	0,202
0,340	1,066	-1,100	0,167	-0,444	-0,455	-0,845	0,140	-0,230	0,629	-0,052
1,652	1,615	0,393	0,922	0,287	0,257	-0,195	-0,229	-0,538	-0,229	-0,520
-0,470	-0,118	-1,124	-0,412	-1,202	-1,509	-1,108	1,041	0,416	0,760	-0,268
2,016	1,029	-0,071	0,577	0,928	0,860	0,552	0,046	-0,247	-0,046	-0,229
1,638	2,141	0,395	0,736	1,402	1,248	0,599	-1,569	-2,000	-0,907	-0,395
0,961	1,197	-0,096	-0,405	-0,008	0,124	0,939	-0,938	-0,985	0,008	-1,010
1,581	0,772	0,018	0,675	-0,719	-0,524	-0,705	0,196	-0,848	-0,018	-1,298
1,196	0,588	-0,472	-0,435	-0,151	0,422	-0,488	0,021	-0,021	0,143	0,204
-0,078	-0,258	-1,552	-1,903	-1,894	-1,005	-0,248	0,060	0,215	-0,012	0,333
2,084	1,881	0,130	0,738	0,711	0,989	0,586	-0,617	-0,287	-0,402	-0,130
2,435	1,962	0,931	1,033	1,603	1,803	-0,305	-0,364	-0,371	-0,934	-0,978
0,815	0,648	0,298	-0,169	-0,082	-0,068	-0,104	-0,827	-0,138	0,832	0,005
-0,567	0,033	-1,842	-1,640	-1,461	-2,382	-2,875	0,310	0,400	0,261	0,224
1,190	0,532	-1,149	-0,456	0,037	-1,315	-0,539	-1,029	-1,027	0,517	-0,037
1,832	1,560	0,013	0,473	0,409	0,378	-0,280	-0,013	-0,154	0,044	-0,391
-0,141	-0,606	-2,287	-1,237	-0,599	-1,666	-0,688	0,721	0,335	0,660	0,155
3,971	3,569	1,159	2,332	2,778	2,774	2,139	-2,454	-1,159	-2,247	-1,184
1,832	1,686	0,319	0,634	0,614	0,801	-0,030	-0,246	-0,244	-0,031	0,030
0,341	-0,805	-1,597	-0,994	-1,419	-0,599	-0,143	0,267	0,280	-0,137	0,245
-0,826	-0,131	-2,076	-1,906	-1,856	-2,189	-2,670	0,823	0,853	0,954	0,492
1,426	1,185	0,266	-0,352	-0,494	-0,063	0,015	-0,423	-1,076	0,593	-0,015
-0,462	0,170	-1,700	-1,499	-1,397	-2,223	-2,804	0,307	0,345	0,288	0,232
1,293	1,619	0,149	0,881	0,295	0,449	-0,149	-0,238	-0,396	-0,335	-0,561
3,104	2,491	1,234	1,853	2,364	2,415	1,743	-1,799	-1,853	-1,448	-1,262
0,595	-0,059	-0,258	-0,894	-0,743	-0,056	-0,623	-0,152	0,324	0,076	0,175
1,587	1,399	0,202	0,819	0,091	-0,092	0,013	-0,727	-0,503	-0,464	-0,013
0,464	0,270	-0,818	-0,750	-0,558	-0,740	-0,903	0,596	0,297	0,202	0,203
-0,221	-0,378	-1,264	-1,674	-1,692	-1,522	-1,320	0,515	0,476	0,641	0,666
1,004	0,970	-0,206	-0,038	0,133	-0,338	-1,073	0,136	0,431	-0,478	0,038
1,900	1,676	1,222	0,749	0,452	1,617	0,302	-1,286	-1,300	-1,292	-1,295
3,856	3,185	1,182	2,143	2,542	2,399	1,801	-1,518	-1,938	-1,894	-1,182
1,234	0,453	-0,255	-0,011	0,233	0,011	-0,481	-0,132	0,021	0,063	0,341
1,537	1,184	0,018	-0,018	-1,637	0,776	-0,115	-1,095	0,241	-0,924	0,392
0,921	0,572	-0,104	-0,282	-0,570	-0,256	-0,279	-1,238	0,149	-0,830	-0,472
1,903	1,536	-0,145	0,455	0,763	0,807	0,211	-0,460	-0,466	-0,265	-0,222
1,797	1,561	0,150	0,673	0,365	0,679	-0,053	-0,312	-0,704	-0,428	-0,730
-0,679	-0,785	-2,277	-1,614	-1,989	-1,819	-1,974	1,658	1,954	1,746	2,291
-1,332	-0,684	-2,144	-1,637	-1,423	-2,271	-3,229	1,902	1,855	1,751	1,111
1,317	1,665	0,144	1,328	-0,677	-0,537	0,411	0,127	-0,690	-0,120	-0,685
1,475	1,175	0,114	0,291	0,516	0,522	0,231	-0,114	-0,601	-0,317	-0,603
1,301	0,824	-0,540	0,201	0,094	0,313	-0,090	0,032	-0,181	-0,032	-0,285
1,473	1,073	-0,152	0,840	0,292	0,135	0,295	-0,214	-0,294	-0,104	-0,500
-0,019	-0,337	-1,630	-1,079	-0,897	-0,757	-1,101	0,135	0,163	0,200	-0,022
0,008	0,302	-0,819	-0,291	-0,885	-1,612	-1,619	0,253	0,785	0,530	0,651
-0,041	0,052	-0,851	-1,002	-1,376	-1,199	-1,714	0,470	0,520	0,516	0,496
0,936	0,666	-0,006	0,006	0,185	-0,404	-1,027	-1,018	-1,037	0,507	-1,035
-1,162	0,166	-2,146	-2,026	-2,204	-2,882	-2,764	1,035	0,499	1,208	0,932
-0,560	-0,032	-1,906	-1,813	-1,689	-2,438	-2,990	0,385	0,486	0,346	0,261
-1,084	-0,595	-1,069	-0,841	-1,954	-2,374	-2,984	1,596	1,710	1,747	1,818
0,417	0,056	-0,845	-0,367	-0,759	-0,745	-1,025	0,030	0,077	0,151	-0,388
-0,054	-0,047	-1,505	-0,707	-1,249	-1,145	-1,272	1,094	0,427	0,504	0,213
1,479	0,982	0,154	0,760	0,146	0,678	0,231	-0,972	-0,790	-0,297	-0,714
3,858	2,795	1,768	2,065	2,669	2,623	2,111	-2,013	-2,010	-1,768	-2,656

2,000	1,544	0,392	0,970	0,763	0,755	-0,048	-0,168	-0,306	-0,111	-0,142
0,901	0,964	-0,175	0,535	-1,006	0,418	-0,623	-1,066	-0,026	0,071	-1,242
1,540	1,999	0,865	1,286	0,643	0,451	0,253	-0,494	-0,487	-0,565	-0,253
-0,602	-0,003	-1,732	-1,582	-1,507	-2,325	-2,786	0,282	0,334	0,231	0,226
-0,430	0,104	-1,560	-1,347	-1,217	-2,115	-2,705	0,169	0,268	0,206	0,175
2,104	2,503	0,134	0,855	1,628	1,435	-0,159	-0,162	-0,157	0,185	-0,157
-0,340	-0,091	-1,734	-1,888	-1,421	-2,126	-2,707	0,497	0,622	0,437	0,195
0,350	0,011	-0,991	-0,587	-0,590	-0,905	-1,317	0,119	-0,019	0,097	-0,300
1,064	0,310	0,426	-0,050	0,268	-0,041	-0,949	-0,309	-0,389	-0,328	-0,564
0,329	-0,241	-0,639	-1,108	-0,053	-0,137	-0,470	0,386	0,185	0,053	0,214
-0,238	0,322	-1,352	-1,010	-0,984	-1,633	-2,189	0,265	0,300	0,184	0,157
-0,450	0,134	-1,561	-1,371	-1,239	-2,082	-2,576	0,270	0,285	0,231	0,228
0,651	0,574	-0,641	-0,126	-0,172	-0,542	-0,825	-1,309	-1,362	-1,436	-1,784
0,445	1,329	0,146	0,904	-0,126	-0,659	0,005	-0,722	-0,356	-0,005	0,087
1,295	1,559	0,355	0,791	0,049	0,062	-0,049	-0,554	-0,435	-0,597	-0,860
-0,138	0,000	-1,309	-1,453	-0,976	-1,299	-1,576	0,541	0,352	0,539	0,207
1,218	0,983	-0,053	0,852	0,596	0,128	-0,630	-0,388	-0,217	-0,153	-0,552
1,972	1,904	-0,025	0,816	1,519	1,319	0,571	-1,498	-1,500	-1,485	-0,007
-0,691	-0,069	-1,512	-1,780	-1,654	-2,136	-2,472	0,855	0,478	0,785	0,594
-0,226	0,313	-1,415	-0,921	-0,842	-1,562	-2,013	0,289	0,255	0,233	0,133
0,127	0,018	-1,026	-0,978	-0,937	-1,124	-2,344	0,491	0,650	0,694	0,075
-0,335	-0,257	-0,499	-1,174	-0,616	-1,583	-1,134	1,575	1,416	1,692	1,010
-1,223	-0,015	-1,100	-1,032	-0,778	-1,235	-2,123	0,638	0,512	0,168	0,655
-0,143	-0,014	-1,420	0,014	-1,095	-0,615	-1,893	0,295	0,405	-0,110	0,017
2,092	1,629	-0,111	1,012	1,460	1,025	-0,533	-0,789	-0,774	-0,766	-0,009
1,876	1,750	0,371	2,055	0,756	1,055	0,364	-0,432	-0,419	-0,385	-0,426
0,647	-0,638	-0,622	-1,300	-0,753	-1,724	-1,128	-0,066	-0,022	0,044	0,279
1,133	1,521	0,164	0,841	0,461	0,919	-0,931	-0,526	-0,908	-0,915	-0,164
2,233	2,018	0,315	1,006	1,058	0,960	0,614	-0,656	-0,986	-0,673	-0,315
0,839	0,193	-0,405	-0,364	-0,810	-1,367	-0,636	0,111	0,571	0,147	0,495
2,675	3,138	2,408	2,262	1,171	1,670	0,453	-0,453	-1,885	-1,887	-1,899
-0,618	0,323	-0,846	-2,340	-1,000	-1,189	-1,044	0,574	0,147	0,797	-0,156
1,848	1,610	0,228	0,766	0,963	1,318	0,240	-0,971	-0,472	-0,522	-0,228
1,214	1,082	0,590	0,049	0,031	0,168	0,182	-0,031	-0,432	-1,260	-0,690
-0,183	0,344	-1,230	-0,916	-0,900	-1,656	-2,209	0,237	0,264	0,256	0,237
0,284	0,003	-1,009	-0,398	-0,187	-0,910	-0,933	0,255	0,285	0,105	-0,003
1,015	1,318	-0,415	0,972	0,115	0,431	-0,024	-0,800	0,154	-0,767	0,024
1,224	1,143	-0,007	0,385	0,154	0,440	-0,232	0,007	-0,074	0,164	-0,367
1,554	0,893	0,216	0,437	0,166	0,079	-0,079	-1,288	-0,157	-0,343	-0,483
0,212	0,260	-0,844	-0,219	-0,823	-0,604	-0,874	0,366	0,199	0,577	0,261
1,423	1,618	0,397	0,584	0,892	0,969	0,246	-0,622	-0,383	-0,579	-0,648
0,882	1,228	0,667	-0,001	0,068	0,197	0,232	-0,221	-0,486	-0,045	0,001
2,013	1,071	0,735	1,012	0,944	0,642	0,479	-1,498	-0,626	-0,713	-0,743
1,011	0,991	0,007	0,101	0,358	0,232	0,031	-0,186	-0,152	-0,886	-0,286
1,842	0,943	0,369	0,544	0,999	0,860	0,515	-1,651	-1,077	-0,853	-1,636
0,521	-0,170	-0,895	-0,281	0,053	-0,643	-1,424	-0,052	0,017	0,188	0,415
1,229	0,650	-0,312	-0,013	0,294	0,306	0,032	-0,140	0,013	-0,146	0,020
0,574	0,501	-0,170	0,468	-0,530	-0,446	-0,937	0,005	0,062	-0,012	0,108
1,770	1,875	0,390	0,755	1,130	0,885	0,611	-1,140	-0,789	-0,504	-0,438
0,803	0,525	-0,874	-0,278	0,210	-0,186	-0,698	0,215	0,359	0,186	0,405
0,871	1,364	-0,241	0,578	0,008	0,244	0,101	-1,195	-0,454	-0,732	0,172
2,724	2,438	0,818	1,718	1,728	1,961	1,050	-1,859	-1,710	-1,278	-1,866
1,950	1,684	0,065	1,078	0,634	1,791	0,680	-0,822	-1,006	-0,309	-0,235
1,131	0,892	-0,497	0,098	-0,089	-0,045	-0,620	0,236	0,045	0,233	-0,099
0,529	1,085	-0,471	0,137	-0,161	-0,362	-1,188	0,089	0,435	0,435	0,006
2,173	2,040	0,999	1,173	1,215	1,182	0,779	-0,779	-1,876	-1,935	-1,960

wt_stim_15	CD4creGARPfl-fl_stim_24	CD4creGARPfl-fl_stim_25	CD4creGARPfl-fl_stim_18	CD4creGARPfl-fl_stim_19	CD4creGARPfl-fl_stim_20	Refseq	GeneID
3,707	-3,751	-3,538	-3,994	-4,058	-4,571	NM_001113379	434215
0,905	-4,340	-5,221	0,526	-4,469	-5,653		
0,906	-3,899	-4,805	0,453	-4,118	-5,167	NM_133362	170942
2,155	-1,889	-1,677	-1,870	-1,859	-1,862	NM_178165	229499
-0,242	-0,260	-0,247	-0,278	-0,285	-0,252	NM_175475	232174
0,897	-1,520	-1,685	-1,513	-1,760	-1,632	NM_001083318	27049
0,902	-0,535	-0,510	-0,523	-0,524	-0,505		
-0,167	-1,164	-1,755	-1,745	-1,739	-1,736	NM_026323	67701
1,030	0,179	-0,210	1,474	0,342	-0,072		
0,385	0,475	0,741	0,117	0,854	0,660	NM_001101431	70291
0,535	-1,172	-1,344	-2,213	-1,429	-1,497	NM_173786	223672
-2,086	-4,279	-4,279	-4,269	-4,262	-4,259	NM_001162938	100033459
-0,204	-2,658	-2,595	-1,815	-2,385	-2,608	NM_026323	67701
-3,004	-0,382	-2,445	-4,390	-1,631	-2,904	XM_357633	
0,918	0,000	-0,161	-1,117	0,400	-0,632		
-0,007	-0,090	0,005	0,018	-0,088	1,056	NM_007769	12945
-0,612	-1,889	-1,819	-1,829	-1,891	-1,838	NM_021400	96875
0,780	-1,474	-1,462	0,606	-1,456	-1,442	NM_172525	214137
0,779	-0,920	-0,910	-1,260	-1,251	-1,219	NM_173786	223672
0,697	-0,876	-1,020	-1,429	-1,477	-1,390	NM_001168660	71898
-0,994	-1,019	0,088	-1,007	-1,017	-1,016		
-1,843	-6,770	-5,372	-7,106	-6,505	-6,224	NM_001162938	100033459
-0,321	-2,845	-2,317	-2,153	-2,197	-2,250	NM_001033460	381738
-0,727	-0,746	-0,737	-0,735	-0,162	0,162		
-0,036	-0,268	0,246	0,342	-0,057	0,506	NM_013879	29867
0,423	-0,989	-0,423	-0,552	-0,437	-0,683		
-0,688	-1,500	-1,506	-1,506	-1,486	-1,501	NM_022420	64297
1,885	1,547	1,861	1,345	1,578	1,434	NM_134066	105349
0,940	-0,846	-0,228	-1,466	-0,360	-1,177	NM_175414	109246
1,231	-0,014	0,014	-1,015	-1,197	0,228		
-2,303	-0,953	-2,359	-2,338	-2,413	-2,381	NM_001033303	229665
0,610	-0,142	-0,334	-0,611	0,130	-0,735	NM_001162476	20897
-0,613	0,932	-0,251	0,483	-0,921	-0,764		
-0,832	-0,385	-0,825	-0,183	0,317	-0,836	NM_175206	74165
0,610	-0,462	0,082	1,787	0,836	0,463		
0,765	-0,960	-3,231	-0,355	0,371	-3,419	NM_001167883	99696
1,588	1,297	0,037	0,883	-0,037	0,264	NM_009946	12890
0,243	-1,675	-0,243	-0,755	-1,092	-0,982		
2,159	-0,452	0,179	0,743	0,515	0,171	NM_010180	14114
-0,190	-1,032	-1,163	-0,760	-0,751	-1,136	NM_008606	17385
1,417	1,567	1,169	0,933	1,073	0,912	NM_009189	20471
-0,044	-0,067	-0,036	-0,054	-0,059	-0,027	NM_053216	112418
-1,233	-0,923	-0,852	-0,750	-0,765	-0,290	NM_010517	16010
1,578	1,326	0,058	0,915	-0,032	0,232		
-0,529	-0,516	0,173	-0,527	-0,506	-0,520		
-0,982	-0,192	0,103	0,195	-1,158	-0,988	NM_144906	73094

-1,195	-1,263	-1,134	-0,779	-0,405	-0,639	NM_175206	74165
0,025	0,196	0,416	-0,416	-0,743	-0,691		
-0,281	-0,299	-0,546	0,048	-0,667	-0,327	NM_029464	75860
0,506	0,709	-0,097	-0,025	0,376	-0,029	NM_001177666	633640
-0,079	-1,649	-1,809	-1,789	-1,893	-1,879	NM_019707	12554
1,664	1,239	-0,022	0,984	0,022	0,212		
0,062	-0,181	-0,004	-0,017	0,003	-0,068	XM_889664	625123
1,648	-1,209	-0,196	-1,210	-0,283	-1,205	NM_178405	98660
-1,030	-1,175	-1,000	-1,005	-1,153	-0,977	NM_010259	14468
-0,921	-1,064	-0,938	0,503	-0,617	-1,268	NM_031183	83395
-1,533	-2,464	-1,876	-2,399	-2,112	-2,425	NM_001162938	100033459
1,881	0,869	0,216	0,921	0,006	0,147		
-0,672	-0,611	-0,436	-1,504	-0,457	0,051	NM_001014900	332934
0,016	-1,998	-0,674	-1,049	-2,358	-0,442	NM_001164518	210094
-2,354	-0,706	-2,353	-2,344	0,099	-2,340		
0,338	-2,884	-1,942	-1,830	-1,269	-1,790	NM_138665	192166
-0,523	-0,334	-0,418	2,400	-0,815	-0,516	NM_001039546	17920
0,087	0,428	0,218	-0,070	-0,088	-0,269	NM_152804	20620
-1,186	-0,241	-0,238	-1,197	-1,256	0,023		19085
1,088	0,379	0,051	0,456	0,008	-0,008		12890
0,219	-0,422	-0,783	-0,989	-0,855	-1,114	NM_008923	19085
-0,086	0,882	0,086	-0,121	-0,128	-0,104	NM_175176	209601
-0,703	0,441	-0,288	-0,140	0,220	0,338	NM_011347	20344
-0,974	-0,978	-0,524	-0,946	-0,060	-0,938	NM_028955	74466
1,455	-0,240	-0,237	-0,153	-0,243	-0,243		
-1,172	-1,914	-2,035	-2,360	-2,816	-2,004		216505
-0,467	-0,827	-0,749	-0,919	-0,680	-0,654	NM_019417	30794
-0,507	0,105	0,227	-0,313	-0,128	-0,337	NM_013749	27279
1,225	0,598	0,093	0,493	-0,013	0,013	NM_009946	12890
1,092	0,491	0,763	0,906	0,818	0,596	NM_001167996	68725
-0,569	-0,941	0,065	-0,065	0,297	-1,564	NM_172525	214137
-0,905	-1,531	-1,988	-0,488	-0,705	-0,728	NM_173744	72148
-0,266	-0,980	-0,729	-0,972	0,556	-0,948		
1,347	0,548	0,227	0,456	-0,001	0,001		
0,045	-1,428	-1,005	-0,654	-0,749	-1,201	NM_008606	17385
-0,320	-1,325	-1,406	-0,594	-0,826	-0,681	NM_173744	72148
-3,138	-1,511	-1,465	-3,212	-1,922	-1,766	NM_010423	15213
-0,718	-2,145	-1,591	-0,745	-0,815	-1,115	NM_011703	22354
-0,377	-0,413	-0,325	-0,412	-0,043	-0,452	NR_044988	621335
-0,695	-0,965	-0,385	-0,527	-0,061	-0,582	NM_026878	68939
1,539	0,667	0,217	1,003	-0,041	0,194		
-1,311	0,176	-1,190	0,390	-1,336	0,215	NM_008746	18213
-2,599	-2,672	-2,613	-2,635	-2,180	-2,637		791369
1,204	0,303	-0,016	-0,001	0,236	-0,150	XR_106831	100044171
0,989	0,223	-0,025	0,086	0,401	-0,033		
-0,070	-0,707	0,303	-0,307	-0,527	-0,013	NM_022030	64051
-0,455	-1,121	-1,311	-1,110	-1,323	-1,032	NM_172507	212531
-0,360	-0,186	-0,905	-1,031	-1,265	-0,695		
0,096	-1,666	-1,384	-0,232	-0,390	-0,690	NM_029723	76747
-1,385	-1,690	-2,201	-1,813	-2,672	-1,868		217151
0,604	0,278	0,007	-0,149	-0,284	-0,354	NM_207687	56226
1,456	1,180	1,301	1,523	1,466	0,986		
-0,933	0,529	-0,930	-0,774	0,274	-0,943	NM_001033343	240667
1,146	0,471	0,173	0,405	0,024	-0,024	XM_001474842	
-0,506	-0,505	0,205	-0,503	-0,231	-0,500	NM_178241	227288
-0,509	-0,535	-0,516	-0,521	-0,550	-0,528	XR_105427	

-0,878	-0,962	-0,886	-0,902	-0,951	-0,907		100038516
-0,999	-2,108	-2,035	-2,043	-2,098	-2,062		
-0,202	-0,256	-0,222	-0,238	1,296	-0,237		70858
-1,031	0,052	-0,417	-1,377	-0,610	0,334		
-0,971	-0,016	-0,050	-0,478	-0,274	0,016	NM_001081120	69627
0,725	-0,027	0,027	-1,337	0,186	0,085	NR_028311	100043335
-0,381	-0,700	-0,721	-0,300	-0,405	-0,341	NM_022019	63953
-2,004	-1,986	-2,001	-1,997	-1,987	-1,995		
-0,298	0,392	0,088	-0,928	-0,994	-0,932		
0,189	-1,287	-1,289	0,145	-1,272	-0,627		
-0,609	-1,078	-0,481	-0,515	-0,151	0,183	NM_001195437	235634
0,186	0,661	0,486	0,609	0,373	0,383		
-0,877	-1,360	-1,337	-1,313	-1,205	-1,394	NM_177292	320916
-1,360	-1,340	-1,341	-1,363	0,305	-1,349		240174
-0,945	0,314	-0,005	0,366	-0,118	-0,744		216760
1,002	0,291	0,034	0,392	-0,001	0,001		
-1,229	-0,306	0,311	2,169	-0,448	0,224	NM_172507	212531
-0,034	-0,677	-0,644	-0,618	-0,578	-0,588	NM_001033405	328833
0,649	-0,031	-0,096	0,091	0,868	-0,028		54651
-3,313	-3,400	-2,329	-2,622	-1,760	-3,477	XR_035179	330578
-0,074	-1,293	-0,995	-0,513	-0,814	-1,028		
0,522	-0,087	0,087	-0,132	0,247	-0,150		
1,998	0,850	0,306	0,980	0,131	0,328		
-0,386	0,631	0,350	-0,903	-0,086	-0,871		
0,989	0,354	0,095	0,424	0,013	-0,013		
0,260	-1,045	-0,858	-0,924	-0,957	-1,335	NM_175535	244867
-1,234	-1,914	-2,275	-2,057	-1,940	-2,479	NM_009162	20394
0,420	-0,639	-0,434	0,325	0,006	0,289	NM_175308	100465
-1,701	-0,204	0,361	-2,216	-0,194	-0,775	NM_028711	74011
0,671	-0,310	-0,549	-0,480	-0,202	-0,290	NM_001033405	328833
0,590	0,208	0,217	0,381	0,362	0,288	NM_012010	26905
0,478	-0,475	-0,931	-0,964	-0,538	-0,265	NM_172507	212531
-1,284	-1,308	-1,295	-1,312	-1,334	-0,302	NM_001012322	319229
-1,285	-1,854	-1,207	-1,721	-1,757	-2,268	XR_035179	330578
-0,451	-0,379	-0,570	-0,454	-0,424	-0,463	NM_016801	20907
-0,445	-0,060	0,119	-1,001	-1,325	-0,816		
0,537	0,104	-0,138	0,188	-1,731	0,237	NM_007425	11596
0,145	-2,216	-1,809	-0,772	-1,372	-1,015	NM_011374	20449
0,053	-0,501	-0,500	-0,169	-0,996	-0,575	NM_172443	207592
1,340	0,644	0,857	1,295	1,449	0,856		74750
2,397	0,762	0,568	0,876	1,142	0,327	NM_007992	14115
-0,685	0,120	-0,685	-0,526	-0,681	-0,697		
-0,854	-1,313	-1,113	-1,069	-0,774	-0,934	NM_178638	81907
0,351	-0,267	-0,563	-0,428	-0,601	-0,214	NM_178892	99929
-0,180	0,104	-0,436	-0,307	-1,004	-1,052	NM_025451	66259
0,019	0,071	0,033	-0,077	0,038	-0,042	NM_176933	319520
-0,598	-0,617	-0,228	-0,008	0,237	0,185		
0,365	0,043	0,053	-0,011	0,046	0,005	NM_022980	53902
0,847	-0,673	-0,638	-0,178	-0,511	0,460		
2,168	0,443	0,712	1,196	0,393	-0,166	NR_045506	71056
1,193	0,389	0,219	0,584	0,069	0,032		
0,261	0,555	0,822	0,636	1,370	1,011	NM_173419	239133
0,495	0,195	-0,030	-0,083	-0,195	-0,376	NM_207687	56226
0,499	0,182	0,047	0,181	0,266	0,062	NM_008967	19222
-0,146	-0,383	-0,912	-0,730	-2,071	-1,108	NM_009926	12815
-1,939	-2,159	-1,933	-2,449	-2,802	-1,836	NM_001080934	217316

0,048	-0,389	-0,503	-0,161	-0,269	-0,455	XM_003086262	
-0,883	-0,800	0,026	-0,221	-0,749	0,248	NM_029139	668110
-1,299	-1,373	-1,192	-1,018	-1,210	-1,456		
1,054	0,279	0,066	0,368	0,013	0,003	NM_011444	20678
0,975	0,266	0,024	0,359	0,018	-0,018		
-0,152	-0,142	-0,151	-0,151	-0,134	-0,156	NM_027001	69239
1,565	0,457	0,099	0,697	0,091	0,202		
-0,082	0,201	0,031	-0,075	0,287	-0,011	NM_001008700	16190
-0,659	-0,186	0,041	0,206	-1,188	0,193	NM_027909	71764
0,716	-0,466	-0,766	0,227	-0,323	-0,492	NR_001460	19782
0,948	0,381	0,049	0,351	0,028	-0,052		
1,000	0,316	0,031	0,349	0,022	-0,022		
-0,101	1,303	1,259	0,101	0,379	1,048	NM_010371	14940
0,044	-0,392	-0,333	-0,181	-0,235	-0,225		
0,082	-0,066	-0,577	-0,334	-1,203	-0,950	NM_001142732	101100
1,582	0,475	0,069	0,539	0,000	0,045		
-0,466	0,424	-0,092	-0,878	0,053	-0,291	NM_172631	52662
0,007	-1,484	-1,484	-1,506	-1,480	-1,489		18197
1,743	0,425	0,482	0,840	0,410	0,069		
0,994	0,563	0,103	0,407	-0,047	0,047		
0,132	-0,162	-0,018	-0,243	0,231	-0,278	NM_010287	14608
0,357	0,490	0,227	0,045	0,456	-0,078	NM_001039244	633640
0,425	0,151	0,063	0,015	-0,093	-0,575		
0,217	-0,414	0,869	0,129	-0,055	1,115		
-0,783	0,074	-0,762	-0,709	0,009	-0,754		
-0,364	-0,432	-0,386	-0,404	-0,429	-0,403		
0,787	0,426	0,027	0,625	0,279	0,472	NM_001033170	73813
-0,906	-0,920	-0,898	-0,916	-0,675	0,831		
-1,041	-2,350	-2,105	-1,479	-1,386	-1,398	NM_145838	241230
0,015	-1,063	-0,015	-0,034	-0,954	-0,343		
-1,890	-1,886	-1,880	-1,897	-1,878	-1,869	NM_010259	14468
1,315	0,217	-0,078	0,166	0,068	-0,005		
-0,956	-1,479	-1,644	-1,405	-1,296	-1,112	NM_007409	11522
-0,471	-1,023	-0,229	-0,079	-0,819	-0,101		
0,996	0,383	-0,026	0,330	0,026	0,076	NM_177354	238328
0,093	-0,184	0,115	-0,117	-0,156	-0,257		
-1,645	-0,185	-0,061	-0,903	-0,319	-0,169	NM_080595	140703
-0,289	-0,290	-0,284	-0,296	-0,530	-0,629	NM_175751	269023
-1,034	0,149	-0,308	-0,890	-0,961	-0,576	NM_001081069	50782
1,197	-0,306	0,013	0,063	-0,338	-0,160	NM_007561	12168
-1,060	-1,610	-0,856	-0,784	-0,246	-0,510	NM_183024	242570
-0,414	-0,468	-0,768	-0,550	-0,178	-0,710		
-0,982	-0,479	-0,638	-1,124	-0,899	-0,792	NM_144926	233878
-0,007	-0,651	-1,475	-0,981	-0,477	-0,590	NM_009800	12348
-1,638	-1,633	-0,578	-1,068	-1,270	-0,369		
0,911	-0,317	-0,277	0,348	-0,610	-0,017		
-0,088	-0,224	-0,378	-0,173	-0,418	-0,241	NM_011578	21814
-0,416	-0,005	0,388	-0,407	-0,434	-0,225	NM_145420	216080
-0,390	-1,220	-1,453	-2,029	-2,030	-2,575		
-0,485	-0,849	-0,850	-1,020	-0,288	-0,857	NM_001197321	108655
-0,008	-2,509	-1,274	-1,087	-1,007	-0,290		
-2,070	-0,818	-1,557	-1,899	-1,252	-2,088		
-1,004	-0,450	-0,752	-0,624	-0,994	-0,065	NM_001012322	319229
0,144	-0,354	-0,208	-0,368	-0,275	-0,669	NM_016851	54139
-0,467	-0,404	-0,006	-0,751	-0,504	-0,756	NR_027784	104103
-1,665	-1,930	-1,665	-1,656	-1,911	-1,673		

SeqID	ProbeID	GeneName	Description
2271329	A_55_P2031466	Lrrc32	Mus musculus leucine rich repeat containing 3
2307570	A_55_P2181222	E430029J22Rik	Mus musculus pyrin domain containing 3 (Pyd
2271288	A_55_P1985422	LOC100044227	Q811B0_MOUSE (Q811B0) Erythroid differen
2292890	A_55_P2034481	Erdr1	Mus musculus erythroid differentiation regulat
2290610	A_51_P349213	Fcrl1	Mus musculus Fc receptor-like 1 (Fcrl1), transc
2295189	A_52_P227267	Atp1a2	Mus musculus ATPase, Na+/K+ transporting,
2286413	A_66_P128761	E430029J22Rik	Mus musculus pyrin domain containing 3 (Pyd
2264815	A_55_P2062133	Etv3	Mus musculus ets variant gene 3 (Etv3), trans
2293489	A_51_P205779	Cd5l	Mus musculus CD5 antigen-like (Cd5l), mRNA
2280606	A_30_P0103165	A_30_P01031657	lincRNA:chr3:84609788-84618062 reverse st
2305426	A_52_P203316	Kcnf1	Mus musculus potassium voltage-gated chann
2290624	A_66_P103957	Bbs7	Mus musculus Bardet-Biedl syndrome 7 (huma
2307607	A_55_P2116536	Kcnf1	Mus musculus potassium voltage-gated chann
2311579	A_30_P0102377	A_30_P01023778	lincRNA:chr3:48896525-48994125 forward st
2261466	A_30_P0102202	A_30_P01022023	lincRNA:chr5:111602486-111603075 forward
2268697	A_66_P121480	Sardh	Mus musculus sarcosine dehydrogenase (Sar
2290566	A_30_P0102826	A_30_P01028268	lincRNA:chr3:37568488-37568964 forward st
2305499	A_30_P0102856	A_30_P01028564	lincRNA:chr10:98718526-98719169 reverse s
2309983	A_55_P2183914	Gm7120	Mus musculus predicted gene 7120 (Gm7120
2310432	A_55_P2015715	Rab4a	Mus musculus RAB4A, member RAS oncoger
2278852	A_51_P335702	Tmem114	Mus musculus transmembrane protein 114 (Tr
2273936	A_55_P2022971	Tspan9	Mus musculus tetraspanin 9 (Tspan9), mRNA
2276525	A_55_P1953341	Wfdc2	Mus musculus WAP four-disulfide core domair
2306301	A_55_P2148641	Rab4a	Mus musculus RAB4A, member RAS oncoger
2288123	A_55_P2038422	Adamts17	Mus musculus a disintegrin-like and metallope
2296517	A_55_P1985693	Fhdc1	Mus musculus FH2 domain containing 1 (Fhdc
2283507	A_55_P2269289	Dgkg	Mus musculus diacylglycerol kinase, gamma (
2284098	A_55_P2152956	Plekhm3	Mus musculus pleckstrin homology domain co
2265884	A_30_P0103276	A_30_P01032768	lincRNA:chr11:99048975-99062425 reverse s
2273566	A_30_P0102598	A_30_P01025989	lincRNA:chr3:84609788-84618062 reverse st
2291814	A_55_P2065651	Trav17	Mus musculus 16 days neonate thymus cDNA
2310774	A_55_P2107775	Apol9a	Mus musculus apolipoprotein L 9a (Apol9a), t
2305960	A_55_P2014100	Gm7120	Mus musculus predicted gene 7120 (Gm7120
2295472	A_30_P0103357	A_30_P01033579	lincRNA:chr1:138473514-138476054 reverse
2301160	A_30_P0103273	A_30_P01032731	lincRNA:chr16:64796849-64843249 reverse s
2303029	A_55_P2120866	LOC100044171	PREDICTED: Mus musculus hypothetical LOC
2306577	A_66_P110633	Apol9b	Mus musculus apolipoprotein L 9b (Apol9b), t
2305000	A_52_P124734	Cybrd1	Mus musculus cytochrome b reductase 1 (Cyb
2283521	A_52_P481279	Gm1060	Mus musculus coiled-coil domain containing 1
2264156	A_55_P2344244	4930556H04Rik	Mus musculus adult male testis cDNA, RIKEN
2286467	A_55_P2056192	NAP111293-1	Unknown
2259768	A_55_P2024634	Gm13546	predicted gene 13546 [Source:MGI Symbol;A
2281640	A_30_P0102038	A_30_P01020388	lincRNA:chrX:47890565-47915775 forward st
2286414	A_30_P0102552	A_30_P01025525	lincRNA:chr3:37659958-37665945 forward st
2290597	A_55_P2082786	Trib2	Mus musculus tribbles homolog 2 (Drosophila)
2279988	A_30_P0101903	A_30_P01019033	lincRNA:chr5:111602486-111603075 forward

2306135	A_30_P0102123	A_30_P01021230	lincRNA:chr3:37659958-37665945 forward st
2282750	A_66_P114705	Adamts6	Mus musculus a disintegrin-like and metallope
2269394	A_51_P424471	Cnih3	Mus musculus cornichon homolog 3 (Drosoph
2264259	A_55_P2001233	E430029J22Rik	Mus musculus pyrin domain containing 3 (Pyd
2280238	A_30_P0102690	A_30_P01026900	lincRNA:chr12:12916233-12919835 forward s
2300864	A_30_P0101804	A_30_P01018048	lincRNA:chr3:37659958-37665945 forward st
2296830	A_55_P2036627	E430029J22Rik	Mus musculus pyrin domain containing 3 (Pyd
2290163	A_55_P1967538	Hunk	Mus musculus hormonally upregulated Neu-as
2263575	A_55_P2043367	Apol9a	Mus musculus apolipoprotein L 9a (Apol9a), t
2265173	A_30_P0101803	A_30_P01018036	lincRNA:chr14:73746324-73749119 reverse s
2261431	A_30_P0101918	A_30_P01019182	lincRNA:chr1:138473514-138476054 reverse
2311496	A_52_P385594	Tal2	Mus musculus T-cell acute lymphocytic leuken
2297589	A_55_P1954393	Susd4	Mus musculus sushi domain containing 4 (Sus
2295316	A_30_P0102390	A_30_P01023909	lincRNA:chr15:92174226-92201201 forward s
2307188	A_51_P399845	Fgf2	Mus musculus fibroblast growth factor 2 (Fgf2
2305092	A_66_P121583	Gm7120	Mus musculus predicted gene 7120 (Gm7120
2299510	A_55_P2032815	Rnase13	Mus musculus ribonuclease, RNase A family,
2304970	A_30_P0103291	A_30_P01032917	lincRNA:chr12:15735906-15798506 reverse s
2310555	A_30_P0102844	A_30_P01028440	lincRNA:chr10:96096995-96097589 forward s
2296265	A_55_P2009225	Cd34	Mus musculus CD34 antigen (Cd34), transcrip
2284245	A_55_P1989738	4930426L09Rik	Mus musculus RIKEN cDNA 4930426L09 ger
2280302	A_51_P263290	Galnt9	Mus musculus UDP-N-acetyl-alpha-D-galactos
2297550	A_55_P2044582	Iglon5	Mus musculus IgLON family member 5 (Iglon5
2298259	A_55_P2012439	Tnfrsf19	Mus musculus tumor necrosis factor receptor s
2293972	A_30_P0101868	A_30_P01018688	lincRNA:chr5:111538420-111540142 forward
2266129	A_55_P2012824	Gm9426	Unknown
2298772	A_30_P0102188	A_30_P01021888	lincRNA:chr3:19179899-19216399 reverse st
2270548	A_52_P108850	St8sia1	Mus musculus ST8 alpha-N-acetyl-neuraminid
2275787	A_65_P08768	Fbln1	fibulin 1 [Source:MGI Symbol;Acc:MGI:95487]
2282633	A_55_P2017207	A430078G23Rik	Mus musculus RIKEN cDNA A430078G23 ge
2282004	A_30_P0101908	A_30_P01019082	lincRNA:chr1:33893475-33903900 forward st
2294545	A_30_P0102937	A_30_P01029370	lincRNA:chr11:95627874-95640824 forward s
2265157	A_52_P590535	Fbln2	Mus musculus fibulin 2 (Fbln2), transcript varia
2298126	A_55_P2074736	Prkar1b	Mus musculus protein kinase, cAMP depende
2277509	A_30_P0102232	A_30_P01022326	lincRNA:chr4:114631940-114632618 reverse
2260687	A_30_P0103064	A_30_P01030644	lincRNA:chrX:135880775-135891525 reverse
2258834	A_30_P0103194	A_30_P01031947	lincRNA:chr3:84609788-84618062 reverse st
2267983	A_55_P2105452	Foxp1	Mus musculus forkhead box P1 (Foxp1), trans
2292501	A_30_P0102555	A_30_P01025555	lincRNA:chr1:138473514-138476054 reverse
2279076	A_55_P2016792	Inpp4b	Mus musculus inositol polyphosphate-4-phosp
2262540	A_55_P2170034	Rgl1	Mus musculus ral guanine nucleotide dissocia
2300201	A_30_P0102902	A_30_P01029025	lincRNA:chr2:164739589-164745000 forward
2303699	A_51_P112223	Gsta4	Mus musculus glutathione S-transferase, alph
2299879	A_66_P135471	Gm4759	Mus musculus predicted gene 4759 (Gm4759
2295753	A_66_P105075	Mmp11	Mus musculus matrix metallopeptidase 11 (Mr
2264648	A_30_P0101906	A_30_P01019065	lincRNA:chr17:12965849-12968519 forward s
2262051	A_55_P2316041	BC050972	cDNA sequence BC050972 [Source:MGI Sym
2266603	A_30_P0101793	A_30_P01017933	lincRNA:chr12:15735906-15798506 reverse s
2303879	A_55_P1989698	Mmp11	Mus musculus matrix metallopeptidase 11 (Mr
2297691	A_55_P2162122	Gm8721	predicted gene 8721 [Source:MGI Symbol;Ac
2268867	A_55_P2184023	Dnahc7a	Mus musculus adult male hypothalamus cDNA
2297276	A_52_P491554	Zfp759	Mus musculus zinc finger protein 759 (Zfp759
2259461	A_55_P1993168	Ppargc1b	Mus musculus peroxisome proliferative activat

ANOVA p-value	Adjusted ANOVA p-value	Tukey p-value	Fold change	Log2 ratio	Median	wt_non-stim_1	wt_non-stim_2	wt_non-stim_3
1,26E-19	4,38E-16	2,28E-14	-230,241	-7,847	10,425	4,432	4,248	4,223
2,63E-16	6,03E-14	1,56E-11	-41,184	-5,364	9,146	4,039	3,987	4,053
4,28E-04	1,39E-03	7,87E-03	-32,492	-5,022	13,324	0,193	-0,022	1,055
4,09E-04	1,34E-03	7,27E-03	-24,847	-4,635	13,815	0,315	-0,030	1,024
1,48E-17	8,83E-15	2,29E-14	-16,201	-4,018	3,846	1,876	1,563	1,550
5,41E-08	3,68E-07	3,85E-07	-7,220	-2,852	3,016	1,533	0,196	0,764
8,36E-17	2,89E-14	6,59E-10	-7,091	-2,826	6,113	3,829	3,835	3,883
3,93E-16	7,93E-14	7,23E-14	-6,835	-2,773	9,485	1,584	1,704	1,810
5,21E-04	1,67E-03	6,11E-04	-6,285	-2,652	5,157	-0,770	-0,133	-2,379
5,27E-05	2,02E-04	2,98E-04	-5,633	-2,494	3,430	0,151	0,160	0,786
5,59E-06	2,55E-05	1,21E-04	-5,483	-2,455	2,039	0,459	-0,084	-0,077
7,46E-04	2,33E-03	7,80E-03	-5,267	-2,397	2,873	-0,917	-0,389	-0,333
6,81E-05	2,55E-04	9,80E-04	-5,260	-2,395	3,499	0,397	-0,536	-0,003
6,46E-03	1,69E-02	1,11E-02	-4,231	-2,081	2,988	-0,967	0,070	0,407
7,06E-08	4,69E-07	8,37E-05	-4,179	-2,063	4,519	0,808	0,669	0,589
2,01E-09	1,94E-08	7,18E-07	-3,874	-1,954	6,039	1,145	1,349	1,207
2,89E-04	9,71E-04	6,79E-03	-3,837	-1,940	2,488	-0,468	-0,175	0,942
8,84E-03	2,26E-02	1,78E-02	-3,660	-1,872	3,431	0,192	0,525	0,167
1,52E-06	7,70E-06	9,25E-03	-3,593	-1,845	5,676	0,025	0,155	0,232
1,95E-13	8,11E-12	1,11E-10	-3,399	-1,765	10,496	1,004	0,972	0,982
1,97E-02	4,67E-02	2,06E-02	-3,387	-1,760	3,538	-1,486	0,922	1,263
7,50E-08	4,96E-07	1,78E-05	-3,329	-1,735	4,084	0,228	0,487	0,607
9,68E-14	4,65E-12	2,34E-06	-3,272	-1,710	4,732	2,696	2,732	2,887
1,80E-10	2,37E-09	1,41E-08	-3,178	-1,668	7,905	0,885	0,667	0,788
1,16E-04	4,17E-04	5,07E-05	-3,057	-1,612	3,548	0,691	-0,028	0,652
1,11E-05	4,78E-05	1,12E-02	-3,029	-1,599	6,625	0,647	0,599	0,189
3,32E-03	9,22E-03	4,00E-03	-2,998	-1,584	4,698	0,331	-0,063	0,063
2,63E-11	4,56E-10	4,11E-10	-2,942	-1,557	6,368	0,420	0,520	0,615
1,30E-10	1,79E-09	3,30E-04	-2,878	-1,525	4,533	2,113	1,656	1,565
8,36E-06	3,68E-05	5,85E-04	-2,872	-1,522	4,015	0,340	0,343	-0,219
6,80E-03	1,78E-02	8,42E-03	-2,854	-1,513	2,762	-0,147	-0,242	0,367
1,84E-11	3,36E-10	2,17E-07	-2,840	-1,506	6,844	1,812	2,027	1,884
4,11E-05	1,60E-04	2,40E-03	-2,825	-1,498	4,882	-0,009	0,013	0,787
1,25E-06	6,44E-06	7,82E-06	-2,815	-1,493	5,508	0,467	0,336	0,351
1,43E-04	5,06E-04	3,81E-02	-2,778	-1,474	4,585	1,001	0,860	0,673
1,75E-08	1,33E-07	1,46E-04	-2,774	-1,472	6,496	0,013	0,001	0,310
1,19E-11	2,32E-10	1,03E-06	-2,734	-1,451	9,841	2,002	2,060	2,088
6,61E-05	2,48E-04	2,65E-04	-2,676	-1,420	4,578	0,557	0,019	0,434
1,74E-14	1,27E-12	6,71E-07	-2,641	-1,401	8,074	2,634	2,887	2,792
7,39E-07	3,97E-06	1,83E-05	-2,606	-1,382	5,567	0,408	0,661	0,486
1,46E-10	1,98E-09	1,76E-07	-2,594	-1,375	7,163	0,475	0,705	0,782
3,78E-07	2,16E-06	4,73E-05	-2,583	-1,369	4,748	0,311	0,390	0,774
3,51E-03	9,70E-03	4,43E-02	-2,579	-1,367	4,947	0,262	-0,087	0,087
1,39E-07	8,68E-07	2,73E-04	-2,565	-1,359	3,768	0,072	-0,483	
1,32E-08	1,04E-07	1,13E-02	-2,535	-1,342	5,900	1,398	1,452	1,347
5,11E-07	2,84E-06	9,53E-03	-2,519	-1,333	5,119	0,994	0,964	0,961

1,62E-08	1,24E-07	1,64E-02	-2,512	-1,329	5,013	0,072	-0,265	-0,566
5,33E-05	2,04E-04	2,23E-03	-2,464	-1,301	6,937	0,598	0,860	0,971
2,11E-18	2,27E-15	1,54E-07	-2,420	-1,275	4,010	-2,135	-2,153	-2,157
7,51E-16	1,24E-13	6,66E-05	-2,415	-1,272	9,641	3,474	3,498	3,579
2,73E-05	1,10E-04	5,82E-03	-2,392	-1,258	4,832	0,611	0,870	0,624
2,96E-12	7,36E-11	1,47E-07	-2,377	-1,249	5,990	-0,179	-0,124	-0,031
3,73E-16	7,64E-14	1,29E-05	-2,372	-1,246	7,821	2,841	3,189	2,974
3,86E-06	1,81E-05	8,89E-03	-2,362	-1,240	2,417	-0,374	-0,393	-0,387
1,19E-11	2,32E-10	1,91E-06	-2,357	-1,237	9,361	2,081	2,086	2,083
7,72E-09	6,42E-08	4,96E-02	-2,341	-1,227	5,322	2,054	2,381	1,921
4,26E-03	1,16E-02	1,55E-02	-2,336	-1,224	4,462	0,299	0,114	0,446
9,53E-08	6,17E-07	2,72E-02	-2,304	-1,204	3,064	-0,918	-0,931	-0,913
2,03E-09	1,96E-08	1,42E-04	-2,301	-1,202	7,896	1,018	0,963	1,113
7,89E-03	2,04E-02	3,99E-02	-2,289	-1,195	3,763	1,440	-0,151	0,366
3,36E-12	8,13E-11	3,93E-04	-2,283	-1,191	3,816	-1,069	-1,408	-1,702
1,78E-06	8,88E-06	5,72E-03	-2,282	-1,190	6,953	-0,045	-0,047	0,332
3,17E-03	8,83E-03	6,75E-03	-2,277	-1,187	3,296	-0,183	1,178	0,045
1,52E-09	1,52E-08	5,56E-03	-2,277	-1,187	6,275	1,079	1,215	1,440
1,55E-10	2,08E-09	2,94E-03	-2,250	-1,170	7,292	2,143	2,320	1,996
6,18E-03	1,63E-02	3,60E-02	-2,249	-1,169	4,304	0,282	0,863	0,467
6,10E-04	1,94E-03	1,52E-03	-2,247	-1,168	5,491	0,131	0,611	0,352
5,33E-08	3,63E-07	6,68E-05	-2,238	-1,162	6,730	0,632	1,016	0,559
3,55E-05	1,40E-04	2,02E-02	-2,208	-1,143	4,840	1,659	1,691	1,440
3,74E-07	2,14E-06	3,06E-02	-2,207	-1,142	4,073	0,022	-1,756	-0,750
5,38E-04	1,72E-03	3,67E-02	-2,181	-1,125	4,827	0,285	0,591	0,599
2,48E-03	7,04E-03	8,32E-03	-2,178	-1,123	4,541	-0,011	-0,068	-0,050
1,45E-06	7,37E-06	5,28E-04	-2,155	-1,108	4,421	0,102	0,817	0,339
7,76E-09	6,45E-08	8,14E-04	-2,154	-1,107	7,973	1,653	1,627	1,676
7,26E-08	4,81E-07	4,57E-04	-2,149	-1,104	5,917	0,006	-0,146	0,241
1,96E-11	3,54E-10	3,85E-03	-2,148	-1,103	5,806	1,898	1,968	1,865
2,13E-03	6,11E-03	3,70E-02	-2,142	-1,099	3,449	-0,045	0,149	-0,665
3,66E-07	2,10E-06	2,18E-03	-2,136	-1,095	4,320	0,898	0,484	0,450
6,78E-09	5,71E-08	2,03E-02	-2,132	-1,092	5,932	-1,532	-0,956	-0,327
5,19E-06	2,38E-05	4,86E-04	-2,105	-1,074	7,097	1,176	0,993	0,986
3,89E-03	1,06E-02	2,76E-02	-2,104	-1,073	3,045	-1,035	0,220	-0,089
3,24E-03	9,00E-03	1,65E-03	-2,101	-1,071	3,412	-0,852	-0,078	0,405
6,24E-04	1,98E-03	1,16E-02	-2,098	-1,069	3,560	0,114	-0,114	0,444
9,89E-06	4,30E-05	1,96E-03	-2,092	-1,065	8,692	0,901	0,704	0,738
4,21E-06	1,96E-05	2,85E-05	-2,092	-1,065	6,137	0,520	0,123	0,109
1,25E-03	3,75E-03	3,14E-03	-2,086	-1,061	5,411	-0,093	0,271	0,914
9,55E-07	5,03E-06	4,08E-04	-2,082	-1,058	5,040	-0,620	-0,838	-0,893
5,35E-11	8,35E-10	6,38E-03	-2,053	-1,038	4,807	1,905	1,735	1,546
4,04E-13	1,46E-11	1,47E-05	-2,043	-1,031	6,881	1,619	1,474	1,692
2,82E-11	4,85E-10	1,76E-02	-2,038	-1,027	5,744	1,665	1,170	1,323
8,84E-10	9,45E-09	1,32E-04	-2,036	-1,026	5,941	1,881	1,358	1,877
1,91E-03	5,54E-03	1,01E-02	-2,029	-1,021	4,386	0,573	0,026	0,556
2,79E-06	1,34E-05	1,65E-03	-2,027	-1,019	1,774	0,000	-0,001	-0,003
1,52E-10	2,05E-09	2,79E-03	-2,027	-1,019	6,835	1,318	1,471	1,368
2,47E-08	1,81E-07	1,35E-03	-2,025	-1,018	7,365	1,622	1,331	1,780
7,02E-03	1,83E-02	2,63E-02	-2,022	-1,016	4,381	0,062	-0,018	0,834
8,55E-04	2,64E-03	8,74E-03	-2,021	-1,015	3,690	0,489	0,045	0,891
1,15E-06	5,97E-06	1,09E-02	-2,017	-1,012	3,052	-1,055	-1,066	-1,053
6,65E-03	1,74E-02	7,60E-03	-2,010	-1,007	4,264	0,274	0,454	-0,040

wt_non-stim_4	wt_non-stim_21	CD4creGARPfl-fl_non-stim_22	CD4creGARPfl-fl_non-stim_23	CD4creGARPfl-fl_non-stim_8	CD4creGARPfl-fl_non-stim_9	CD4creGARPfl-fl_non-stim_10	wt_stim_11	wt_stim_12	wt_stim_13
4,092	4,351	-6,339	-6,465	-6,413	-6,052	-7,275	4,042	3,853	4,000
4,158	3,919	1,090	1,955	2,197	2,176	1,665	-1,142	-1,266	-1,090
0,844	0,308	-4,485	-5,691	0,022	-4,958	-6,331	0,064	-0,054	0,931
0,832	0,409	-3,932	-5,281	0,030	-4,312	-5,653	0,081	-0,155	0,926
1,784	1,972	-1,923	-1,935	-1,907	-1,892	-1,550	1,874	2,156	1,935
0,638	1,085	-0,853	-1,026	-1,213	-0,271	-0,849	1,860	1,492	1,660
3,789	3,532	0,765	1,485	1,691	1,654	1,357	-1,443	-1,490	-1,256
1,794	1,430	-1,335	-0,987	-1,259	-0,897	-1,426	1,137	1,210	1,141
0,029	1,399	-0,271	0,387	-0,029	0,661	2,570	0,520	0,884	0,835
-0,525	-1,348	-1,406	-1,388	-1,330	-1,277	0,068	1,266	1,314	1,603
0,478	0,111	-0,099	-0,085	-0,077	-0,069	-0,078	2,268	2,496	2,496
-0,936	-0,941	1,043	-0,963	0,333	1,457	-0,968	1,745	2,013	2,059
0,277	0,003	-0,942	-1,423	-1,378	-1,313	-1,362	0,748	0,194	1,607
-0,667	0,081	-0,680	-0,971	1,151	-0,964	-0,957	0,966	1,211	0,313
0,416	1,754	0,185	1,839	0,631	0,671	-0,079	-1,072	-0,335	0,079
1,542	1,301	-0,571	0,016	-0,248	0,186	-0,472	0,162	-0,217	0,124
-0,689	-0,163	-0,701	-0,090	0,539	-0,683	-0,687	1,452	1,748	2,563
0,452	-1,574	-1,071	-0,559	0,180	0,925	0,312	-0,167	-1,445	0,456
-0,636	-0,817	-0,676	-1,630	-1,519	-1,952	-2,339	1,842	1,820	2,081
0,932	0,749	0,241	-0,318	0,395	0,124	0,340	-0,023	-0,160	-0,042
0,924	-0,650	0,341	0,116	0,282	-0,477	-0,411	0,643	0,262	-0,116
0,458	0,514	-2,118	-1,236	-1,773	-1,452	-0,953	0,673	0,516	0,889
2,584	3,298	0,577	0,451	0,397	0,288	0,204	-0,779	-0,884	-0,899
0,604	0,601	-0,023	-0,652	0,238	-0,042	0,295	-0,084	0,031	-0,104
-0,462	-0,623	0,128	0,028	-0,660	-0,065	0,066	0,710	0,773	1,078
-0,068	0,089	2,834	-0,087	0,880	1,562	2,387	-0,169	0,068	0,078
-0,543	0,428	-0,690	-0,170	-1,043	0,160	-0,215	0,846	0,442	0,807
0,601	0,677	-0,493	-0,136	-0,347	-0,212	-0,056	0,308	0,344	0,261
1,770	1,875	0,390	0,755	1,130	0,885	0,611	-1,140	-0,789	-0,504
0,548	-0,357	-1,040	-1,598	-2,029	-2,054	-0,109	1,561	1,544	1,508
0,470	-0,438	1,058	0,055	-0,157	-0,943	-0,088	1,342	0,487	0,576
1,718	1,894	-0,576	0,103	-0,351	-0,524	-0,529	0,077	0,066	-0,066
0,002	-0,446	-0,597	-1,298	-0,489	-1,526	-0,740	1,495	1,966	2,065
0,249	0,013	-0,984	-0,406	-0,317	0,100	-0,965	-0,013	0,309	0,101
0,469	1,256	0,208	1,634	-0,082	0,727	-0,175	0,185	-0,697	0,010
-0,606	-0,176	-0,743	-1,723	-1,532	-1,397	-1,278	1,470	1,541	1,616
1,909	2,117	-0,341	0,339	-0,028	-0,043	-0,322	0,028	0,060	-0,168
0,541	0,753	0,009	0,363	-0,225	-0,344	-0,037	0,388	-0,009	0,719
2,868	3,293	0,992	1,189	0,788	0,672	0,321	-0,896	-0,849	-0,777
0,462	0,548	-0,204	0,328	-0,131	0,131	-0,307	0,342	-0,229	0,482
0,941	0,568	-1,077	-1,592	-0,932	-1,274	-1,279	0,822	0,977	0,720
0,758	0,566	-1,237	-1,531	-0,964	-0,849	-1,626	0,613	1,102	0,386
0,813	0,385	-1,187	-1,194	-1,770	0,299	-0,889	0,354	0,419	0,980
0,722	0,415	-1,614	-1,438	-1,950	-1,929	-1,937	0,951	1,348	1,198
1,710	1,221	0,579	0,860	0,836	0,853	0,320	-0,546	-0,888	-0,527
0,873	2,123	0,925	2,259	1,044	1,122	0,070	-0,365	-0,194	-0,070

-0,591	0,139	-2,946	-1,366	-2,840	-2,591	-2,804	1,511	1,483	1,509
0,762	0,615	-0,317	-0,155	0,092	0,192	-0,305	-0,092	-0,236	0,161
-2,136	-2,161	-2,140	-2,160	-2,135	-2,162	-2,162	3,493	3,551	3,542
3,590	3,412	1,039	1,974	2,056	1,927	1,472	-1,039	-1,119	-1,140
0,239	1,086	-0,065	0,710	0,480	-0,076	-0,377	-0,378	0,065	-0,381
0,025	0,050	-1,414	-0,949	-1,162	-1,546	-1,411	1,335	1,316	1,368
3,287	2,938	2,022	2,608	2,294	2,326	1,784	-1,916	-2,347	-1,784
0,320	-0,393	-0,435	-0,430	-0,388	-0,344	-0,375	2,290	1,740	2,182
1,882	2,041	-0,220	0,366	0,083	-0,040	-0,333	0,018	-0,018	-0,123
2,140	3,029	1,854	2,676	1,818	1,626	0,601	-0,718	-1,133	-0,601
0,558	-0,020	-0,443	0,065	-0,132	0,020	-1,244	0,296	0,108	0,446
-0,232	-0,920	-0,990	-0,952	-0,939	-0,912	-0,904	1,940	2,595	2,460
0,462	1,090	0,063	0,392	0,432	0,793	0,280	-0,931	-0,860	-0,704
0,846	0,235	0,409	-0,357	0,044	0,425	-0,044	-0,297	0,488	0,633
-1,354	-1,260	-1,758	-1,765	-1,785	-0,859	-1,741	1,982	2,258	2,255
-0,335	-0,257	-0,499	-1,174	-0,616	-1,583	-1,134	1,575	1,416	1,692
1,072	0,512	-0,045	-0,163	-1,364	0,069	0,288	0,677	0,646	1,034
1,553	1,155	0,689	1,155	0,810	1,231	0,587	-1,122	-0,685	-0,658
1,961	2,300	0,900	2,094	1,193	1,400	0,459	-0,629	-0,579	-0,608
-0,505	0,586	-0,206	0,287	0,250	0,320	-0,329	0,642	0,032	0,358
0,476	0,381	-0,520	-0,191	-0,377	0,353	-0,450	0,541	0,385	0,718
0,577	0,595	-0,195	0,172	-0,023	0,085	0,076	-0,299	0,023	-0,032
0,992	1,142	-1,422	-0,116	-0,016	-0,724	0,621	0,185	0,094	0,137
-1,448	-1,168	-2,091	-0,534	-2,051	-2,044	-2,050	1,386	2,135	1,926
0,446	1,487	0,387	1,510	-0,265	0,043	-0,590	0,011	-0,011	-0,022
0,724	0,021	0,242	0,084	0,113	-0,104	0,000	0,132	0,237	0,000
0,557	0,581	-0,037	0,292	0,408	0,088	-0,141	-0,248	-0,614	-0,033
1,903	1,536	-0,145	0,455	0,763	0,807	0,211	-0,460	-0,466	-0,265
-0,006	0,039	-0,813	-1,245	-0,895	-0,768	-1,143	1,325	1,050	0,843
1,974	1,667	0,945	1,538	1,642	1,396	1,130	-2,002	-1,885	-1,496
-1,091	-0,826	-0,749	0,186	-0,333	-1,409	-0,009	1,219	0,880	0,602
0,498	0,903	0,136	0,249	0,827	-0,026	0,197	-0,412	-0,855	0,026
-1,332	-0,684	-2,144	-1,637	-1,423	-2,271	-3,229	1,902	1,855	1,751
1,128	1,170	-0,254	0,053	-0,455	-0,401	-0,796	0,200	1,695	0,381
-1,066	-0,536	0,694	-1,105	-0,759	-0,749	-0,373	0,201	1,162	1,191
-0,038	0,441	-0,046	-0,378	-0,241	0,639	0,541	0,626	1,608	0,969
-0,623	0,177	-1,265	-0,464	-1,524	-1,453	-0,398	1,624	0,839	1,205
0,803	0,525	-0,874	-0,278	0,210	-0,186	-0,698	0,215	0,359	0,186
0,383	0,119	-0,638	-0,728	-0,084	0,072	-0,563	-0,053	0,053	0,166
0,009	0,384	-0,551	0,205	-0,009	0,068	-1,224	-0,023	0,150	0,196
-1,207	0,046	-0,244	-0,046	-0,822	-0,223	-0,820	2,335	1,194	1,381
1,850	1,504	1,039	1,196	1,035	1,210	0,291	-0,959	-0,492	-0,492
1,275	1,538	0,894	0,519	0,884	0,858	0,509	-0,509	-0,623	-0,561
1,183	1,487	0,855	1,295	1,851	1,272	1,104	-1,521	-2,361	-1,446
1,768	1,539	-0,146	0,285	0,250	0,006	-0,639	-0,155	0,172	0,489
0,392	1,080	-0,470	1,024	-0,262	-0,026	-0,682	0,523	0,311	0,681
0,000	-0,001	0,573	-0,003	-0,002	-0,006	-0,005	2,494	1,939	2,142
1,657	1,157	0,723	1,277	1,126	1,053	0,436	-0,743	-0,436	-0,482
1,317	1,420	-0,508	0,103	0,246	-0,013	-1,009	0,013	-0,152	0,204
0,018	0,398	-0,619	0,198	0,076	-0,543	-1,058	0,405	0,633	0,620
0,022	0,867	-1,117	-0,022	0,638	0,167	-0,575	-0,055	0,142	0,439
-1,063	-0,999	0,159	0,008	-0,738	-1,024	0,079	0,421	0,880	1,133
0,441	0,157	-0,372	0,019	-0,685	0,496	-0,175	0,435	0,176	0,748

wt_stim_14	wt_stim_15	CD4creGARPfl-fl_stim_24	CD4creGARPfl-fl_stim_25	CD4creGARPfl-fl_stim_18	CD4creGARPfl-fl_stim_19	CD4creGARPfl-fl_stim_20	Refseq
3,538	3,707	-3,751	-3,538	-3,994	-4,058	-4,571	NM_001113379
-1,120	-1,843	-6,770	-5,372	-7,106	-6,505	-6,224	NM_001162938
0,553	0,905	-4,340	-5,221	0,526	-4,469	-5,653	
0,518	0,906	-3,899	-4,805	0,453	-4,118	-5,167	NM_133362
2,452	2,155	-1,889	-1,677	-1,870	-1,859	-1,862	NM_178165
1,455	1,648	-1,209	-0,196	-1,210	-0,283	-1,205	NM_178405
-0,765	-2,086	-4,279	-4,279	-4,269	-4,262	-4,259	NM_001162938
1,156	0,897	-1,520	-1,685	-1,513	-1,760	-1,632	NM_001083318
1,311	1,600	-1,436	-1,558	-3,171	-2,815	-1,767	NM_009690
1,228	1,598	-0,040	-1,209	-1,200	0,040	-1,180	
2,195	2,374	0,069	-0,096	-0,081	2,201	-0,088	NM_201531
2,173	1,642	0,513	-0,935	-0,384	1,652	-0,923	NM_027810
1,092	2,063	0,139	0,367	-1,353	-1,370	-1,303	NM_201531
1,140	1,696	0,463	-0,941	-0,970	-0,070	-0,983	
-0,757	-0,148	-2,351	-2,391	-2,398	-2,479	-2,417	
-0,016	0,338	-2,884	-1,942	-1,830	-1,269	-1,790	NM_138665
2,030	2,458	0,090	-0,686	1,466	1,361	-0,671	
0,285	0,925	-1,603	-1,587	-1,578	-1,485	-1,593	
1,040	0,506	0,709	-0,097	-0,025	0,376	-0,029	NM_001177666
-0,057	0,023	-1,807	-1,638	-1,853	-1,650	-1,899	NM_009003
0,180	1,008	-1,584	-0,898	-1,499	-1,572	-0,164	NM_029070
1,105	0,940	-0,846	-0,228	-1,466	-0,360	-1,177	NM_175414
-1,323	-0,204	-2,658	-2,595	-1,815	-2,385	-2,608	NM_026323
0,023	0,329	-1,606	-1,644	-1,992	-1,534	-1,821	NM_009003
1,455	0,679	-1,580	-0,269	-1,559	-0,672	-0,838	NM_001033877
-0,375	-0,351	-1,045	-1,409	-1,915	-1,769	-2,003	NM_001205355
0,311	1,974	-0,316	-0,778	0,417	-0,984	-0,927	NM_138650
0,401	0,056	-1,143	-1,252	-1,520	-1,249	-0,958	NM_001039493
-0,438	-0,390	-1,220	-1,453	-2,029	-2,030	-2,575	
1,247	1,231	-0,014	0,014	-1,015	-1,197	0,228	
1,446	1,611	-0,171	-0,055	-0,940	0,450	-0,300	
0,259	0,535	-1,172	-1,344	-2,213	-1,429	-1,497	NM_173786
0,972	0,438	0,384	-0,002	-0,899	0,256	-0,149	NM_001177666
0,036	0,109	-1,393	-1,855	-1,237	-0,844	-1,498	
-0,010	-0,522	-0,437	-1,484	-1,980	-0,603	-2,130	
0,641	1,204	0,303	-0,016	-0,001	0,236	-0,150	XR_106831
0,273	0,697	-0,876	-1,020	-1,429	-1,477	-1,390	NM_001168660
0,779	-0,254	-1,352	-0,415	-1,224	-0,579	-1,032	NM_028593
-0,973	-0,321	-2,845	-2,317	-2,153	-2,197	-2,250	NM_001033460
0,465	-0,448	-1,004	-1,477	-1,039	-0,882	-1,124	
0,917	0,423	-0,989	-0,423	-0,552	-0,437	-0,683	
0,383	0,243	-1,675	-0,243	-0,755	-1,092	-0,982	
0,900	-0,266	-0,980	-0,729	-0,972	0,556	-0,948	
1,481	0,918	0,000	-0,161	-1,117	0,400	-0,632	
-0,320	-2,376	-1,658	-1,857	-1,888	-2,131	-2,509	NM_144551
-0,691	-0,732	-1,121	-1,698	-1,858	-1,436	-2,143	

1,756	1,030	0,179	-0,210	1,474	0,342	-0,072	
0,166	0,966	-1,139	-1,488	-0,123	-1,264	-0,504	NM_001081020
4,331	3,826	2,135	2,189	2,591	2,705	2,276	NM_028408
-1,126	-1,533	-2,464	-1,876	-2,399	-2,112	-2,425	NM_001162938
0,086	0,139	-0,553	-1,193	-1,206	-0,636	-1,799	
1,509	0,989	0,223	-0,025	0,086	0,401	-0,033	
-1,869	-1,968	-3,252	-3,162	-3,150	-4,143	-3,091	NM_001162938
1,201	2,806	1,826	1,052	-0,320	0,942	0,610	NM_015755
0,299	0,779	-0,920	-0,910	-1,260	-1,251	-1,219	NM_173786
-1,250	-0,882	-1,326	-1,231	-2,108	-2,175	-3,232	
0,207	-0,331	-1,268	-1,178	-1,017	-0,048	-0,391	
3,161	1,298	1,346	1,256	0,795	2,337	0,232	NM_009317
-1,003	-0,063	-2,492	-2,236	-1,619	-1,455	-2,062	NM_144796
1,006	-0,582	-0,540	-0,781	-0,707	-0,411	-0,948	
2,089	2,848	1,064	0,859	1,398	1,647	0,893	NM_008006
1,010	0,357	0,490	0,227	0,045	0,456	-0,078	NM_001039244
0,667	-0,052	-0,445	-0,520	-0,497	-0,580	-1,309	NM_001011687
-0,587	-1,470	-2,285	-1,871	-0,957	-1,825	-2,505	
-0,459	-1,516	-1,949	-2,340	-1,777	-1,523	-1,745	
-0,392	-0,341	-1,420	-0,032	-0,470	-1,155	-1,137	NM_133654
1,065	-0,207	-0,651	-0,824	-0,222	-0,131	-0,627	NR_024323
0,279	-0,740	-1,421	-1,086	-1,195	-1,196	-0,819	NM_198306
-0,552	0,016	-1,998	-0,674	-1,049	-2,358	-0,442	NM_001164518
1,547	0,940	0,197	-0,022	0,406	0,691	1,000	NM_013869
0,071	-0,831	-0,824	-1,136	-2,151	-0,708	-1,537	
0,180	-0,199	-0,992	0,159	-1,071	-0,620	-1,017	
0,033	0,158	-1,141	-1,145	-1,297	-1,019	-0,543	
-0,222	0,145	-2,216	-1,809	-0,772	-1,372	-1,015	NM_011374
0,864	1,189	-0,300	0,745	-0,253	0,470	-0,053	
-2,269	-0,945	-2,597	-3,054	-3,907	-2,988	-2,033	NM_001033378
1,140	1,108	-1,187	0,009	0,109	-0,121	0,796	
-0,385	-0,597	-1,507	-0,721	-1,848	-1,848	-1,200	
1,111	2,397	0,762	0,568	0,876	1,142	0,327	NM_007992
-0,053	0,219	-0,422	-0,783	-0,989	-0,855	-1,114	NM_008923
0,637	1,416	0,143	0,089	-1,045	0,177	-0,558	
0,481	0,631	0,038	-0,440	-1,426	-0,211	-1,422	
0,782	0,631	0,540	-0,230	-1,451	-1,151	0,492	
0,405	-0,485	-0,849	-0,850	-1,020	-0,288	-0,857	NM_001197321
0,089	0,111	-1,458	-0,916	-0,667	-0,977	-1,580	
0,216	0,714	-1,276	-0,865	-0,754	-0,360	-1,123	NM_001024617
1,633	1,484	0,100	-0,507	0,477	0,425	0,466	NM_016846
-1,312	-0,291	-1,519	-1,175	-1,561	-1,530	-1,537	
-0,887	-0,552	-1,286	-1,592	-1,611	-1,316	-2,028	NM_010357
-1,955	-0,855	-2,734	-2,911	-2,145	-2,070	-2,548	NR_003967
-0,006	-0,190	-1,032	-1,163	-0,760	-0,751	-1,136	NM_008606
0,097	-0,204	-0,710	-0,703	-1,028	-0,719	-0,453	
2,266	2,177	-0,005	1,698	1,627	1,158	-0,006	
-0,499	-1,409	-1,518	-1,225	-1,708	-1,920	-1,421	
-0,255	0,045	-1,428	-1,005	-0,654	-0,749	-1,201	NM_008606
0,639	-0,325	-0,544	-0,396	-0,332	-0,479	-0,165	
-0,568	0,074	-1,907	-0,941	-1,902	-0,822	-0,699	
1,004	1,208	-0,150	0,385	0,523	-0,008	-0,070	NM_172392
-0,203	0,917	-0,654	-0,019	-0,949	-0,268	-0,573	NM_133249

GeneID
434215
100033459
170942
229499
98660
100033459
27049
11801
382571
71492
382571
192166
633640
19341
74720
109246
67701
19341
233332
229474
110197
241075
21473
223672
633640
100044171
71898
73649
381738
75842
217410

108154
72978
100033459

100033459
26559
223672

21350
96935

14173
633640
497071

12490
74629
231605
210094
29820

20449
14114
319493

14115
19085

108655

234515
19731

14860
209380
17385

17385

627872
268670
170826

SeqID	ProbeID	GeneName
2277855	A_55_P2106690	Tal1
2263973	A_51_P364485	Tnfaip2
2279870	A_55_P2098697	Tnfaip2
2298843	A_51_P188271	Cd248
2306905	A_55_P2028365	Lrrc36
2263599	A_51_P142896	Cd59a
2309813	A_52_P244193	Cd24a
2263382	A_55_P1958275	Bcl11a
2284426	A_55_P2214124	Igh-VJ558
2287004	A_55_P2052240	Crlf1
2311495	A_55_P1995404	Cd59b
2304506	A_52_P219473	Cdc6
2276432	A_55_P1988872	ENSMUST0000010
2279875	A_55_P2003915	ENSMUST0000010
2274189	A_51_P234140	Cdkl1
2309975	A_51_P407657	ENSMUST0000010
2264330	A_55_P2075510	ENSMUST0000010
2279664	A_51_P339793	Il1rl1
2262785	A_51_P204913	Bag2
2299838	A_51_P481920	Ccna2
2274720	A_55_P2065671	Ccnb1
2288815	A_51_P170807	Map3k6
2267381	A_52_P296913	Cnnm1
2304290	A_55_P1956718	Rab38
2273712	A_51_P408946	Ccne1
2271387	A_51_P253803	Mki67
2272460	A_55_P1960148	AK007826
2306362	A_55_P2130497	ENSMUST0000010
2261055	A_55_P2030299	LOC544904
2281467	A_66_P114695	Cdc25c
2300786	A_51_P457528	Ccnb2
2309991	A_55_P2140348	Igh-VJ558
2264083	A_55_P2129092	Cdc42bpa
2282178	A_51_P487690	Ifi44
2293227	A_55_P2039324	Ccnf
2301420	A_66_P125327	Ccne1
2289773	A_55_P1952256	Ccnb1
2301481	A_66_P128434	Kit
2308773	A_51_P405638	LOC100045791
2259200	A_52_P30641	ENSMUST0000010
2274816	A_55_P2011950	LOC435333
2288305	A_55_P2079079	Cd19
2267667	A_55_P2075393	LOC100047132
2260536	A_55_P2096395	Cdc42bpa
2302604	A_51_P137111	Chek2
2278881	A_52_P30989	Cdkn3

2302014	A_51_P155142	Cdca8
2286831	A_51_P427917	Il31ra
2290025	A_55_P1975874	Bcl2l15
2264647	A_55_P1959421	ENSMUST0000010
2302700	A_55_P2092869	Anxa9
2267713	A_52_P112190	LOC100047053
2303584	A_52_P451073	Tnfrsf21
2289563	A_55_P1977451	ENSMUST0000010
2295190	A_55_P2096402	Cdc42bpa
2258389	A_55_P2087429	LOC100047162
2259508	A_55_P2170681	Cdca8
2300047	A_51_P312035	Cd300e
2303607	A_55_P2001267	XM_001472404
2311760	A_55_P2105878	Ccr10
2262726	A_51_P473170	Tnfsf4
2312288	A_51_P130095	Fcgr2b
2307080	A_51_P125135	Cdca5
2277727	A_52_P661044	Ccr3
2266724	A_55_P1996946	Cdc20
2265415	A_52_P186262	ENSMUST0000010
2270408	A_52_P546162	ENSMUST0000010
2291622	A_55_P1965050	Cks2
2258591	A_55_P1965338	Anxa8
2306096	A_51_P375146	Cd36
2262852	A_55_P2027737	Il1rl1
2285899	A_55_P2058028	ENSMUST0000010
2287578	A_55_P1986282	Cdkn1a
2266536	A_55_P2058023	ENSMUST0000010
2307552	A_51_P117581	Cables1
2279139	A_55_P2065510	ENSMUST0000010
2281798	A_55_P2066578	Ifi204
2310147	A_51_P284608	Cd74
2264800	A_51_P227004	Cks1b
2272946	A_55_P2092826	Anxa1
2295994	A_51_P241457	Lilrb4
2306283	A_55_P2137496	LOC100047815
2286855	A_55_P2061495	Cks1b
2279835	A_51_P271503	Il1r1

Description	ANOVA p-value
Mus musculus T-cell acute lymphocytic leukemia 1 (Tal1), mRNA [NM_011527]	2,68E-07
Mus musculus tumor necrosis factor, alpha-induced protein 2 (Tnfaip2), mRNA [NM_00	1,78E-07
Mus musculus tumor necrosis factor, alpha-induced protein 2 (Tnfaip2), mRNA [NM_00	1,85E-07
Mus musculus CD248 antigen, endosialin (Cd248), mRNA [NM_054042]	1,69E-08
Mus musculus leucine rich repeat containing 36 (Lrrc36), transcript variant 1, mRNA [N	2,39E-05
Mus musculus CD59a antigen (Cd59a), transcript variant 2, mRNA [NM_007652]	5,22E-07
Mus musculus CD24a antigen (Cd24a), mRNA [NM_009846]	8,58E-09
Mus musculus B-cell CLL/lymphoma 11A (zinc finger protein) (Bcl11a), transcript varian	4,65E-09
Mus musculus immunoglobulin heavy chain complex, mRNA (cDNA clone MGC:30346	1,89E-14
Mus musculus cytokine receptor-like factor 1 (Crlf1), mRNA [NM_018827]	3,84E-04
Mus musculus CD59b antigen (Cd59b), mRNA [NM_181858]	3,26E-04
Mus musculus cell division cycle 6 homolog (S. cerevisiae) (Cdc6), transcript variant 1,	3,08E-06
Mus musculus B-cell CLL/lymphoma 11A (zinc finger protein) (Bcl11a), transcript varian	2,63E-07
immunoglobulin kappa variable 6-13 [Source:MGI Symbol;Acc:MGI:1330829] [ENSMU	1,04E-09
Mus musculus cyclin-dependent kinase-like 1 (CDC2-related kinase) (Cdkl1), mRNA [NI	1,57E-02
immunoglobulin kappa chain variable 19-93 [Source:MGI Symbol;Acc:MGI:107617] [E	3,95E-09
immunoglobulin heavy variable V1-23 [Source:MGI Symbol;Acc:MGI:3815050] [ENSM	6,33E-10
Mus musculus interleukin 1 receptor-like 1 (Il1rl1), transcript variant 2, mRNA [NM_0107	6,08E-03
Mus musculus BCL2-associated athanogene 2 (Bag2), mRNA [NM_145392]	1,29E-06
Mus musculus cyclin A2 (Ccna2), mRNA [NM_009828]	1,06E-04
Mus musculus cyclin B1 (Ccnb1), mRNA [NM_172301]	3,92E-05
Mus musculus mitogen-activated protein kinase kinase kinase 6 (Map3k6), mRNA [NM	3,57E-04
Mus musculus cyclin M1 (Cnnm1), mRNA [NM_031396]	8,74E-06
Mus musculus RAB38, member of RAS oncogene family (Rab38), mRNA [NM_028238	1,15E-05
Mus musculus cyclin E1 (Ccne1), mRNA [NM_007633]	1,20E-05
Mus musculus antigen identified by monoclonal antibody Ki 67 (Mki67), mRNA [NM_00	4,09E-04
PREDICTED: Mus musculus immunoglobulin heavy chain (J558 family) (Igh-VJ558), m	4,37E-17
immunoglobulin heavy variable V1-67 [Source:MGI Symbol;Acc:MGI:3645228] [ENSM	1,13E-12
immunoglobulin heavy variable V14-3 [Source:MGI Symbol;Acc:MGI:4439764] [ENSM	1,50E-11
Mus musculus cell division cycle 25 homolog C (S. pombe) (Cdc25c), mRNA [NM_0098	2,94E-05
Mus musculus cyclin B2 (Ccnb2), mRNA [NM_007630]	1,15E-05
Mus musculus immunoglobulin heavy chain complex, mRNA (cDNA clone MGC:118142	9,82E-10
Mus musculus CDC42 binding protein kinase alpha (Cdc42bpa), mRNA [NM_0010332	7,75E-04
Mus musculus interferon-induced protein 44 (Ifi44), mRNA [NM_133871]	1,68E-07
Mus musculus cyclin F (Ccnf), mRNA [NM_007634]	2,23E-06
Mus musculus cyclin E1 (Ccne1), mRNA [NM_007633]	3,32E-06
Mus musculus cyclin B1 (Ccnb1), mRNA [NM_172301]	2,33E-05
Mus musculus kit oncogene (Kit), transcript variant 1, mRNA [NM_001122733]	5,64E-09
immunoglobulin heavy variable V9-3 [Source:MGI Symbol;Acc:MGI:3642720] [ENSMU	2,79E-08
immunoglobulin kappa variable 4-86 [Source:MGI Symbol;Acc:MGI:2685305] [ENSMU	5,59E-10
immunoglobulin heavy variable 1-85 [Source:MGI Symbol;Acc:MGI:3645723] [ENSMU	3,78E-11
Mus musculus CD19 antigen (Cd19), mRNA [NM_009844]	3,47E-04
immunoglobulin kappa variable 6-20 [Source:MGI Symbol;Acc:MGI:1330836] [ENSMU	2,95E-08
Mus musculus CDC42 binding protein kinase alpha (Cdc42bpa), mRNA [NM_0010332	4,05E-03
Mus musculus CHK2 checkpoint homolog (S. pombe) (Chek2), mRNA [NM_016681]	7,48E-05
Mus musculus cyclin-dependent kinase inhibitor 3 (Cdkn3), mRNA [NM_028222]	9,93E-05

Mus musculus cell division cycle associated 8 (Cdca8), mRNA [NM_026560]	4,03E-05
Mus musculus interleukin 31 receptor A (Il31ra), mRNA [NM_139299]	9,23E-04
Mus musculus BCLl2-like 15 (Bcl2l15), transcript variant 1, mRNA [NM_001142959]	9,86E-05
immunoglobulin kappa chain variable 6-29 [Source:MGI Symbol;Acc:MGI:4439616] [E	5,81E-09
Mus musculus annexin A9 (Anxa9), transcript variant 1, mRNA [NM_001085383]	3,20E-04
immunoglobulin kappa variable 6-32 [Source:MGI Symbol;Acc:MGI:3641634] [ENSMU	7,97E-11
Mus musculus tumor necrosis factor receptor superfamily, member 21 (Tnfrsf21), mRNA	9,26E-06
immunoglobulin kappa variable 4-80 [Source:MGI Symbol;Acc:MGI:4439653] [ENSMU	7,78E-13
Mus musculus CDC42 binding protein kinase alpha (Cdc42bpa), mRNA [NM_0010332	5,94E-03
immunoglobulin kappa variable 6-15 [Source:MGI Symbol;Acc:MGI:1330831] [ENSMU	1,17E-10
Mus musculus cell division cycle associated 8 (Cdca8), mRNA [NM_026560]	1,23E-05
Mus musculus CD300e antigen (Cd300e), mRNA [NM_172050]	2,48E-03
Mus musculus isolate 311 immunoglobulin heavy chain variable region mRNA, partial c	1,16E-09
Mus musculus chemokine (C-C motif) receptor 10 (Ccr10), mRNA [NM_007721]	5,02E-13
Mus musculus tumor necrosis factor (ligand) superfamily, member 4 (Tnfsf4), mRNA [NI	1,49E-11
Mus musculus Fc receptor, IgG, low affinity IIb (Fcgr2b), transcript variant 1, mRNA [NM	2,71E-07
Mus musculus cell division cycle associated 5 (Cdca5), mRNA [NM_026410]	2,26E-06
Mus musculus chemokine (C-C motif) receptor 3 (Ccr3), mRNA [NM_009914]	4,74E-06
Mus musculus cell division cycle 20 homolog (S. cerevisiae) (Cdc20), mRNA [NM_0232	8,23E-05
immunoglobulin kappa chain variable 6-25 [Source:MGI Symbol;Acc:MGI:4439867] [E	2,00E-13
immunoglobulin kappa variable 4-58 [Source:MGI Symbol;Acc:MGI:2685923] [ENSMU	2,53E-13
Mus musculus CDC28 protein kinase regulatory subunit 2 (Cks2), mRNA [NM_025415]	1,70E-06
Mus musculus annexin A8 (Anxa8), mRNA [NM_013473]	3,39E-04
Mus musculus CD36 antigen (Cd36), transcript variant 2, mRNA [NM_007643]	5,05E-05
Mus musculus interleukin 1 receptor-like 1 (Il1rl1), transcript variant 1, mRNA [NM_001(	4,12E-04
immunoglobulin kappa variable 4-63 [Source:MGI Symbol;Acc:MGI:3645235] [ENSMU	5,78E-12
Mus musculus cyclin-dependent kinase inhibitor 1A (P21) (Cdkn1a), transcript variant 2	4,30E-11
immunoglobulin kappa variable 4-57-1 [Source:MGI Symbol;Acc:MGI:2686264] [ENSM	1,55E-12
Mus musculus CDK5 and Abl enzyme substrate 1 (Cables1), transcript variant 2, mRNA	1,45E-09
immunoglobulin heavy variable V1-20 [Source:MGI Symbol;Acc:MGI:3644607] [ENSM	4,55E-11
Mus musculus interferon activated gene 204, mRNA (cDNA clone MGC:18551 IMAGE	4,84E-04
Mus musculus CD74 antigen (invariant polypeptide of major histocompatibility complex	7,57E-07
Mus musculus CDC28 protein kinase 1b (Cks1b), mRNA [NM_016904]	6,50E-06
Mus musculus annexin A1 (Anxa1), mRNA [NM_010730]	4,73E-08
Mus musculus leukocyte immunoglobulin-like receptor, subfamily B, member 4 (Lilrb4),	9,57E-07
Mus musculus CD79A antigen (immunoglobulin-associated alpha), mRNA (cDNA clone	1,06E-08
Mus musculus CDC28 protein kinase 1b (Cks1b), mRNA [NM_016904]	6,32E-06
Mus musculus interleukin 1 receptor, type I (Il1r1), transcript variant 1, mRNA [NM_008	2,03E-05

Adjusted ANOVA p-value	Tukey p-value	Fold change	Log2 ratio	Median	wt_non-stim_1	wt_non-stim_2	wt_non-stim_3	wt_non-stim_4
**1,58E-06**	**1,13E-05**	**19,932**	4,317	6,904	-0,406	-0,273	0,377	0,203
**1,09E-06**	**7,87E-06**	**19,576**	4,291	7,822	-0,367	-0,383	-0,221	0,343
**1,13E-06**	**1,10E-05**	**15,584**	3,962	7,023	-0,652	-0,460	-0,079	0,214
**1,29E-07**	**3,18E-05**	**12,978**	3,698	4,184	1,173	1,159	0,780	0,597
**9,68E-05**	**1,83E-04**	**12,711**	3,668	3,620	*-0,050*	*-0,705*	*-0,261*	0,229
**2,89E-06**	**1,52E-04**	**11,786**	3,559	4,159	0,113	0,063	-0,113	0,416
**7,06E-08**	**5,41E-06**	**11,592**	3,535	8,841	0,657	0,454	0,691	0,649
**4,09E-08**	**3,71E-05**	**9,389**	3,231	7,075	0,257	0,501	0,535	1,049
**1,36E-12**	**7,06E-06**	**8,497**	3,087	10,262	3,018	1,568	2,828	2,732
**1,26E-03**	**2,01E-04**	**8,421**	3,074	6,711	-0,946	-1,138	-0,646	-1,289
**1,09E-03**	**1,20E-03**	**7,923**	2,986	2,134	*0,004*	*-0,002*	*0,017*	*-0,041*
**1,47E-05**	**1,48E-05**	**6,788**	2,763	7,696	-2,994	-2,808	-2,895	-2,794
**1,55E-06**	**2,39E-03**	**6,212**	2,635	5,739	0,271	0,266	-0,036	0,318
**1,09E-08**	**7,14E-03**	**5,352**	2,420	6,521	2,057	0,600	0,891	2,141
**3,80E-02**	**3,94E-02**	**5,348**	2,419	2,138	*-0,248*	*1,103*	*-0,266*	*-0,249*
**3,54E-08**	**3,33E-02**	**4,857**	2,280	9,436	1,654	1,455	2,844	1,827
**7,07E-09**	**1,64E-02**	**4,827**	2,271	5,936	1,318	2,745	2,574	0,959
**1,60E-02**	**1,32E-02**	**4,656**	2,219	4,575	-0,444	*-1,375*	*-1,526*	*-2,263*
**6,63E-06**	**1,55E-05**	**4,579**	2,195	9,061	-2,537	-2,325	-2,336	-2,193
**3,85E-04**	**3,43E-04**	**4,380**	2,131	11,879	-2,097	-1,652	-1,966	-2,189
**1,53E-04**	**1,04E-04**	**4,356**	2,123	10,940	-1,999	-1,615	-1,900	-2,117
**1,18E-03**	**1,09E-03**	**4,272**	2,095	4,772	*-1,601*	-0,721	-0,853	-0,718
**3,84E-05**	**5,44E-05**	**4,249**	2,087	5,115	*-3,224*	*-2,341*	*-3,237*	*-2,184*
**4,93E-05**	**5,97E-04**	**4,187**	2,066	6,362	-0,076	-0,285	0,275	0,321
**5,13E-05**	**1,46E-04**	**4,155**	2,055	12,326	-2,463	-2,166	-2,369	-2,242
**1,34E-03**	**2,02E-04**	**4,056**	2,020	12,761	-1,437	-1,177	-1,433	-1,673
**1,94E-14**	**1,37E-06**	**4,053**	2,019	14,047	2,644	1,617	2,475	2,367
**3,41E-11**	**5,07E-04**	**3,840**	1,941	9,697	1,345	1,539	2,148	1,650
**2,83E-10**	**3,08E-03**	**3,558**	1,831	11,483	1,607	1,342	2,012	1,514
**1,17E-04**	**1,50E-04**	**3,543**	1,825	7,105	-1,830	-1,678	-2,401	-2,067
**4,93E-05**	**9,62E-05**	**3,465**	1,793	11,146	-0,421	-0,084	-0,239	-0,326
**1,04E-08**	**3,13E-02**	**3,425**	1,776	7,989	1,482	1,615	1,944	1,574
**2,42E-03**	**1,47E-03**	**3,385**	1,759	3,974	*-0,942*	*-2,188*	*-1,052*	*-1,431*
**1,03E-06**	**2,29E-03**	**3,340**	1,740	3,951	0,262	-0,208	0,239	1,469
**1,09E-05**	**2,52E-04**	**3,317**	1,730	9,819	-2,287	-2,450	-2,532	-2,478
**1,58E-05**	**5,54E-04**	**3,276**	1,712	7,250	-3,121	-2,421	-2,664	-2,958
**9,46E-05**	**1,32E-04**	**3,251**	1,701	8,579	-1,749	-1,434	-1,812	-1,868
**4,85E-08**	**3,32E-05**	**3,240**	1,696	7,847	-0,029	0,068	0,163	0,029
**2,02E-07**	**4,63E-02**	**3,184**	1,671	8,565	1,359	1,864	1,294	0,694
**6,34E-09**	**4,23E-02**	**3,180**	1,669	8,696	2,370	1,584	2,512	0,951
**6,22E-10**	**1,28E-02**	**3,173**	1,666	10,629	1,808	1,686	2,282	1,798
**1,15E-03**	**2,18E-02**	**3,147**	1,654	8,944	-0,401	-1,145	-1,150	-1,302
**2,13E-07**	**4,70E-02**	**3,091**	1,628	6,601	1,137	0,832	1,381	0,930
**1,10E-02**	**2,36E-03**	**3,078**	1,622	5,332	-0,233	-0,935	-0,645	-0,948
**2,78E-04**	**5,40E-04**	**3,046**	1,607	6,216	-1,764	-1,720	-1,736	-2,388
**3,62E-04**	**6,98E-04**	**3,036**	1,602	8,032	-0,126	-0,059	-0,301	-0,338

1,57E-04	1,65E-04	3,031	1,600	12,619	-1,579	-1,235	-1,541	-1,606
2,84E-03	3,41E-02	2,992	1,581	3,200	-1,353	0,672	-0,053	-0,321
3,60E-04	2,41E-02	2,986	1,578	6,002	0,380	0,322	0,435	0,132
4,98E-08	1,84E-02	2,977	1,574	6,555	1,105	0,779	1,369	0,998
1,07E-03	1,58E-04	2,971	1,571	5,598	-0,706	-0,442	-0,777	-0,549
1,18E-09	3,33E-02	2,924	1,548	8,699	1,668	1,145	2,032	1,818
4,05E-05	2,40E-03	2,916	1,544	4,859	-2,978	-1,555	-2,034	-2,396
2,52E-11	7,06E-03	2,807	1,489	10,602	2,233	1,163	2,355	1,604
1,57E-02	2,43E-02	2,738	1,453	4,859	-2,027	-1,032	-3,061	-1,306
1,63E-09	4,97E-03	2,706	1,436	9,445	1,843	1,167	1,892	1,830
5,25E-05	1,45E-04	2,668	1,416	10,229	-1,735	-1,390	-1,637	-1,997
7,06E-03	4,75E-02	2,667	1,415	2,386	0,388	-0,557	-0,559	1,572
1,20E-08	7,17E-03	2,663	1,413	5,257	1,937	1,419	2,663	1,904
1,75E-11	2,71E-03	2,661	1,412	5,111	2,524	1,982	2,057	1,776
2,81E-10	1,50E-02	2,617	1,388	5,732	-2,449	-3,097	-3,087	-3,786
1,59E-06	1,98E-02	2,617	1,388	5,301	-0,075	0,010	-0,055	-0,010
1,11E-05	4,90E-04	2,530	1,339	12,369	-2,025	-1,757	-2,036	-2,208
2,19E-05	4,60E-02	2,526	1,337	5,021	0,182	0,276	0,123	0,151
3,04E-04	1,27E-04	2,521	1,334	12,441	-1,269	-1,040	-1,186	-1,356
8,28E-12	1,99E-04	2,497	1,320	9,090	1,975	1,343	2,008	2,194
1,00E-11	2,74E-03	2,474	1,307	10,069	2,247	1,337	2,467	1,597
8,51E-06	1,68E-04	2,367	1,243	9,031	-1,654	-1,842	-1,994	-1,702
1,12E-03	1,63E-02	2,312	1,209	3,914	-2,151	-0,634	-1,360	-0,204
1,94E-04	8,81E-03	2,307	1,206	3,908	-0,171	-0,819	-1,091	-0,054
1,35E-03	6,00E-03	2,286	1,193	9,511	-0,978	-0,860	-0,947	-0,889
1,26E-10	2,60E-02	2,247	1,168	11,099	2,184	1,467	2,889	1,880
6,91E-10	1,46E-02	2,215	1,147	6,361	-4,350	-2,982	-1,668	-3,607
4,43E-11	1,52E-02	2,181	1,125	11,259	2,071	1,148	2,661	1,842
1,46E-08	6,01E-05	2,172	1,119	7,342	0,032	-0,032	-0,176	-0,429
7,26E-10	4,92E-02	2,169	1,117	9,689	1,768	1,642	2,070	1,462
1,56E-03	1,90E-02	2,169	1,117	4,098	-0,202	-1,672	-0,525	-2,217
4,06E-06	8,71E-03	2,169	1,117	13,958	0,214	-0,165	-0,093	-0,237
2,93E-05	3,05E-04	2,139	1,097	12,494	-1,524	-1,345	-1,465	-1,570
3,26E-07	3,72E-02	2,130	1,091	9,126	0,154	-0,088	0,270	0,088
5,04E-06	4,02E-02	2,126	1,088	6,723	-0,851	-1,387	-1,173	-2,056
8,52E-08	4,84E-02	2,094	1,066	9,802	0,552	0,047	0,130	0,074
2,85E-05	3,95E-04	2,091	1,064	12,167	-1,553	-1,425	-1,498	-1,711
8,33E-05	4,50E-03	2,075	1,053	5,696	-0,282	-0,409	-0,562	-0,442

wt_non-stim_21	CD4creGARPfl-fl_non-stim_22	CD4creGARPfl-fl_non-stim_23	CD4creGARPfl-fl_non-stim_8	CD4creGARPfl-fl_non-stim_9	CD4creGARPfl-fl_non-stim_10	wt_stim_11	wt_stim_12	wt_stim_13	wt_stim_14
1,034	5,869	1,919	4,273	4,520	5,300	-1,539	-1,775	-0,780	-1,234
0,904	5,478	1,834	4,070	3,937	4,968	-1,483	-1,926	-1,076	-1,234
0,638	5,421	1,686	3,882	3,811	4,758	-1,608	-2,355	-1,137	-1,565
1,661	5,917	2,399	4,857	4,597	5,558	-0,597	-1,047	-2,317	-0,711
0,557	5,249	1,291	3,502	3,619	4,794	-1,500	-0,010	-0,976	-0,016
0,920	5,375	1,588	3,672	3,323	4,620	-2,143	-2,152	-2,147	-1,664
1,448	5,430	2,168	3,539	4,192	4,816	-0,917	-0,983	-0,833	-0,736
1,678	4,606	1,969	3,766	3,490	4,401	-2,078	-2,313	-1,745	-2,350
2,669	4,774	5,291	5,818	6,266	5,838	-5,493	-5,571	-5,472	-5,730
-1,099	3,304	-0,520	1,185	1,975	2,720	-0,047	0,012	0,117	0,292
0,010	4,466	-0,020	1,900	2,990	3,656	-0,099	-0,074	0,002	-0,038
-1,871	1,423	-1,449	-0,353	-0,045	0,983	0,074	0,107	0,198	-0,019
1,752	3,543	1,491	2,906	2,590	3,309	-2,071	-1,849	-2,134	-3,884
0,328	3,645	3,311	2,523	3,432	2,245	-2,852	-4,082	-3,357	-4,414
0,351	2,825	-0,255	0,801	2,171	2,922	-0,238	0,146	-0,239	0,745
0,890	2,156	3,934	3,344	5,025	4,313	-4,688	-4,925	-4,834	-4,275
1,893	2,544	4,165	4,680	4,288	2,705	-4,167	-3,386	-4,169	-2,157
0,623	1,601	-0,138	0,191	1,879	0,843	-0,432	-0,117	0,117	-0,803
-1,942	1,098	-1,400	-0,130	-0,376	0,499	-0,019	0,019	0,089	0,053
-0,904	1,431	-1,027	-0,419	0,166	0,746	0,004	0,011	-0,054	0,007
-0,875	1,352	-0,953	0,070	0,223	0,965	-0,166	-0,070	-0,218	-0,290
-0,407	1,851	0,190	1,375	1,267	1,663	-0,050	-0,714	-0,433	-0,418
-1,846	1,534	-1,101	-0,282	-0,254	1,065	0,259	-0,243	0,466	0,314
0,872	2,564	0,517	2,341	1,812	2,552	-0,667	-0,911	-0,095	-0,816
-1,715	1,035	-1,663	-0,339	-0,187	0,720	0,081	0,030	-0,014	0,072
-0,004	1,904	0,004	0,235	0,588	1,216	-0,428	-0,289	-0,363	-0,368
2,430	4,036	4,285	4,449	4,594	4,503	-5,922	-5,647	-5,419	-5,287
1,314	2,704	3,481	3,532	3,860	3,470	-3,898	-3,622	-3,411	-4,278
1,198	2,619	3,058	3,345	3,777	3,843	-3,923	-3,943	-3,733	-4,048
-0,696	1,274	-0,706	-0,413	-0,005	0,538	-0,028	-0,113	0,005	0,029
0,788	2,750	0,940	1,214	1,553	2,257	-0,374	-0,270	-0,418	-0,598
1,237	2,012	3,292	3,435	3,968	3,350	-2,939	-3,497	-3,325	-4,284
-1,259	1,062	-1,002	0,501	0,290	0,987	-0,040	0,241	-0,280	-0,534
0,531	2,002	1,971	2,096	2,009	1,795	-0,901	-0,576	-2,062	-2,062
-1,402	0,752	-1,363	-1,014	-0,720	0,082	0,023	0,042	-0,023	-0,036
-2,009	0,888	-2,031	-0,952	-0,965	0,029	0,288	0,398	0,312	0,442
-0,957	1,338	-0,836	-0,105	-0,047	0,799	-0,014	0,268	0,138	0,003
0,961	1,925	1,478	1,765	1,419	1,837	-0,913	-1,227	-1,049	-1,256
0,354	2,964	1,850	3,756	2,859	3,030	-3,395	-3,356	-3,499	-3,169
1,124	2,582	2,986	3,304	3,903	3,253	-3,112	-3,819	-3,368	-5,135
1,395	2,375	3,464	3,472	4,076	3,420	-3,737	-3,716	-3,232	-3,893
0,198	0,011	0,674	0,297	0,509	1,431	-0,883	-1,777	-1,334	-2,100
0,749	1,851	2,559	3,108	3,381	2,017	-4,704	-3,734	-2,423	-2,222
-0,381	1,798	-0,561	0,978	0,937	1,529	0,036	0,168	0,298	-0,360
-1,041	1,383	-1,029	-0,370	-0,129	0,799	0,415	0,525	0,129	0,454
0,972	2,863	0,865	0,997	1,476	2,224	-0,290	-0,180	-0,378	-0,460

-0,589	1,091	-0,561	-0,165	0,059	0,721	-0,005	0,005	-0,068	-0,086
1,493	2,064	1,403	1,661	1,214	1,528	0,053	-1,361	-1,378	0,387
0,191	2,693	-0,132	1,361	1,900	2,635	-1,325	-0,527	-1,341	-0,901
0,790	1,855	2,573	3,293	3,233	1,943	-4,708	-2,987	-3,381	-2,015
-0,674	1,899	-0,270	0,897	0,800	1,843	-0,108	0,007	0,050	0,105
1,723	1,627	2,358	4,006	3,322	3,272	-2,993	-3,457	-2,961	-3,839
-1,259	0,495	-0,838	-0,587	-0,489	-0,272	0,747	1,044	0,730	0,780
1,306	2,662	3,093	2,935	3,157	3,314	-3,771	-4,307	-3,518	-4,867
-0,761	1,378	-1,774	0,147	-0,472	1,020	-0,035	0,035	0,425	0,705
0,853	4,601	2,682	3,380	3,266	3,175	-3,498	-2,870	-4,622	-4,601
-0,840	0,936	-0,741	-0,427	-0,222	0,401	-0,007	0,092	0,007	-0,060
0,103	0,051	1,517	1,285	1,622	2,561	-0,362	-0,564	-0,555	-0,558
1,153	2,724	3,216	3,317	5,033	4,895	-3,404	-3,419	-2,174	-3,416
2,274	2,979	4,208	3,468	3,583	3,253	-3,198	-2,415	-2,581	-1,776
-2,342	-2,156	-1,215	-1,700	-2,549	-1,646	1,890	2,326	2,109	2,759
1,178	1,493	1,135	1,070	1,377	1,795	-1,877	-2,223	-1,662	-2,274
-1,178	0,415	-1,251	-0,741	-0,686	0,092	0,009	0,017	-0,009	-0,011
1,664	2,024	2,118	1,219	1,307	1,519	-0,847	-0,746	-1,086	-1,262
-0,454	1,147	-0,431	0,020	0,148	0,766	-0,121	-0,020	-0,107	-0,213
1,167	2,673	3,296	3,703	4,046	3,229	-3,297	-3,423	-3,380	-3,701
1,326	2,382	2,903	2,803	3,416	3,604	-3,185	-3,584	-3,159	-3,930
-1,213	0,358	-1,279	-0,577	-0,460	0,102	0,150	0,144	0,062	0,053
-1,465	0,288	-0,152	-0,750	-0,061	-0,266	0,255	0,061	0,449	0,387
-0,035	1,886	0,035	0,191	1,035	1,513	-0,765	-0,617	-1,976	-1,401
0,304	0,911	0,304	-0,020	0,024	0,705	-0,559	-0,429	-0,381	-1,053
1,631	3,048	2,895	2,697	3,625	3,859	-3,602	-3,734	-3,230	-3,612
-2,807	-1,834	-1,684	-2,085	-2,053	-1,642	1,732	2,103	1,987	2,479
1,371	2,685	2,966	2,863	3,084	3,499	-3,750	-4,023	-3,357	-4,203
0,505	0,983	1,250	1,087	0,993	1,476	-1,452	-1,707	-1,407	-2,022
1,372	2,605	2,584	2,759	3,422	2,923	-4,075	-3,135	-3,353	-3,420
-0,146	0,565	0,891	0,592	0,724	0,062	-1,417	-0,650	-0,062	-1,646
0,469	0,547	1,034	0,769	1,023	1,412	-1,066	-1,526	-1,379	-1,869
-0,822	0,445	-0,927	-0,368	-0,436	0,305	0,052	0,016	-0,011	-0,018
1,476	1,397	1,856	1,245	1,192	0,971	-1,957	-2,269	-1,699	-2,408
-1,136	0,087	0,240	-0,656	-0,940	-0,086	0,086	0,334	0,293	-0,270
0,906	0,572	1,124	1,196	1,494	1,953	-2,315	-3,083	-2,604	-3,093
-0,803	0,384	-1,008	-0,433	-0,448	0,227	0,002	0,001	-0,089	-0,001
-0,369	0,873	1,132	0,644	0,563	0,264	-0,370	-0,481	-0,264	-0,556

wt_stim_15	CD4creGARPfl-fl_stim_24	CD4creGARPfl-fl_stim_25	CD4creGARPfl-fl_stim_18	CD4creGARPfl-fl_stim_19	CD4creGARPfl-fl_stim_20	Refseq	GeneID
-0,053	0,178	-1,172	0,053	-0,175	-0,476	NM_011527	21349
-1,224	1,003	0,093	-0,093	-0,947	0,518	NM_009396	21928
-1,901	1,034	0,079	-0,252	-0,931	0,570	NM_009396	21928
-1,190	-1,380	-0,898	-2,337	-0,727	-0,951	NM_054042	70445
-1,110	0,941	0,010	1,990	-1,011	-0,045	NM_001170788	270091
-1,219	0,597	-0,063	-0,146	-2,134	-1,125	NM_007652	12509
-0,886	-0,490	-0,954	-0,748	-1,037	-0,454	NM_009846	12484
-1,139	-1,061	-1,366	-0,504	-1,245	-0,257	NM_016707	14025
-7,360	-1,568	-1,831	-2,058	-2,949	-2,655		16061
0,645	-0,255	-0,311	0,190	-0,012	0,050	NM_018827	12931
0,173	-0,089	-0,060	0,028	-0,037	-0,016	NM_181858	333883
0,235	-0,293	0,019	0,106	0,325	0,138	NM_011799	23834
-1,622	-0,971	-2,158	-0,398	-1,188	0,036	NM_001242934	14025
-3,461	-0,328	-1,277	-2,753	-2,004	-1,512		
-0,243	-0,246	-0,146	0,921	-0,258	0,396	NM_183294	71091
-3,809	-4,393	-1,253	-3,103	-0,890	-0,955		
-4,171	-1,337	-1,927	-1,862	-2,527	-0,959		
-1,079	1,425	1,576	-0,272	0,451	0,579	NM_010743	17082
-0,258	0,301	0,511	0,074	0,366	0,160	NM_145392	213539
0,690	-0,046	-0,004	0,180	0,324	0,130	NM_009828	12428
0,521	0,369	0,274	0,222	0,443	0,432	NM_172301	268697
-2,939	0,505	0,520	0,263	0,483	0,050	NM_016693	53608
1,303	-0,015	0,140	0,375	0,086	0,015	NM_031396	83674
-0,017	0,204	-0,458	0,017	-0,231	-0,065	NM_028238	72433
0,489	-0,176	0,014	0,437	0,370	0,421	NM_007633	12447
0,458	-0,104	0,024	0,023	0,068	0,190	NM_001081117	17345
-5,894	-1,650	-1,980	-1,617	-2,899	-2,458	XM_001472091	
-5,212	-1,314	-2,296	-3,040	-1,944	-1,653		
-5,808	-1,198	-2,637	-3,200	-2,271	-1,435		
0,151	0,291	0,325	0,231	0,416	0,233	NM_009860	12532
0,135	0,035	-0,015	-0,308	0,059	0,015	NM_007630	12442
-5,994	-1,237	-2,117	-3,417	-1,662	-1,510		16061
1,090	0,020	-0,020	0,510	0,712	0,260	NM_001033285	226751
-2,059	0,208	-1,101	-0,894	-0,622	-0,497	NM_133871	99899
0,592	0,247	0,215	0,350	0,356	0,439	NM_007634	12449
-0,175	0,108	0,524	-0,029	0,714	0,464	NM_007633	12447
0,029	0,538	0,543	-0,003	0,626	0,433	NM_172301	268697
-1,262	0,195	-0,103	-0,876	-0,869	-0,701	NM_001122733	16590
-6,528	-0,354	-2,295	-2,828	-1,515	-1,254		
-4,127	-2,044	-1,977	-3,782	-0,951	-1,221		
-5,938	-1,395	-2,121	-2,865	-1,665	-1,516		
-0,380	0,495	0,165	-0,011	0,613	1,809	NM_009844	12478
-3,321	-0,749	-2,246	-3,728	-0,934	-2,147		
0,714	0,360	-0,036	-0,060	0,114	-0,788	NM_001033285	226751
-0,247	0,447	0,467	-0,168	0,447	0,370	NM_016681	50883
0,041	0,326	-0,041	-0,233	0,188	0,270	NM_028222	72391

0,560	0,168	0,260	0,286	0,414	0,481	NM_026560	52276
-1,360	-0,626	-0,675	-0,139	0,206	-1,344	NM_139299	218624
-0,373	0,416	-0,595	-1,074	-0,786	-0,507	NM_001142959	229672
-2,439	-0,779	-2,078	-2,746	-1,239	-1,612		
-0,103	0,258	0,133	-0,011	0,204	-0,007	NM_001085383	71790
-4,742	-1,145	-1,943	-1,712	-1,312	-1,210		
-1,022	0,846	1,063	0,272	0,990	0,623	NM_178589	94185
-5,181	-1,501	-1,994	-2,609	-1,827	-1,163		
0,539	0,466	0,461	-0,522	0,476	-0,227	NM_001033285	226751
-5,720	-0,853	-1,889	-2,309	-1,432	-1,618		
0,069	0,159	0,317	0,073	0,475	0,393	NM_026560	52276
-0,551	-0,229	-0,463	0,232	-0,051	0,668	NM_172050	217306
-3,402	-1,166	-2,458	-3,416	-1,153	-1,260		
-3,220	-2,363	-3,282	-3,236	-2,027	-3,296	NM_007721	12777
1,215	2,091	2,267	1,547	2,126	1,933	NM_009452	22164
-2,545	1,102	0,279	-0,636	-0,466	1,180	NM_001077189	14130
0,645	0,074	0,178	0,343	0,516	0,513	NM_026410	67849
-2,623	-0,123	-0,630	-1,255	-1,795	-0,843	NM_009914	12771
0,212	0,332	0,338	0,024	0,229	0,238	NM_023223	107995
-4,463	-1,286	-1,621	-2,185	-1,167	-1,456		
-3,513	-1,664	-2,216	-2,311	-1,527	-1,326		
0,036	-0,036	0,189	-0,107	0,242	0,247	NM_025415	66197
0,301	0,067	0,363	-0,152	0,423	0,431	NM_013473	11752
-1,934	0,721	0,638	0,644	0,198	0,622	NM_007643	12491
-0,616	0,994	0,865	0,020	0,130	0,455	NM_001025602	17082
-5,271	-1,467	-2,356	-2,439	-1,499	-1,475		
1,642	2,663	2,222	2,028	1,873	2,193	NM_001111099	12575
-5,120	-1,572	-2,330	-2,549	-1,987	-1,148		
-1,495	0,146	0,129	-0,614	-0,367	0,152	NM_022021	63955
-5,724	-1,372	-2,095	-3,140	-1,894	-1,869		
-2,218	1,571	0,634	0,125	0,491	1,314		15951
-0,818	0,446	0,064	-0,149	-0,064	1,060	NM_001042605	16149
0,283	0,011	0,140	0,153	0,311	0,383	NM_016904	54124
-2,686	0,378	-0,287	-1,088	-0,881	-0,806	NM_010730	16952
-0,519	3,099	2,177	0,986	1,404	1,954	NM_013532	14728
-1,984	-1,010	-1,520	-1,550	-1,440	-0,047		12518
0,214	0,009	0,207	0,166	0,313	0,294	NM_016904	54124
-1,746	1,275	1,776	0,575	0,701	0,692	NM_008362	16177

SeqID	ProbeID	GeneName
2276496	A_51_P460954	Ccl6
2268264	A_51_P413866	Cfb
2272748	A_51_P509679	Igh-VJ558
2272460	A_55_P1960148	AK007826
2284426	A_55_P2214124	Igh-VJ558
2309975	A_51_P407657	ENSMUST0000010
2264395	A_66_P139367	ENSMUST0000010
2261863	A_55_P2094060	Gzma
2276337	A_55_P1998011	Klra23
2266123	A_55_P2038987	Gas7
2262206	A_55_P2186942	ENSMUST0000010
2290842	A_55_P1979147	Klrb1c
2276883	A_52_P157837	ENSMUST0000010
2285927	A_66_P105596	LOC676193
2258389	A_55_P2087429	LOC100047162
2277494	A_52_P165705	Cd1d2
2271120	A_52_P161488	Clec4e
2288714	A_51_P435671	LOC631064
2271192	A_55_P2071716	Klre1
2287527	A_52_P272778	Klrk1
2269354	A_55_P2077958	Klra5
2288811	A_55_P2139356	LOC631518
2269908	A_55_P1997997	Klra23
2312019	A_66_P114582	ENSMUST0000010
2259450	A_55_P2183369	ENSMUST0000010
2309470	A_51_P212782	Il1b
2312288	A_51_P130095	Fcgr2b
2302264	A_52_P265556	ENSMUST0000010
2278988	A_55_P2079579	Pira7
2289563	A_55_P1977451	ENSMUST0000010
2284595	A_51_P212754	Tgfbi
2303935	A_55_P2138386	Il5
2287936	A_55_P2004536	Klra4
2281140	A_55_P2135526	Gzmc
2290409	A_52_P538084	ENSMUST0000010
2265811	A_51_P414879	ENSMUST0000010
2309485	A_51_P123625	Irg1
2306552	A_51_P303424	Itgax
2257024	A_55_P2099742	Ccl19
2264330	A_55_P2075510	ENSMUST0000010
2311735	A_52_P502849	ENSMUST0000010
2311641	A_51_P367070	Il9
2259452	A_51_P301394	Gspt2
2310444	A_52_P765764	ENSMUST0000010
2267512	A_52_P212686	Lrrk1
2304791	A_55_P2133907	ENSMUST0000010

2284193	A_55_P1988994	LOC100046350
2308283	A_55_P2015363	C1ra
2288477	A_51_P156274	Ltbr
2261598	A_55_P1978033	ENSMUST0000010
2279289	A_66_P102346	ENSMUST0000010
2267713	A_52_P112190	LOC100047053
2276126	A_55_P1979526	Cdkn2b
2291815	A_55_P1966608	LOC640701
2295954	A_66_P106497	ENSMUST0000010
2276510	A_55_P2140286	ENSMUST0000010
2276743	A_51_P154485	Fcrla
2285899	A_55_P2058028	ENSMUST0000010
2299955	A_55_P2187217	ENSMUST0000010
2309508	A_66_P124806	Tlr4
2286312	A_55_P2097279	Lilrb3
2282902	A_55_P2186972	ENSMUST0000010
2291515	A_52_P42245	Klrb1a
2274816	A_55_P2011950	LOC435333
2269728	A_52_P15405	Blk
2287696	A_55_P2119377	Klri2
2264476	A_55_P2103948	ENSMUST0000010
2302088	A_55_P2068595	LOC100047095
2261784	A_52_P614207	LOC100047222
2281798	A_55_P2066578	Ifi204
2260279	A_55_P2025586	ENSMUST0000010
2276840	A_52_P408757	Fcgr3
2263287	A_51_P322640	Ccl24
2306096	A_51_P375146	Cd36
2266536	A_55_P2058023	ENSMUST0000010
2264295	A_55_P2187067	ENSMUST0000010
2262142	A_52_P63973	ENSMUST0000010
2287115	A_55_P2027731	Il1rl1
2291370	A_55_P2004526	Klra8
2258950	A_66_P112234	LOC676175
2285560	A_55_P2058040	ENSMUST0000010
2297182	A_66_P133367	ENSMUST0000010
2276960	A_51_P245989	Ccr2
2301307	A_55_P1997756	Il6
2265415	A_52_P186262	ENSMUST0000010
2308625	A_55_P2051270	Tctex1d1
2289471	A_55_P1979364	ENSMUST0000010
2311225	A_55_P2186978	ENSMUST0000010
2286864	A_55_P2062802	ENSMUST0000010
2306362	A_55_P2130497	ENSMUST0000010
2279875	A_55_P2003915	ENSMUST0000010
2281868	A_51_P513776	LOC640979
2260889	A_55_P1957459	Lilrb4
2257552	A_55_P1962400	Il1rn
2300373	A_55_P2276221	Gm5222
2290157	A_51_P288295	LOC677484
2257556	A_55_P2155560	LOC630565
2295024	A_55_P2060897	ENSMUST0000010
2308773	A_51_P405638	LOC100045791
2289484	A_52_P374653	ENSMUST0000010
2265453	A_51_P500882	Ncr1
2273411	A_66_P116173	Il23r

2295994	A_51_P241457	Lilrb4
2299041	A_52_P630429	ENSMUST0000010
2302866	A_55_P1974577	Nfam1
2278427	A_66_P109731	ENSMUST0000010
2270408	A_52_P546162	ENSMUST0000010
2259200	A_52_P30641	ENSMUST0000010
2309991	A_55_P2140348	Igh-VJ558
2275786	A_55_P2008437	Cdkn2a
2288305	A_55_P2079079	Cd19
2289185	A_55_P2117342	ENSMUST0000010
2286378	A_52_P578436	ENSMUST0000010
2285307	A_55_P1988183	Cdkn2b
2301163	A_55_P2003911	ENSMUST0000010
2295039	A_55_P2026258	ENSMUST0000010
2271312	A_52_P15966	ENSMUST0000010
2273241	A_55_P2199202	Il22
2298904	A_51_P150710	Igj
2307634	A_55_P2167347	ENSMUST0000010
2297923	A_55_P2180839	Il13
2291437	A_66_P140474	LOC100047316
2311645	A_55_P2094104	LOC100047070
2305911	A_55_P1987406	ENSMUST0000010
2287230	A_66_P113466	ENSMUST0000010
2270417	A_55_P2057283	ENSMUST0000010
2295017	A_51_P461665	Cxcl9
2258981	A_66_P127412	ENSMUST0000010
2260024	A_55_P2029319	Cd70
2285366	A_55_P1978994	ENSMUST0000010
2283726	A_52_P232813	Cxcl3
2267745	A_55_P2004541	Klra7
2279870	A_55_P2098697	Tnfaip2
2303775	A_55_P2036693	Cd40
2261055	A_55_P2030299	LOC544904
2272642	A_55_P2004527	Klra8
2302806	A_55_P2134277	Igh-2
2264069	A_55_P2145224	ENSMUST0000010
2272896	A_55_P1974014	LOC100046496
2307552	A_51_P117581	Cables1
2301716	A_55_P2087622	Il4i1
2261533	A_51_P114462	Ccl17
2278204	A_55_P1985985	Il3
2270460	A_51_P185660	Ccl9
2261676	A_51_P520966	Icosl
2270800	A_51_P242930	Lat2
2282732	A_55_P1971174	Cd1d2
2271993	A_51_P390937	LOC675735
2279139	A_55_P2065510	ENSMUST0000010
2298039	A_51_P470079	Il1r2
2268109	A_55_P2214531	Il9r
2303110	A_52_P68893	Ifng
2260776	A_66_P134171	ENSMUST0000010
2301510	A_51_P500090	Klra7
2305866	A_51_P333274	Gzmb
2309745	A_52_P638459	Ccl5
2310147	A_51_P284608	Cd74
2293209	A_51_P327796	Itgb5

2276850	A_51_P104768	Gm16697
2264647	A_55_P1959421	ENSMUST0000010
2297153	A_55_P2016462	Cxcl10
2272049	A_51_P109570	Klrb1b
2291885	A_55_P1977038	Cxcl9
2271948	A_55_P1964325	Klrb1b
2290350	A_51_P205334	Tnfrsf8
2261417	A_55_P1999033	LOC100046275
2277497	A_51_P405476	Fcer1g
2311424	A_55_P2067620	ENSMUST0000010
2288611	A_51_P196925	Cx3cl1
2262297	A_66_P120987	Cd79a
2260255	A_52_P99888	Cxcl16
2275538	A_51_P346668	Irf5
2286550	A_55_P1975560	Ifi204
2305609	A_51_P342652	Cd79b
2278627	A_55_P2032718	Klra9
2282597	A_55_P2132532	ENSMUST0000010
2294364	A_55_P2009375	Cdk18
2296320	A_55_P2004652	Klrc1
2288151	A_55_P2081164	Cd300lf
2294087	A_55_P2117614	Tnfrsf13c
2275179	A_51_P150302	Crtam
2279835	A_51_P271503	Il1r1
2264890	A_55_P2129047	Tlr7
2306283	A_55_P2137496	LOC100047815
2296282	A_55_P1970755	Hdac9
2306440	A_55_P2106121	Il21
2260286	A_55_P2014427	Il17re
2301794	A_55_P1992592	Il5ra
2309812	A_51_P234956	Xcl1
2305468	A_55_P2132533	ENSMUST0000010
2284569	A_52_P517098	Il18rap
2298628	A_55_P2157829	ENSMUST0000010
2299483	A_55_P2100475	Gas2l3
2302450	A_55_P2063755	Naip6
2289283	A_51_P378298	Faim3
2307398	A_55_P1984168	Casp4
2262852	A_55_P2027737	Il1rl1
2308900	A_51_P237865	Il4

Description	ANOVA p-value
Mus musculus chemokine (C-C motif) ligand 6 (Ccl6), mRNA [NM_009139]	3,92E-04
Mus musculus complement factor B (Cfb), transcript variant 1, mRNA [NM_00819	3,12E-04
PREDICTED: Mus musculus immunoglobulin heavy chain (J558 family) (Igh-VJ55	1,11E-16
PREDICTED: Mus musculus immunoglobulin heavy chain (J558 family) (Igh-VJ55	4,37E-17
Mus musculus immunoglobulin heavy chain complex, mRNA (cDNA clone MGC:3	1,89E-14
immunoglobulin kappa chain variable 19-93 [Source:MGI Symbol;Acc:MGI:10761	3,95E-09
immunoglobulin heavy variable V12-3 [Source:MGI Symbol;Acc:MGI:3646760] [E	6,44E-04
Mus musculus granzyme A (Gzma), mRNA [NM_010370]	6,70E-04
Mus musculus killer cell lectin-like receptor subfamily A, member 23 (Klra23), mRN	1,05E-03
Mus musculus growth arrest specific 7 (Gas7), transcript variant 2, mRNA [NM_00	2,27E-06
immunoglobulin kappa variable 10-94 [Source:MGI Symbol;Acc:MGI:3646140] [E	7,44E-08
Mus musculus killer cell lectin-like receptor subfamily B member 1C (Klrb1c), trans	2,72E-12
immunoglobulin kappa variable 3-9 [Source:MGI Symbol;Acc:MGI:1330856] [ENS	9,43E-07
immunoglobulin kappa variable 3-12 [Source:MGI Symbol;Acc:MGI:1330815] [EN	3,96E-08
immunoglobulin kappa variable 6-15 [Source:MGI Symbol;Acc:MGI:1330831] [EN	1,17E-10
Mus musculus CD1d2 antigen (Cd1d2), mRNA [NM_007640]	7,44E-10
Mus musculus C-type lectin domain family 4, member e (Clec4e), mRNA [NM_019	1,80E-02
M.musculus mRNA for C2-Fcgamma fusion protein [X96776]	2,56E-10
Mus musculus killer cell lectin-like receptor family E member 1 (Klre1), mRNA [NM_	1,50E-08
Mus musculus killer cell lectin-like receptor subfamily K, member 1 (Klrk1), transcri	1,47E-08
Mus musculus killer cell lectin-like receptor, subfamily A, member 5 (Klra5), mRNA	3,56E-03
M.musculus immunoglobulin VDJ region (A8H). [X80960]	1,42E-08
Mus musculus killer cell lectin-like receptor subfamily A, member 23 (Klra23), mRN	8,35E-03
immunoglobulin heavy variable V1-54 [Source:MGI Symbol;Acc:MGI:3647133] [E	4,61E-07
immunoglobulin kappa variable 3-4 [Source:MGI Symbol;Acc:MGI:1330855] [ENS	4,63E-08
Mus musculus interleukin 1 beta (Il1b), mRNA [NM_008361]	2,60E-03
Mus musculus Fc receptor, IgG, low affinity IIb (Fcgr2b), transcript variant 1, mRN	2,71E-07
immunoglobulin kappa chain variable 4-91 [Source:MGI Symbol;Acc:MGI:364227	1,82E-09
Mus musculus paired-Ig-like receptor A7 (Pira7), mRNA [NM_011094]	1,46E-04
immunoglobulin kappa variable 4-80 [Source:MGI Symbol;Acc:MGI:4439653] [EN	7,78E-13
Mus musculus transforming growth factor, beta induced (Tgfbi), mRNA [NM_0093	4,72E-04
Mus musculus interleukin 5 (Il5), mRNA [NM_010558]	1,71E-08
Mus musculus killer cell lectin-like receptor, subfamily A, member 4 (Klra4), transc	1,00E-03
Mus musculus granzyme C (Gzmc), mRNA [NM_010371]	7,89E-06
immunoglobulin kappa variable 1-135 [Source:MGI Symbol;Acc:MGI:3819952] [E	1,99E-08
immunoglobulin kappa variable 1-133 [Source:MGI Symbol;Acc:MGI:3648380] [E	1,33E-11
Mus musculus immunoresponsive gene 1 (Irg1), mRNA [NM_008392]	1,27E-09
Mus musculus integrin alpha X (Itgax), mRNA [NM_021334]	8,42E-05
Mus musculus chemokine (C-C motif) ligand 19 (Ccl19), mRNA [NM_011888]	5,22E-03
immunoglobulin heavy variable V1-23 [Source:MGI Symbol;Acc:MGI:3815050] [E	6,33E-10
immunoglobulin kappa variable 3-4 [Source:MGI Symbol;Acc:MGI:1330855] [ENS	2,24E-05
Mus musculus interleukin 9 (Il9), mRNA [NM_008373]	4,74E-12
Mus musculus G1 to S phase transition 2 (Gspt2), mRNA [NM_008179]	7,38E-03
immunoglobulin kappa variable 1-132 [Source:MGI Symbol;Acc:MGI:3648800] [E	8,05E-12
Mus musculus leucine-rich repeat kinase 1 (Lrrk1), mRNA [NM_146191]	1,38E-04
immunoglobulin heavy variable V8-5 [Source:MGI Symbol;Acc:MGI:3645478] [EN	8,29E-08

immunoglobulin kappa chain variable 1-122 [Source:MGI Symbol;Acc:MGI:44397	2,24E-11
Mus musculus complement component 1, r subcomponent A (C1ra), mRNA [NM_	2,64E-05
Mus musculus lymphotoxin B receptor (Ltbr), mRNA [NM_010736]	7,13E-05
immunoglobulin kappa variable 3-10 [Source:MGI Symbol;Acc:MGI:1330821] [EN	1,95E-06
immunoglobulin kappa variable 1-110 [Source:MGI Symbol;Acc:MGI:4439558] [E	1,46E-10
immunoglobulin kappa variable 6-32 [Source:MGI Symbol;Acc:MGI:3641634] [EN	7,97E-11
Mus musculus cyclin-dependent kinase inhibitor 2B (p15, inhibits CDK4) (Cdkn2b	5,27E-03
PREDICTED: Mus musculus similar to immunoglobulin mu-chain-like (LOC100505	4,84E-11
immunoglobulin kappa chain variable 8-30 [Source:MGI Symbol;Acc:MGI:364225	3,68E-11
immunoglobulin kappa variable 1-131 [Source:MGI Symbol;Acc:MGI:3645551] [E	1,90E-11
Mus musculus Fc receptor-like A (Fcrla), transcript variant 2, mRNA [NM_145141]	3,68E-08
immunoglobulin kappa variable 4-63 [Source:MGI Symbol;Acc:MGI:3645235] [EN	5,78E-12
immunoglobulin heavy variable V1-42 [Source:MGI Symbol;Acc:MGI:3704123] [E	4,88E-09
Mus musculus toll-like receptor 4 (Tlr4), mRNA [NM_021297]	3,71E-03
Mus musculus leukocyte immunoglobulin-like receptor, subfamily B (with TM and I	5,65E-04
Mus musculus immunoglobulin kappa light chain, variable region mRNA, clone BA	2,07E-10
Mus musculus killer cell lectin-like receptor subfamily B member 1A (Klrb1a), trans	1,21E-10
immunoglobulin heavy variable 1-85 [Source:MGI Symbol;Acc:MGI:3645723] [EN	3,78E-11
Mus musculus B lymphoid kinase (Blk), mRNA [NM_007549]	8,13E-07
Mus musculus killer cell lectin-like receptor family I member 2 (Klri2), mRNA [NM_1	3,38E-05
immunoglobulin kappa variable 4-53 [Source:MGI Symbol;Acc:MGI:2686266] [EN	1,21E-11
Mus musculus predicted gene, 18756 (Gm18756), transcript variant 2, non-codin	1,39E-02
immunoglobulin kappa variable 3-7 [Source:MGI Symbol;Acc:MGI:1330852] [ENS	1,02E-06
Mus musculus interferon activated gene 204, mRNA (cDNA clone MGC:18551 IM	4,84E-04
immunoglobulin heavy variable V3-8 [Source:MGI Symbol;Acc:MGI:3645298] [EN	5,42E-09
Mus musculus Fc receptor, IgG, low affinity III (Fcgr3), mRNA [NM_010188]	2,89E-04
Mus musculus chemokine (C-C motif) ligand 24 (Ccl24), mRNA [NM_019577]	6,50E-14
Mus musculus CD36 antigen (Cd36), transcript variant 2, mRNA [NM_007643]	5,05E-05
immunoglobulin kappa variable 4-57-1 [Source:MGI Symbol;Acc:MGI:2686264] [E	1,55E-12
immunoglobulin kappa variable 1-117 [Source:MGI Symbol;Acc:MGI:4439721] [E	2,38E-11
immunoglobulin kappa chain variable 1-35 [Source:MGI Symbol;Acc:MGI:443961	3,20E-10
Mus musculus interleukin 1 receptor-like 1 (Il1rl1), transcript variant 1, mRNA [NM_	4,30E-03
Mus musculus killer cell lectin-like receptor, subfamily A, member 8 (Klra8), transcr	1,08E-03
Mus musculus isolate C5-12 anti-GBM immunoglobulin kappa chain variable regio	3,80E-07
immunoglobulin kappa variable 4-73 [Source:MGI Symbol;Acc:MGI:3645825] [EN	4,44E-12
immunoglobulin kappa chain variable 8-27 [Source:MGI Symbol;Acc:MGI:443986	4,21E-08
Mus musculus chemokine (C-C motif) receptor 2 (Ccr2), mRNA [NM_009915]	1,01E-11
Mus musculus interleukin 6 (Il6), mRNA [NM_031168]	2,48E-05
immunoglobulin kappa chain variable 6-25 [Source:MGI Symbol;Acc:MGI:443986	2,00E-13
Mus musculus Tctex1 domain containing 1 (Tctex1d1), transcript variant 1, mRNA	1,52E-03
immunoglobulin heavy variable 1-83 [Source:MGI Symbol;Acc:MGI:3648939] [EN	4,72E-09
immunoglobulin kappa variable 4-78 [Source:MGI Symbol;Acc:MGI:3819775] [EN	5,90E-13
Mus musculus partial mRNA for immunoglobulin kappa light chain variable region	2,57E-10
immunoglobulin heavy variable V1-67 [Source:MGI Symbol;Acc:MGI:3645228] [E	1,13E-12
immunoglobulin kappa variable 6-13 [Source:MGI Symbol;Acc:MGI:1330829] [EN	1,04E-09
Immunoglobulin heavy chain V gene segment [Source:IMGT/GENE-DB;Acc:IGHV	2,08E-12
Mus musculus leukocyte immunoglobulin-like receptor, subfamily B, member 4 (Li	3,42E-06
Mus musculus interleukin 1 receptor antagonist (Il1rn), transcript variant 2, mRNA	1,73E-09
immunoglobulin kappa variable 17-121 [Source:MGI Symbol;Acc:MGI:3647671] [	7,90E-10
immunoglobulin heavy variable V1-7 [Source:MGI Symbol;Acc:MGI:3704122] [EN	3,01E-12
immunoglobulin heavy variable V1-5 [Source:MGI Symbol;Acc:MGI:3704121] [EN	2,05E-10
immunoglobulin kappa constant [Source:MGI Symbol;Acc:MGI:96495] [ENSMUS	1,24E-12
immunoglobulin heavy variable V9-3 [Source:MGI Symbol;Acc:MGI:3642720] [EN	2,79E-08
immunoglobulin kappa variable 3-10 [Source:MGI Symbol;Acc:MGI:1330821] [EN	1,89E-05
Mus musculus natural cytotoxicity triggering receptor 1 (Ncr1), mRNA [NM_01074	3,16E-05
Mus musculus interleukin 23 receptor (Il23r), mRNA [NM_144548]	1,01E-05

Mus musculus leukocyte immunoglobulin-like receptor, subfamily B, member 4 (Li	**9,57E-07**
immunoglobulin kappa variable 2-116 [Source:MGI Symbol;Acc:MGI:3647785] [E	**2,86E-11**
Mus musculus Nfat activating molecule with ITAM motif 1 (Nfam1), mRNA [NM_02	**2,47E-09**
immunoglobulin kappa chain variable 9-124 [Source:MGI Symbol;Acc:MGI:36468	**1,38E-07**
immunoglobulin kappa variable 4-58 [Source:MGI Symbol;Acc:MGI:2685923] [EN	**2,53E-13**
immunoglobulin kappa variable 4-86 [Source:MGI Symbol;Acc:MGI:2685305] [EN	**5,59E-10**
Mus musculus immunoglobulin heavy chain complex, mRNA (cDNA clone MGC:1	**9,82E-10**
Mus musculus cyclin-dependent kinase inhibitor 2A (Cdkn2a), transcript variant 1	**7,68E-07**
Mus musculus CD19 antigen (Cd19), mRNA [NM_009844]	**3,47E-04**
immunoglobulin kappa variable 2-116 [Source:MGI Symbol;Acc:MGI:3647785] [E	**1,28E-11**
immunoglobulin kappa chain variable 4-71 [Source:MGI Symbol;Acc:MGI:443965	**8,21E-13**
Mus musculus cyclin-dependent kinase inhibitor 2B (p15, inhibits CDK4) (Cdkn2b	**4,35E-06**
immunoglobulin kappa variable 6-17 [Source:MGI Symbol;Acc:MGI:1330833] [EN	**7,25E-11**
immunoglobulin kappa variable 12-46 [Source:MGI Symbol;Acc:MGI:4439773] [E	**6,58E-08**
immunoglobulin heavy variable 1-84 [Source:MGI Symbol;Acc:MGI:3644235] [EN	**7,02E-10**
Mus musculus interleukin 22 (Il22), mRNA [NM_016971]	**5,61E-07**
Mus musculus immunoglobulin joining chain (Igj), mRNA [NM_152839]	**5,03E-14**
immunoglobulin kappa variable 14-126 [Source:MGI Symbol;Acc:MGI:3643131] [	**4,32E-07**
Mus musculus interleukin 13 (Il13), mRNA [NM_008355]	**3,08E-14**
immunoglobulin kappa chain variable 4-70 [Source:MGI Symbol;Acc:MGI:268634	**6,13E-13**
immunoglobulin kappa variable 8-28 [Source:MGI Symbol;Acc:MGI:3642251] [EN	**3,50E-10**
immunoglobulin lambda variable 1 [Source:MGI Symbol;Acc:MGI:96530] [ENSMU	**1,02E-11**
immunoglobulin kappa chain variable 4-90 [Source:MGI Symbol;Acc:MGI:443983	**3,12E-10**
immunoglobulin lambda constant 2 [Source:MGI Symbol;Acc:MGI:99547] [ENSM	**3,54E-11**
Mus musculus chemokine (C-X-C motif) ligand 9 (Cxcl9), mRNA [NM_008599]	**9,38E-12**
immunoglobulin heavy variable V11-2 [Source:MGI Symbol;Acc:MGI:4947968] [E	**8,84E-07**
Mus musculus CD70 antigen (Cd70), mRNA [NM_011617]	**4,43E-09**
immunoglobulin heavy variable V9-2 [Source:MGI Symbol;Acc:MGI:3643816] [EN	**1,38E-07**
Mus musculus chemokine (C-X-C motif) ligand 3 (Cxcl3), mRNA [NM_203320]	**1,25E-07**
Mus musculus killer cell lectin-like receptor, subfamily A, member 7 (Klra7), transcr	**7,44E-03**
Mus musculus tumor necrosis factor, alpha-induced protein 2 (Tnfaip2), mRNA [N	**1,85E-07**
Mus musculus CD40 antigen (Cd40), transcript variant 1, mRNA [NM_011611]	**7,96E-08**
immunoglobulin heavy variable V14-3 [Source:MGI Symbol;Acc:MGI:4439764] [E	**1,50E-11**
Mus musculus killer cell lectin-like receptor, subfamily A, member 8 (Klra8), transcr	**1,03E-03**
immunoglobulin heavy variable V1-18 [Source:MGI Symbol;Acc:MGI:4439780] [E	**9,41E-10**
immunoglobulin lambda constant 3 [Source:MGI Symbol;Acc:MGI:99886] [ENSM	**3,06E-11**
immunoglobulin kappa variable 2-109 [Source:MGI Symbol;Acc:MGI:3642626] [E	**2,07E-10**
Mus musculus CDK5 and Abl enzyme substrate 1 (Cables1), transcript variant 2,	**1,45E-09**
Mus musculus interleukin 4 induced 1 (Il4i1), mRNA [NM_010215]	**2,05E-10**
Mus musculus chemokine (C-C motif) ligand 17 (Ccl17), mRNA [NM_011332]	**3,43E-11**
Mus musculus interleukin 3 (Il3), mRNA [NM_010556]	**6,10E-10**
Mus musculus chemokine (C-C motif) ligand 9 (Ccl9), mRNA [NM_011338]	**3,27E-03**
Mus musculus icos ligand (Icosl), mRNA [NM_015790]	**3,57E-13**
Mus musculus linker for activation of T cells family, member 2 (Lat2), transcript va	**1,04E-03**
Mus musculus CD1d2 antigen (Cd1d2), mRNA [NM_007640]	**1,20E-10**
immunoglobulin kappa variable 14-111 [Source:MGI Symbol;Acc:MGI:4439863] [	**3,41E-10**
immunoglobulin heavy variable V1-20 [Source:MGI Symbol;Acc:MGI:3644607] [E	**4,55E-11**
Mus musculus interleukin 1 receptor, type II (Il1r2), mRNA [NM_010555]	**8,06E-08**
Mus musculus interleukin 9 receptor (Il9r), transcript variant 1, mRNA [NM_00113	**2,32E-06**
Mus musculus interferon gamma (Ifng), mRNA [NM_008337]	**2,69E-04**
immunoglobulin kappa chain variable 8-24 [Source:MGI Symbol;Acc:MGI:494795	**1,56E-10**
Mus musculus killer cell lectin-like receptor, subfamily A, member 7 (Klra7), transcr	**1,20E-02**
Mus musculus granzyme B (Gzmb), mRNA [NM_013542]	**4,84E-09**
Mus musculus chemokine (C-C motif) ligand 5 (Ccl5), mRNA [NM_013653]	**8,56E-13**
Mus musculus CD74 antigen (invariant polypeptide of major histocompatibility cor	**7,57E-07**
Mus musculus integrin beta 5 (Itgb5), transcript variant 2, mRNA [NM_010580]	**2,23E-08**

Immunoglobulin heavy chain V gene segment [Source:IMGT/GENE-DB;Acc:IGHV	3,11E-07
immunoglobulin kappa chain variable 6-29 [Source:MGI Symbol;Acc:MGI:443961	5,81E-09
Mus musculus chemokine (C-X-C motif) ligand 10 (Cxcl10), mRNA [NM_021274]	2,83E-06
Mus musculus killer cell lectin-like receptor subfamily B member 1B (Klrb1b), mRN	2,55E-04
Mus musculus chemokine (C-X-C motif) ligand 9 (Cxcl9), mRNA [NM_008599]	4,55E-11
Mus musculus killer cell lectin-like receptor subfamily B member 1B (Klrb1b), mRN	8,94E-06
Mus musculus tumor necrosis factor receptor superfamily, member 8 (Tnfrsf8), mR	1,11E-09
immunoglobulin heavy variable V8-12 [Source:MGI Symbol;Acc:MGI:3642873] [E	1,11E-07
Mus musculus Fc receptor, IgE, high affinity I, gamma polypeptide (Fcer1g), mRN	2,78E-04
immunoglobulin kappa chain variable 2-137 [Source:MGI Symbol;Acc:MGI:44398	6,92E-08
Mus musculus chemokine (C-X3-C motif) ligand 1 (Cx3cl1), mRNA [NM_009142]	4,44E-05
Mus musculus CD79A antigen (immunoglobulin-associated alpha) (Cd79a), mRN	1,50E-07
Mus musculus chemokine (C-X-C motif) ligand 16 (Cxcl16), mRNA [NM_023158]	7,75E-04
Mus musculus interferon regulatory factor 5 (Irf5), transcript variant 2, mRNA [NM_	5,45E-05
Mus musculus interferon activated gene 204 (Ifi204), mRNA [NM_008329]	1,13E-10
Mus musculus CD79B antigen (Cd79b), mRNA [NM_008339]	6,00E-09
Mus musculus killer cell lectin-like receptor subfamily A, member 9 (Klra9), mRNA [	5,09E-03
immunoglobulin kappa chain variable 15-103 [Source:MGI Symbol;Acc:MGI:9651	2,42E-09
Mus musculus cyclin-dependent kinase 18 (Cdk18), mRNA [NM_008795]	1,60E-03
Mus musculus killer cell lectin-like receptor subfamily C, member 1 (Klrc1), transcri	3,44E-04
Mus musculus CD300 antigen like family member F (Cd300lf), transcript variant 1,	5,45E-03
Mus musculus tumor necrosis factor receptor superfamily, member 13c (Tnfrsf13c	1,50E-04
Mus musculus cytotoxic and regulatory T cell molecule (Crtam), mRNA [NM_0194	4,03E-09
Mus musculus interleukin 1 receptor, type I (Il1r1), transcript variant 1, mRNA [NM	2,03E-05
Mus musculus toll-like receptor 7 (Tlr7), mRNA [NM_133211]	3,01E-10
Mus musculus CD79A antigen (immunoglobulin-associated alpha), mRNA (cDNA	1,06E-08
Mus musculus histone deacetylase 9 (Hdac9), mRNA [NM_024124]	1,57E-06
Mus musculus interleukin 21 (Il21), mRNA [NM_021782]	7,52E-07
Mus musculus interleukin 17 receptor E (Il17re), transcript variant 3, mRNA [NM_(	1,59E-08
Mus musculus interleukin 5 receptor, alpha (Il5ra), mRNA [NM_008370]	1,42E-02
Mus musculus chemokine (C motif) ligand 1 (Xcl1), mRNA [NM_008510]	1,30E-03
immunoglobulin kappa chain variable 15-103 [Source:MGI Symbol;Acc:MGI:9651	1,40E-09
Mus musculus interleukin 18 receptor accessory protein (Il18rap), mRNA [NM_01(	1,47E-07
chemokine (C-C motif) ligand 1 [Source:MGI Symbol;Acc:MGI:98258] [ENSMUST	8,64E-13
Mus musculus growth arrest-specific 2 like 3 (Gas2l3), transcript variant 2, mRNA	8,20E-07
Mus musculus NLR family, apoptosis inhibitory protein 6 (Naip6), mRNA [NM_010	5,25E-10
Mus musculus Fas apoptotic inhibitory molecule 3 (Faim3), mRNA [NM_026976]	1,75E-09
Mus musculus caspase 4, apoptosis-related cysteine peptidase (Casp4), mRNA [	1,62E-08
Mus musculus interleukin 1 receptor-like 1 (Il1rl1), transcript variant 1, mRNA [NM_	4,12E-04
Mus musculus interleukin 4 (Il4), mRNA [NM_021283]	7,07E-12

Adjusted ANOVA p-value	Tukey p-value	Fold change	Log2 ratio	Median	wt_non-stim_1	wt_non-stim_2	wt_non-stim_3	wt_non-stim_4	wt_non-stim_21
1,28E-03	1,05E-03	20,620	4,366	6,389	-0,298	-0,600	-1,745	-0,461	3,048
1,04E-03	7,90E-03	14,291	3,837	3,183	-1,075	-1,129	-1,137	-0,984	-1,134
3,47E-14	4,90E-10	13,929	3,800	14,844	2,474	1,498	2,306	2,205	2,251
1,94E-14	7,47E-10	12,702	3,667	14,047	2,644	1,617	2,475	2,367	2,430
1,36E-12	5,05E-07	11,424	3,514	10,262	3,018	1,568	2,828	2,732	2,669
3,54E-08	1,09E-02	10,808	3,434	9,436	1,654	1,455	2,844	1,827	0,890
2,04E-03	3,80E-02	10,418	3,381	5,908	-0,295	-0,562	4,328	0,321	0,860
2,11E-03	3,08E-03	9,448	3,240	10,766	-1,262	-0,978	-1,422	-1,262	2,422
3,19E-03	1,30E-03	9,057	3,179	4,685	-1,067	-1,043	-0,546	-0,735	2,239
1,11E-05	1,66E-05	8,877	3,150	4,763	-0,555	-0,693	-0,083	-0,588	0,710
4,93E-07	2,29E-03	8,766	3,132	8,507	0,612	0,255	1,575	0,578	1,555
6,87E-11	8,66E-06	8,682	3,118	9,508	2,156	2,317	2,076	1,725	1,877
4,97E-06	1,09E-02	8,282	3,050	5,732	2,059	0,078	1,207	1,550	1,056
2,77E-07	2,07E-03	8,140	3,025	8,850	1,003	0,747	1,895	1,048	0,294
1,63E-09	2,23E-04	7,912	2,984	9,445	1,843	1,167	1,892	1,830	0,853
8,13E-09	8,66E-06	7,121	2,832	4,036	-0,812	-2,069	-0,196	-2,017	-1,611
4,30E-02	1,66E-02	6,845	2,775	2,606	-0,808	-0,167	0,072	0,083	-0,072
3,21E-09	6,70E-05	6,543	2,710	7,959	1,815	1,930	1,780	0,808	1,914
1,16E-07	1,11E-04	6,476	2,695	6,158	0,378	0,525	0,516	0,044	1,862
1,14E-07	4,38E-05	6,426	2,684	5,445	0,596	0,478	0,513	0,157	0,548
9,82E-03	5,80E-03	6,382	2,674	3,763	-1,934	-0,723	-1,757	-0,574	2,070
1,11E-07	6,47E-03	6,084	2,605	6,333	1,808	1,056	1,605	2,385	0,645
2,14E-02	9,87E-03	6,017	2,589	4,873	-0,285	-1,547	-0,354	-0,353	2,737
2,59E-06	2,18E-02	5,959	2,575	7,436	1,451	0,518	1,657	0,112	-0,112
3,19E-07	1,92E-03	5,784	2,532	8,962	1,404	1,058	2,934	1,317	0,113
7,35E-03	1,71E-03	5,720	2,516	4,825	0,351	-0,047	-1,759	0,299	-1,104
1,59E-06	1,97E-05	5,665	2,502	5,301	-0,075	0,010	-0,055	-0,010	1,178
1,78E-08	5,65E-05	5,626	2,492	7,865	0,146	1,239	1,601	0,932	0,417
5,18E-04	1,23E-03	5,594	2,484	5,454	-0,225	0,118	-0,880	-0,951	1,899
2,52E-11	7,96E-06	5,579	2,480	10,602	2,233	1,163	2,355	1,604	1,306
1,52E-03	9,59E-03	5,487	2,456	6,004	-0,087	-0,021	-0,437	-0,220	2,963
1,31E-07	2,36E-04	5,374	2,426	4,255	-1,795	-2,075	-2,089	-2,002	-2,090
3,07E-03	1,70E-02	5,329	2,414	5,892	0,115	-0,719	-0,167	-0,115	2,994
3,49E-05	1,40E-05	5,315	2,410	8,294	0,514	0,383	0,563	0,651	0,574
1,50E-07	3,15E-04	5,234	2,388	9,184	1,829	1,149	1,662	1,217	0,541
2,54E-10	1,44E-05	5,144	2,363	11,313	1,917	1,446	2,011	1,612	0,864
1,30E-08	6,49E-06	5,067	2,341	4,981	-1,758	-1,099	-1,830	-1,410	-1,158
3,11E-04	4,40E-03	5,032	2,331	6,721	-0,015	0,015	-0,305	-0,442	2,274
1,39E-02	7,73E-03	5,007	2,324	2,644	0,597	0,599	-0,878	-0,295	0,038
7,07E-09	1,07E-02	4,942	2,305	5,936	1,318	2,745	2,574	0,959	1,893
9,12E-05	2,59E-02	4,908	2,295	9,978	0,984	0,505	2,970	1,242	-0,505
1,08E-10	4,26E-04	4,901	2,293	4,211	-2,074	-2,084	-2,062	-2,119	-1,614
1,91E-02	3,87E-02	4,857	2,280	3,058	-0,372	-1,085	-1,070	-1,078	0,424
1,68E-10	1,88E-05	4,740	2,245	9,109	1,882	1,388	2,035	1,635	0,707
4,90E-04	8,68E-04	4,727	2,241	6,279	-0,544	-1,644	-1,292	-1,040	0,703
5,44E-07	1,95E-02	4,617	2,207	7,457	2,817	1,020	1,779	2,278	0,789

3,98E-10	4,48E-05	4,528	2,179	11,475	1,939	1,551	2,232	1,663	0,846
1,06E-04	4,19E-05	4,528	2,179	3,475	-0,409	-0,427	-0,655	-1,267	-0,313
2,66E-04	1,33E-04	4,525	2,178	5,196	-0,961	-1,473	-1,020	-1,147	0,193
9,66E-06	3,77E-02	4,491	2,167	8,680	2,238	0,674	4,082	1,600	0,077
1,98E-09	2,59E-04	4,426	2,146	10,154	1,522	1,333	2,139	1,621	0,851
1,18E-09	3,88E-04	4,423	2,145	8,699	1,668	1,145	2,032	1,818	1,723
1,40E-02	2,42E-02	4,377	2,130	4,247	0,022	-2,247	1,329	-1,084	-1,119
7,66E-10	9,31E-04	4,377	2,130	9,478	1,483	1,406	1,769	1,471	1,403
6,07E-10	2,02E-04	4,368	2,127	10,696	1,407	1,030	1,798	1,835	1,047
3,46E-10	2,96E-05	4,362	2,125	5,329	1,522	0,824	1,520	1,627	0,526
2,59E-07	2,05E-04	4,347	2,120	7,533	0,378	-0,006	0,286	0,006	0,799
1,26E-10	3,27E-04	4,323	2,112	11,099	2,184	1,467	2,889	1,880	1,631
4,26E-08	3,31E-02	4,323	2,112	5,609	1,128	2,675	1,410	2,929	1,565
1,02E-02	3,57E-03	4,272	2,095	4,178	-1,311	-1,541	0,022	-0,229	-0,022
1,80E-03	1,19E-03	4,257	2,090	6,813	-0,386	-0,799	-1,164	-1,205	1,409
2,68E-09	9,75E-04	4,243	2,085	9,394	2,157	1,225	3,120	1,991	1,638
1,68E-09	6,00E-04	4,211	2,074	8,802	1,396	1,839	1,510	1,057	1,616
6,22E-10	7,41E-04	4,205	2,072	10,629	1,808	1,686	2,282	1,798	1,395
4,34E-06	5,17E-04	4,179	2,063	7,014	0,624	0,017	0,203	-0,162	0,595
1,33E-04	4,14E-03	4,170	2,060	5,552	0,113	-0,291	0,962	-0,113	1,451
2,35E-10	9,98E-04	4,158	2,056	9,993	2,344	1,346	2,875	1,922	1,496
3,41E-02	4,50E-02	4,150	2,053	2,031	-0,003	-0,015	-0,008	0,009	-0,020
5,35E-06	2,19E-02	4,144	2,051	10,743	0,899	0,296	2,736	0,819	-0,296
1,56E-03	1,97E-03	4,144	2,051	4,098	-0,202	-1,672	-0,525	-2,217	-0,146
4,68E-08	8,17E-03	4,138	2,049	8,832	1,763	1,807	1,716	1,636	0,887
9,69E-04	4,93E-03	4,132	2,047	5,107	-0,180	-0,022	0,022	0,102	1,647
3,47E-12	2,66E-04	4,130	2,046	5,991	-4,097	-2,805	-3,409	-2,489	-3,626
1,94E-04	4,57E-04	4,112	2,040	3,908	-0,171	-0,819	-1,091	-0,054	-0,035
4,43E-11	6,05E-05	4,101	2,036	11,259	2,071	1,148	2,661	1,842	1,371
4,19E-10	6,55E-05	4,095	2,034	11,345	1,837	1,545	2,258	1,730	0,921
3,90E-09	7,49E-05	4,093	2,033	9,210	1,808	1,238	1,703	1,325	0,638
1,17E-02	6,50E-03	4,073	2,026	3,721	-1,819	0,301	-1,850	-1,831	0,140
3,28E-03	1,71E-02	4,061	2,022	6,411	-0,143	-0,451	0,143	-0,292	2,288
2,17E-06	2,95E-02	4,033	2,012	7,026	1,865	0,016	1,806	1,932	-0,016
1,02E-10	2,26E-04	4,031	2,011	11,829	2,192	1,362	2,767	1,908	1,399
2,93E-07	1,53E-03	4,017	2,006	8,341	2,202	1,739	1,893	1,447	0,644
2,03E-10	4,01E-06	3,997	1,999	5,974	0,792	0,800	0,824	0,911	1,092
1,00E-04	9,94E-03	3,975	1,991	4,394	-2,390	-1,936	-1,567	-2,416	-0,102
8,28E-12	1,17E-05	3,910	1,967	9,090	1,975	1,343	2,008	2,194	1,167
4,48E-03	4,72E-03	3,910	1,967	2,745	0,304	-0,831	-0,840	-0,868	0,599
4,14E-08	1,28E-03	3,893	1,961	9,069	2,016	1,506	2,266	2,084	1,216
2,00E-11	6,29E-05	3,891	1,960	11,519	2,107	1,315	2,746	1,867	1,439
3,22E-09	6,72E-04	3,883	1,957	9,358	1,482	1,689	1,729	1,469	0,958
3,41E-11	1,48E-04	3,872	1,953	9,697	1,345	1,539	2,148	1,650	1,314
1,09E-08	2,67E-03	3,861	1,949	6,521	2,057	0,600	0,891	2,141	0,328
5,56E-11	1,68E-04	3,792	1,923	11,027	1,781	1,666	2,225	1,882	1,384
1,62E-05	3,53E-05	3,789	1,922	9,050	-0,697	-0,384	-0,666	-1,017	-0,446
1,71E-08	6,01E-04	3,776	1,917	3,889	-1,779	-1,809	-1,810	-0,816	-1,813
8,57E-09	5,57E-04	3,774	1,916	8,917	2,401	1,132	1,814	1,153	0,813
7,45E-11	1,90E-04	3,753	1,908	11,234	1,779	1,619	2,246	1,711	1,363
2,66E-09	1,58E-03	3,753	1,908	7,887	1,858	1,712	2,138	1,919	1,555
3,68E-11	3,08E-06	3,704	1,889	16,491	1,060	0,946	1,197	0,953	0,700
2,02E-07	7,43E-03	3,681	1,880	8,565	1,359	1,864	1,294	0,694	0,354
7,79E-05	4,92E-02	3,668	1,875	8,648	2,205	0,703	4,076	1,632	0,130
1,25E-04	1,56E-02	3,650	1,868	4,678	-0,364	0,048	0,337	-0,330	2,421
4,38E-05	1,67E-05	3,650	1,868	6,478	-0,219	-0,120	0,119	-0,612	-0,809

**5,04E-06**	**2,59E-04**	**3,650**	1,868	6,723	-0,851	-1,387	-1,173	-2,056	-1,136
**4,90E-10**	**2,03E-04**	**3,615**	1,854	9,672	1,595	1,618	1,846	1,541	0,949
**2,33E-08**	**6,76E-05**	**3,610**	1,852	6,554	0,534	0,263	0,299	0,393	1,836
**8,62E-07**	**3,78E-02**	**3,603**	1,849	9,450	1,944	1,463	2,055	2,032	0,742
**1,00E-11**	**1,32E-04**	**3,600**	1,848	10,069	2,247	1,337	2,467	1,597	1,326
**6,34E-09**	**8,10E-03**	**3,585**	1,842	8,696	2,370	1,584	2,512	0,951	1,124
**1,04E-08**	**7,44E-03**	**3,565**	1,834	7,989	1,482	1,615	1,944	1,574	1,237
**4,12E-06**	**3,69E-05**	**3,563**	1,833	6,066	-0,733	-0,519	-0,886	-0,752	-0,453
**1,15E-03**	**1,33E-03**	**3,555**	1,830	8,944	-0,401	-1,145	-1,150	-1,302	0,198
**2,46E-10**	**6,11E-05**	**3,548**	1,827	10,323	1,644	1,472	1,769	1,521	0,911
**2,63E-11**	**1,02E-04**	**3,533**	1,821	12,686	2,199	1,363	2,673	1,814	1,466
**2,02E-05**	**2,22E-04**	**3,519**	1,815	5,613	-0,092	-0,438	-0,739	-0,838	-0,309
**1,09E-09**	**4,27E-04**	**3,509**	1,811	11,980	1,739	1,009	1,858	1,843	1,017
**4,40E-07**	**1,41E-02**	**3,506**	1,810	9,559	1,598	1,095	2,143	1,557	0,645
**7,74E-09**	**2,05E-03**	**3,504**	1,809	4,786	1,647	0,344	2,137	2,425	1,384
**3,08E-06**	**2,09E-05**	**3,497**	1,806	4,668	*-0,892*	*-1,037*	-0,348	-0,150	*-1,229*
**2,84E-12**	**4,62E-06**	**3,473**	1,796	14,290	1,638	1,428	1,966	1,554	1,156
**2,44E-06**	**2,90E-03**	**3,458**	1,790	12,375	0,695	0,054	0,116	0,061	0,341
**1,96E-12**	**9,84E-04**	**3,427**	1,777	6,878	*-3,864*	*-3,542*	*-3,336*	*-3,750*	-2,602
**2,06E-11**	**3,21E-04**	**3,378**	1,756	11,264	2,354	1,427	2,813	2,107	1,628
**4,21E-09**	**1,37E-03**	**3,371**	1,753	11,045	1,759	0,830	1,960	2,027	1,082
**2,04E-10**	**2,88E-04**	**3,366**	1,751	13,483	1,607	1,469	2,014	1,624	0,966
**3,82E-09**	**2,20E-03**	**3,343**	1,741	9,204	2,151	1,024	2,564	1,688	1,433
**5,88E-10**	**2,63E-04**	**3,324**	1,733	15,135	1,311	0,965	1,613	1,336	0,814
**1,91E-10**	**1,46E-03**	**3,315**	1,729	5,630	*-2,339*	*-3,742*	*-3,742*	*-2,207*	*-3,119*
**4,68E-06**	**1,93E-03**	**3,260**	1,705	8,654	0,961	-0,015	0,015	0,289	0,539
**3,91E-08**	**4,82E-06**	**3,256**	1,703	7,538	-1,048	-1,498	-1,010	-1,187	-0,977
**8,62E-07**	**2,59E-02**	**3,245**	1,698	8,999	1,975	1,978	1,697	0,804	0,522
**7,88E-07**	**4,62E-05**	**3,242**	1,697	2,307	*-0,103*	*-0,208*	*-0,194*	*-0,216*	*-0,211*
**1,93E-02**	**2,02E-02**	**3,229**	1,691	6,882	-0,386	-0,722	-0,580	-0,285	2,331
**1,13E-06**	**2,14E-02**	**3,220**	1,687	7,023	-0,652	-0,460	-0,079	0,214	0,638
**5,24E-07**	**1,99E-04**	**3,207**	1,681	6,139	-1,200	-1,741	-1,066	-1,507	-0,401
**2,83E-10**	**5,86E-04**	**3,187**	1,672	11,483	1,607	1,342	2,012	1,514	1,198
**3,15E-03**	**2,96E-02**	**3,167**	1,663	6,699	0,029	-0,327	0,047	-0,345	2,549
**9,99E-09**	**1,26E-02**	**3,154**	1,657	8,471	1,784	1,448	2,046	1,384	1,451
**5,18E-10**	**1,55E-04**	**3,147**	1,654	14,881	1,204	0,839	1,488	1,304	0,785
**2,68E-09**	**1,52E-03**	**3,123**	1,643	9,552	1,453	1,649	1,854	1,516	0,973
**1,46E-08**	**2,97E-06**	**3,082**	1,624	7,342	0,032	-0,032	-0,176	-0,429	0,505
**2,66E-09**	**4,56E-05**	**3,069**	1,618	10,181	-1,469	-1,621	-1,781	-1,672	-0,671
**5,73E-10**	**2,96E-04**	**3,067**	1,617	7,732	-1,859	-3,313	-2,411	-2,869	-1,418
**6,84E-09**	**2,72E-02**	**3,067**	1,617	7,686	-2,886	-3,371	*-3,946*	-3,509	*-4,923*
**9,08E-03**	**4,60E-02**	**3,029**	1,599	7,157	-2,761	-1,652	-1,971	-2,304	1,493
**1,32E-11**	**1,22E-05**	**2,998**	1,584	8,505	1,465	1,115	1,384	1,155	1,054
**3,17E-03**	**2,85E-03**	**2,983**	1,577	6,826	-0,771	-0,968	-0,573	-0,944	0,428
**1,67E-09**	**2,82E-04**	**2,965**	1,568	7,926	1,222	0,878	1,002	0,992	0,522
**4,12E-09**	**7,56E-04**	**2,910**	1,541	12,411	1,128	1,062	1,315	1,153	0,467
**7,26E-10**	**1,92E-03**	**2,880**	1,526	9,689	1,768	1,642	2,070	1,462	1,372
**5,30E-07**	**1,09E-04**	**2,834**	1,503	10,524	-0,960	-0,595	-1,368	-1,790	-1,231
**1,13E-05**	**2,10E-02**	**2,797**	1,484	4,715	0,710	0,279	0,600	0,353	0,947
**9,09E-04**	**1,44E-03**	**2,759**	1,464	14,060	-0,152	0,003	0,064	-0,160	-0,575
**2,10E-09**	**6,62E-04**	**2,747**	1,458	9,865	1,637	0,985	1,374	1,757	0,716
**2,99E-02**	**2,05E-02**	**2,742**	1,455	5,843	-0,378	-1,076	-0,247	0,018	2,531
**4,23E-08**	**3,22E-03**	**2,736**	1,452	10,847	-2,300	-2,208	-2,281	-2,266	-1,127
**2,71E-11**	**3,17E-04**	**2,726**	1,447	14,643	1,359	1,583	1,464	1,108	1,778
**4,06E-06**	**3,83E-05**	**2,721**	1,444	13,958	0,214	-0,165	-0,093	-0,237	0,469
**1,65E-07**	**1,03E-05**	**2,709**	1,438	7,084	0,202	-0,038	-0,043	-0,146	0,290

1,81E-06	2,28E-02	2,676	1,420	9,957	0,365	0,000	0,467	0,140	0,000
4,98E-08	3,58E-02	2,595	1,376	6,555	1,105	0,779	1,369	0,998	0,790
1,36E-05	6,75E-06	2,595	1,376	9,845	-0,244	-0,058	-0,868	-0,994	-0,760
8,62E-04	1,93E-03	2,583	1,369	5,544	0,059	-0,499	0,131	-0,615	0,674
7,26E-10	5,03E-04	2,578	1,366	5,063	*-1,601*	*-2,488*	*-3,275*	*-1,778*	*-2,199*
3,92E-05	4,64E-04	2,521	1,334	7,760	0,114	-0,061	0,061	-0,661	0,466
1,15E-08	2,99E-04	2,519	1,333	5,734	-1,430	-1,186	-0,629	-1,810	-0,916
7,08E-07	3,04E-02	2,509	1,327	7,801	2,501	1,177	1,754	2,367	0,882
9,35E-04	6,25E-03	2,485	1,313	10,520	0,081	-0,296	-0,117	-0,506	1,807
4,61E-07	2,22E-02	2,473	1,306	8,076	1,508	1,715	1,583	1,236	0,967
1,72E-04	1,07E-04	2,454	1,295	6,555	-0,048	-0,659	0,039	-0,670	-0,085
9,31E-07	2,44E-03	2,417	1,273	6,318	0,467	0,023	0,270	-0,023	0,746
2,42E-03	5,30E-04	2,403	1,265	5,490	0,141	0,120	-0,010	0,182	-0,045
2,08E-04	4,82E-05	2,383	1,253	9,589	0,186	-0,270	-0,074	-0,123	0,345
1,58E-09	7,36E-05	2,373	1,247	8,409	0,559	0,429	0,336	0,815	0,745
5,12E-08	5,16E-04	2,331	1,221	12,774	0,543	0,107	0,273	0,064	0,979
1,36E-02	4,28E-02	2,328	1,219	5,456	-0,026	-0,218	-0,117	0,005	2,500
2,29E-08	1,02E-02	2,326	1,218	11,385	1,038	1,446	2,797	2,387	1,082
4,70E-03	3,14E-02	2,326	1,218	4,264	0,159	-0,166	0,024	-0,440	0,184
1,14E-03	5,55E-03	2,315	1,211	5,194	0,001	0,608	0,175	0,308	0,272
1,45E-02	2,67E-02	2,291	1,196	8,070	-0,355	-1,256	-0,761	-1,387	0,467
5,29E-04	4,66E-03	2,278	1,188	7,700	0,033	-0,274	-0,030	-0,324	0,643
3,60E-08	4,81E-06	2,269	1,182	10,455	-0,567	-0,446	-0,601	-0,581	-0,026
8,33E-05	7,00E-05	2,269	1,182	5,696	-0,282	-0,409	-0,562	-0,442	-0,369
3,70E-09	2,32E-04	2,242	1,165	7,658	1,131	1,013	1,210	1,036	1,106
8,52E-08	1,32E-03	2,242	1,165	9,802	0,552	0,047	0,130	0,074	0,906
7,93E-06	7,93E-04	2,227	1,155	6,039	-0,244	0,100	0,362	0,131	-0,355
4,04E-06	2,80E-02	2,224	1,153	7,990	-1,735	-0,199	-2,632	-1,907	-1,868
1,22E-07	1,59E-04	2,198	1,136	8,469	0,210	0,232	0,315	0,061	-0,061
3,47E-02	1,53E-02	2,175	1,121	7,028	0,000	-0,910	0,000	-0,650	0,354
3,87E-03	2,38E-03	2,164	1,114	11,820	-0,021	0,231	0,021	-0,311	-0,506
1,41E-08	1,49E-02	2,152	1,106	10,912	1,049	1,630	2,858	2,575	1,149
9,14E-07	7,58E-03	2,133	1,093	7,557	0,115	0,218	0,277	0,074	0,377
2,74E-11	1,32E-03	2,120	1,084	6,581	-2,028	-2,599	-2,205	-1,691	-2,094
4,37E-06	4,10E-02	2,071	1,050	3,271	*-1,439*	*-1,443*	*-1,445*	*-1,453*	*-1,445*
5,99E-09	1,63E-03	2,066	1,047	5,853	0,747	1,188	0,965	0,429	1,207
1,72E-08	3,33E-02	2,058	1,041	11,196	1,385	0,486	0,795	0,489	1,942
1,24E-07	6,64E-07	2,042	1,030	11,463	-0,175	-0,123	-0,141	-0,411	-0,263
1,35E-03	4,38E-03	2,020	1,014	9,511	-0,978	-0,860	-0,947	-0,889	0,304
1,50E-10	1,98E-03	2,017	1,012	11,424	-1,285	-1,032	-1,039	-1,471	-1,897

CD4creGARPfl-fl_non-stim_22	CD4creGARPfl-fl_non-stim_23	CD4creGARPfl-fl_non-stim_8	CD4creGARPfl-fl_non-stim_9	CD4creGARPfl-fl_non-stim_10	wt_stim_11	wt_stim_12	wt_stim_13	wt_stim_14	wt_stim_15	CD4creGARPfl-fl_stim_24
2,227	3,640	1,003	0,585	0,955	-3,386	-3,346	-3,544	-2,710	-3,378	3,309
-1,188	-1,182	-0,254	5,723	-1,141	0,254	-1,142	2,056	2,066	0,901	6,174
3,510	3,620	3,664	3,739	3,684	-6,020	-5,715	-5,328	-5,289	-5,928	-1,498
4,036	4,285	4,449	4,594	4,503	-5,922	-5,647	-5,419	-5,287	-5,894	-1,650
4,774	5,291	5,818	6,266	5,838	-5,493	-5,571	-5,472	-5,730	-7,360	-1,568
2,156	3,934	3,344	5,025	4,313	-4,688	-4,925	-4,834	-4,275	-3,809	-4,393
0,158	2,525	3,928	0,305	2,461	-3,737	-1,986	-1,083	-3,540	-4,086	0,311
3,527	3,088	1,323	0,495	0,530	-2,007	-2,106	-2,146	-2,482	-2,025	2,528
0,451	2,086	0,219	0,250	0,154	-1,349	-1,738	-1,359	-2,808	-2,754	1,470
0,251	0,604	0,114	0,528	0,468	-2,891	-2,126	-1,544	-2,869	-2,873	0,595
1,921	3,168	2,260	4,132	2,296	-4,782	-4,328	-2,294	-4,780	-6,744	-1,648
1,423	2,473	2,150	2,705	2,074	-5,607	-6,114	-6,700	-6,184	-6,693	-2,022
0,972	2,468	1,700	2,948	1,262	-1,277	-2,583	-3,615	-3,870	-3,842	-0,078
2,300	2,066	1,851	3,514	2,547	-4,735	-4,792	-2,991	-3,195	-6,803	-0,294
4,601	2,682	3,380	3,266	3,175	-3,498	-2,870	-4,622	-4,601	-5,720	-0,853
2,946	3,531	3,270	3,545	2,998	-0,784	-2,062	-2,077	-2,068	-2,049	1,490
-0,811	-0,575	0,527	0,447	1,147	-0,807	0,313	-0,810	-0,815	-0,812	4,078
2,478	2,956	2,883	3,659	3,191	-3,382	-3,671	-3,925	-5,878	-5,729	-1,358
1,355	2,197	1,193	1,526	1,599	-2,344	-2,858	-3,307	-3,678	-3,308	-0,044
0,648	1,295	0,720	0,958	0,479	-2,306	-2,105	-3,492	-3,330	-3,493	-0,157
0,620	1,137	-0,248	0,810	0,337	-1,925	-0,847	-1,925	-1,909	-1,178	0,980
1,840	1,714	3,218	5,989	3,799	-4,028	-4,414	-3,879	-4,433	-4,416	-2,248
0,935	2,076	0,204	0,819	0,504	-0,948	-1,342	-1,509	-2,517	-0,342	2,023
2,807	1,459	1,680	1,412	5,488	-3,655	-5,614	-5,595	-5,627	-5,547	-2,229
2,459	2,430	2,582	3,416	2,928	-3,902	-4,376	-2,547	-2,601	-6,095	-0,113
-0,826	-0,283	1,385	0,615	0,329	-1,934	-0,798	-0,769	-2,301	-0,947	3,348
1,493	1,135	1,070	1,377	1,795	-1,877	-2,223	-1,662	-2,274	-2,545	1,102
1,386	1,867	2,230	2,014	2,061	-2,835	-3,392	-2,997	-2,943	-4,060	-0,146
0,695	1,227	0,456	0,624	1,598	-1,853	-2,082	-1,622	-1,796	-2,037	1,136
2,662	3,093	2,935	3,157	3,314	-3,771	-4,307	-3,518	-4,867	-5,181	-1,501
1,304	1,738	1,104	0,672	1,481	-4,161	-2,370	-1,949	-2,005	-4,044	1,657
-2,197	-2,175	-2,112	0,333	-2,083	0,883	1,451	0,822	1,201	-0,333	5,191
1,252	3,330	0,563	1,161	0,715	-2,061	-2,810	-2,397	-1,840	-1,135	0,795
-0,641	-0,126	-0,172	-0,542	-0,825	-1,309	-1,362	-1,436	-1,784	-0,101	1,303
1,765	2,305	2,250	2,533	2,363	-3,543	-4,576	-3,440	-3,153	-7,181	-1,155
1,561	2,246	2,417	2,680	2,150	-3,395	-4,038	-3,405	-3,787	-5,248	-1,424
-0,979	-0,754	-0,227	-0,554	-0,270	0,569	0,628	0,711	0,227	0,686	3,974
1,799	2,674	0,756	0,987	1,177	-1,850	-2,050	-1,683	-2,741	-1,033	1,022
-0,485	-0,318	-0,711	-0,410	0,881	-0,619	-0,038	-0,591	0,512	-0,774	1,733
2,544	4,165	4,680	4,288	2,705	-4,167	-3,386	-4,169	-2,157	-4,171	-1,337
1,353	2,083	2,199	2,614	6,085	-4,090	-4,302	-2,848	-3,578	-8,064	-0,946
-2,137	-2,098	-2,091	-1,674	-2,055	1,920	2,279	1,846	4,023	1,614	4,722
0,731	-0,234	0,183	-1,045	-0,183	-1,062	1,116	-1,060	1,114	-1,047	1,233
1,272	2,134	2,337	2,765	1,971	-3,432	-3,773	-3,599	-3,772	-3,986	-1,528
0,527	0,583	0,768	0,378	0,993	-1,649	-1,718	-1,311	-1,945	-0,372	0,965
2,120	2,016	3,123	5,907	3,930	-2,676	-5,421	-3,976	-3,476	-5,499	-1,769

1,557	2,254	2,498	2,687	2,153	-3,347	-3,854	-3,289	-3,668	-5,075	-1,489
0,205	-0,031	0,031	0,267	0,277	-0,111	0,285	-1,034	-1,519	-1,459	1,076
-0,392	0,126	0,249	-0,006	0,346	-1,486	-1,483	-0,796	-1,616	0,006	1,056
1,430	3,001	3,692	3,008	2,420	-1,788	-2,911	-1,813	-3,516	-4,527	-0,404
1,408	2,106	2,542	2,443	1,975	-3,676	-3,924	-2,813	-3,758	-5,208	-1,613
1,627	2,358	4,006	3,322	3,272	-2,993	-3,457	-2,961	-3,839	-4,742	-1,145
-0,844	-0,022	-0,806	-2,222	0,086	0,493	0,064	-0,163	-1,702	-0,808	2,890
2,617	2,824	3,312	3,883	3,440	-4,075	-3,549	-3,530	-4,100	-6,338	-1,403
1,382	2,147	2,239	2,566	1,817	-3,121	-3,704	-2,859	-3,895	-4,734	-1,577
1,336	2,093	1,852	2,150	1,914	-3,100	-3,461	-3,477	-2,592	-3,433	-1,076
0,777	1,313	1,014	1,418	2,101	-2,955	-3,418	-2,739	-3,683	-2,019	-0,465
3,048	2,895	2,697	3,625	3,859	-3,602	-3,734	-3,230	-3,612	-5,271	-1,467
1,649	1,890	3,317	1,968	3,094	-2,427	-3,653	-3,637	-3,653	-3,645	-1,296
0,094	0,132	0,114	-0,526	0,125	0,088	-1,847	-1,121	-1,913	-1,717	0,377
0,148	1,196	0,033	0,413	1,329	-1,508	-1,158	-1,295	-2,189	-1,334	1,401
2,831	2,429	2,383	3,078	3,715	-3,904	-3,732	-2,823	-4,273	-5,750	-1,225
0,859	2,068	1,691	2,152	1,615	-4,153	-4,214	-4,079	-4,561	-3,335	-1,380
2,375	3,464	3,472	4,076	3,420	-3,737	-3,716	-3,232	-3,893	-5,938	-1,395
0,909	2,036	1,126	0,889	1,498	-2,264	-2,431	-1,970	-2,018	-4,348	-0,201
1,090	2,121	0,619	0,754	0,536	-1,190	-2,331	-2,095	-2,404	-3,362	0,927
2,887	2,698	2,499	3,359	3,824	-3,730	-3,684	-3,092	-4,016	-4,970	-1,599
-0,030	0,194	-0,004	0,186	0,006	-0,024	-0,016	0,772	0,000	0,063	0,000
1,496	1,996	2,282	2,879	5,159	-3,885	-3,947	-2,949	-3,746	-5,877	-1,244
0,565	0,891	0,592	0,724	0,062	-1,417	-0,650	-0,062	-1,646	-2,218	1,571
2,363	2,374	2,441	2,512	2,047	-4,276	-3,434	-3,612	-4,795	-6,997	-0,887
0,917	1,378	0,729	-0,033	1,370	-1,214	-2,237	-1,543	-1,910	-1,891	1,800
-3,488	-3,858	-2,544	-4,074	-2,526	3,605	3,276	3,559	2,489	3,995	6,321
1,886	0,035	0,191	1,035	1,513	-0,765	-0,617	-1,976	-1,401	-1,934	0,721
2,685	2,966	2,863	3,084	3,499	-3,750	-4,023	-3,357	-4,203	-5,120	-1,572
1,543	2,242	2,491	2,675	2,141	-3,332	-3,657	-3,079	-3,875	-5,082	-1,623
1,786	2,306	2,308	2,592	2,360	-3,016	-3,841	-3,001	-2,830	-5,091	-0,842
0,133	-0,470	-1,720	0,006	0,746	-0,554	-1,099	-0,006	-1,851	-1,847	1,199
0,749	2,412	0,390	0,729	0,415	-1,666	-1,841	-1,201	-1,962	-1,301	0,356
4,475	3,432	2,668	2,751	2,991	-2,526	-2,834	-2,633	-2,363	-5,004	0,493
2,855	2,776	2,642	3,173	3,715	-3,842	-3,698	-3,243	-3,939	-5,261	-1,516
1,308	2,345	2,285	3,258	1,742	-3,093	-3,031	-3,944	-3,042	-6,571	-1,080
1,545	2,034	1,229	1,440	1,156	-2,880	-3,255	-2,604	-3,135	-4,074	-0,924
-0,542	0,328	-2,064	-2,397	-2,410	0,457	0,730	1,111	0,102	-0,171	5,317
2,673	3,296	3,703	4,046	3,229	-3,297	-3,423	-3,380	-3,701	-4,463	-1,286
-0,680	0,064	-0,859	-0,791	1,368	-0,839	-0,222	0,706	-0,064	-0,804	2,103
2,265	2,880	2,749	3,306	3,095	-3,509	-3,385	-3,057	-4,717	-7,228	-1,216
2,750	2,839	2,739	3,194	3,640	-3,851	-3,832	-3,363	-3,899	-4,867	-1,580
1,289	2,142	1,612	2,631	2,425	-3,426	-2,781	-2,973	-3,200	-4,694	-0,958
2,704	3,481	3,532	3,860	3,470	-3,898	-3,622	-3,411	-4,278	-5,212	-1,314
3,645	3,311	2,523	3,432	2,245	-2,852	-4,082	-3,357	-4,414	-3,461	-0,328
2,340	3,442	3,464	4,049	3,474	-3,758	-3,745	-3,393	-3,835	-5,304	-1,384
0,658	0,838	-0,065	-0,291	0,443	-0,004	0,190	0,004	-0,532	-0,627	2,948
-1,600	-1,225	-1,363	-1,777	-0,189	0,939	1,498	1,511	0,189	0,568	2,862
1,797	2,607	2,227	4,108	2,704	-3,021	-2,936	-3,240	-2,975	-4,751	-0,813
2,788	3,354	3,498	3,806	3,704	-3,818	-3,857	-3,610	-4,134	-5,766	-1,363
2,170	2,636	2,377	2,993	2,543	-4,011	-3,100	-3,828	-2,725	-6,077	-1,555
1,265	1,358	1,352	1,468	1,582	-3,223	-3,516	-3,205	-3,540	-4,170	-1,384
2,964	1,850	3,756	2,859	3,030	-3,395	-3,356	-3,499	-3,169	-6,528	-0,354
1,368	2,952	3,696	3,065	2,322	-1,864	-2,484	-1,755	-3,627	-6,802	-0,409
2,798	3,538	1,875	1,733	1,722	-2,743	-2,752	-1,712	-1,916	-1,820	1,490
0,324	1,525	0,710	0,448	-0,119	-0,325	-0,599	-0,242	-0,567	-1,145	1,574

0,087	0,240	-0,656	-0,940	-0,086	0,086	0,334	0,293	-0,270	-0,519	3,099
1,334	2,147	1,849	2,850	2,379	-3,082	-2,762	-2,882	-3,547	-4,026	-1,017
0,910	1,734	1,151	1,331	1,502	-2,884	-3,705	-2,388	-3,347	-2,964	-0,520
1,399	2,349	1,811	3,500	2,239	-2,825	-3,393	-4,869	-2,737	-6,485	-1,543
2,382	2,903	2,803	3,416	3,604	-3,185	-3,584	-3,159	-3,930	-3,513	-1,664
2,582	2,986	3,304	3,903	3,253	-3,112	-3,819	-3,368	-5,135	-4,127	-2,044
2,012	3,292	3,435	3,968	3,350	-2,939	-3,497	-3,325	-4,284	-5,994	-1,237
0,156	-0,032	-0,259	0,032	0,794	0,614	-0,224	-0,157	-0,620	0,808	2,596
0,011	0,674	0,297	0,509	1,431	-0,883	-1,777	-1,334	-2,100	-0,380	0,495
1,451	2,226	2,075	2,753	2,417	-3,014	-3,093	-3,035	-3,555	-4,316	-1,093
2,738	2,715	2,669	3,174	3,426	-3,571	-3,654	-3,211	-4,011	-4,838	-1,538
-0,418	-0,215	-0,292	0,026	0,647	0,479	-0,026	0,409	-0,201	0,325	2,882
1,795	2,218	2,734	2,893	2,125	-3,197	-3,532	-3,154	-3,739	-5,315	-1,721
1,519	3,461	2,429	3,336	2,804	-3,245	-3,202	-2,755	-4,546	-6,493	-1,435
2,814	1,748	2,586	2,000	2,621	-2,920	-2,461	-2,885	-2,900	-2,911	-1,091
-0,368	-0,053	0,366	-0,237	0,241	-0,060	0,391	0,053	0,566	-0,889	2,971
1,842	2,157	2,347	2,706	2,821	-3,823	-3,960	-3,585	-4,023	-4,522	-1,946
-0,054	0,419	0,936	1,403	2,066	-2,838	-3,532	-3,473	-3,775	-2,661	-0,681
-5,101	-3,176	-3,264	-2,940	-3,379	3,009	2,937	2,706	2,602	3,326	4,926
2,752	2,946	2,712	3,384	3,853	-3,543	-3,424	-3,112	-3,730	-4,439	-1,595
1,984	2,242	3,071	3,061	2,746	-3,183	-3,508	-2,913	-3,661	-5,462	-1,755
2,146	2,058	2,402	3,033	3,053	-3,520	-3,697	-2,982	-3,878	-4,456	-1,167
1,793	2,904	2,666	2,911	2,943	-3,565	-3,379	-2,999	-3,900	-5,738	-1,824
1,284	1,406	1,616	2,479	2,025	-3,044	-3,730	-3,229	-3,668	-3,765	-1,056
-1,714	-1,818	-1,903	-3,626	-1,787	2,600	2,187	2,761	1,714	2,207	4,932
0,433	0,683	0,871	1,431	2,292	-2,466	-3,543	-3,194	-3,156	-2,137	-0,152
-0,106	0,211	-0,465	-0,750	0,087	-0,007	0,091	0,007	-0,159	0,149	2,060
2,994	1,731	3,814	2,975	3,269	-3,006	-3,154	-3,070	-3,158	-6,984	-0,522
-0,282	-0,262	0,383	-0,101	-0,203	1,380	1,549	0,946	0,047	-0,047	3,530
0,600	1,864	0,285	0,709	0,702	-0,915	-1,744	-1,009	-1,687	-0,743	1,354
5,421	1,686	3,882	3,811	4,758	-1,608	-2,355	-1,137	-1,565	-1,901	1,034
-1,124	-0,457	-0,562	-0,633	0,631	0,897	0,393	0,736	-0,393	0,583	2,475
2,619	3,058	3,345	3,777	3,843	-3,923	-3,943	-3,733	-4,048	-5,808	-1,198
1,142	2,889	0,494	0,997	0,572	-1,077	-1,953	-1,375	-1,693	-1,804	0,713
3,156	2,749	2,602	3,741	2,998	-3,758	-3,443	-2,958	-4,066	-6,615	-1,384
1,173	1,336	1,472	2,239	1,913	-3,032	-3,662	-3,199	-3,785	-3,715	-1,035
1,272	2,112	1,741	2,846	2,446	-2,841	-2,483	-2,775	-3,436	-4,019	-0,973
0,983	1,250	1,087	0,993	1,476	-1,452	-1,707	-1,407	-2,022	-1,495	0,146
-0,822	-0,219	-0,725	-0,784	-0,357	0,937	0,535	0,623	0,219	0,898	2,346
-1,576	-1,528	-1,832	-1,226	-0,772	1,403	0,878	1,105	0,772	1,627	3,076
-5,025	-3,773	-5,787	-4,588	-1,903	2,021	2,121	2,224	1,903	2,107	6,071
0,908	1,908	-0,218	-0,776	0,001	-0,063	-0,001	-0,373	0,092	-0,141	5,190
0,919	1,597	1,683	1,737	1,385	-2,634	-3,161	-2,499	-3,359	-3,059	-0,919
-0,117	0,731	0,310	0,074	0,401	-1,215	-1,147	-0,710	-1,085	0,106	0,715
1,219	1,769	1,628	1,885	1,459	-1,621	-2,355	-1,951	-2,350	-3,013	-0,544
0,889	1,397	1,572	2,096	1,779	-3,130	-3,664	-3,646	-3,571	-3,656	-1,040
2,605	2,584	2,759	3,422	2,923	-4,075	-3,135	-3,353	-3,420	-5,724	-1,372
-0,400	-0,044	-0,710	-1,055	0,099	0,169	0,331	0,044	-0,480	0,311	1,916
0,988	1,376	1,353	0,977	1,047	-2,828	-1,185	-2,867	-2,050	-0,316	-0,279
-0,179	-0,303	-0,003	0,209	-0,246	0,030	0,338	-0,120	-0,225	-0,133	3,864
1,468	1,854	1,955	2,974	1,820	-3,023	-2,900	-2,972	-3,311	-4,388	-1,565
0,527	2,196	-0,018	0,484	0,429	-1,040	-2,291	-0,864	-2,182	-0,623	1,584
-0,585	-1,081	-1,872	-2,409	-2,482	0,922	1,024	0,819	0,585	0,623	3,190
2,383	2,113	1,798	1,949	1,845	-4,238	-4,214	-4,485	-4,789	-4,373	-1,108
0,547	1,034	0,769	1,023	1,412	-1,066	-1,526	-1,379	-1,869	-0,818	0,446
0,453	1,150	0,439	0,692	1,012	-1,399	-1,425	-1,133	-1,563	-0,964	0,038

2,919	1,778	1,936	2,082	5,102	-4,426	-4,426	-4,350	-4,017	-7,254	-1,864
1,855	2,573	3,293	3,233	1,943	-4,708	-2,987	-3,381	-2,015	-2,439	-0,779
0,058	0,436	0,239	0,173	0,522	-0,514	-0,766	-0,546	-1,122	-1,033	1,073
1,171	1,372	0,541	0,764	0,587	-1,428	-1,395	-1,102	-3,287	-1,609	1,760
-1,580	-2,478	-0,967	-1,549	-1,863	1,673	1,867	1,953	0,967	1,360	4,190
0,953	1,457	0,661	1,053	0,497	-1,364	-1,551	-1,287	-2,031	-1,716	1,281
-1,329	-0,579	-1,202	-0,805	-0,348	0,797	0,768	0,452	0,348	0,424	2,076
2,294	2,094	3,703	5,503	4,133	-2,424	-5,374	-2,744	-2,779	-5,659	-1,806
1,265	2,101	0,944	1,232	1,277	-0,814	-1,189	-0,919	-1,318	-0,849	1,613
1,313	2,272	1,353	2,113	2,417	-3,710	-2,944	-3,182	-2,435	-6,155	-0,967
0,157	0,636	0,450	0,721	0,496	-0,350	-0,859	-0,694	-1,491	-0,653	1,177
0,463	1,049	1,244	1,339	1,953	-2,589	-2,024	-1,576	-1,998	-1,373	-0,543
-0,243	0,085	-0,069	0,018	-0,315	-0,106	-0,431	-1,017	-0,927	-1,734	0,976
0,158	0,741	-0,015	0,015	0,260	-0,530	-0,650	-0,521	-0,707	-1,064	0,746
0,486	1,056	0,848	0,766	0,433	-2,339	-2,176	-2,061	-2,368	-2,443	-0,336
0,772	1,413	0,991	1,248	1,659	-2,157	-2,497	-2,310	-2,832	-1,416	-0,745
0,754	2,091	0,450	0,796	0,833	-0,845	-1,000	-0,723	-1,334	-0,839	0,912
1,680	1,754	1,659	1,723	1,501	-3,171	-3,541	-2,956	-2,317	-5,049	-1,953
0,569	0,875	0,318	0,486	0,813	-2,005	-1,242	-0,137	-1,332	-0,550	0,552
-0,001	0,575	0,349	1,070	0,389	-1,241	-0,681	-1,107	-1,281	-3,401	0,639
0,014	0,821	0,287	0,351	1,446	-0,604	-1,136	-0,922	-1,764	0,034	0,274
0,342	0,720	0,644	0,918	1,444	-0,896	-1,464	-1,158	-1,253	-0,224	-0,062
-0,847	-0,197	-0,669	-0,655	-0,346	0,201	0,189	0,026	0,067	0,388	1,636
0,873	1,132	0,644	0,563	0,264	-0,370	-0,481	-0,264	-0,556	-1,746	1,275
0,588	0,988	0,539	0,545	0,021	-1,654	-2,069	-1,551	-1,969	-2,005	-0,804
0,572	1,124	1,196	1,494	1,953	-2,315	-3,083	-2,604	-3,093	-1,984	-1,010
0,390	0,750	1,236	0,801	1,084	-1,239	-1,896	-0,872	-1,256	-0,619	-0,084
-0,551	-1,846	-2,218	-2,538	-0,757	0,587	0,825	0,543	0,199	0,230	2,760
0,789	2,035	1,085	0,883	0,594	-1,815	-1,609	-1,702	-1,684	-1,973	-0,146
-0,272	0,517	-0,074	0,587	1,206	-0,629	-1,525	-0,496	-1,587	0,363	0,723
-0,679	0,193	0,327	0,652	0,367	-0,861	-1,095	-1,200	-0,594	-1,004	0,943
1,712	1,974	1,724	1,854	1,656	-2,807	-3,330	-2,815	-1,997	-4,469	-1,710
1,272	0,939	0,589	0,522	0,144	-1,954	-2,057	-1,679	-2,467	-2,727	-0,074
-1,956	-2,031	-2,193	-2,511	-2,101	1,804	2,023	1,691	1,978	1,691	4,390
0,068	-0,999	-1,430	-0,178	-1,015	0,178	0,793	1,079	-0,068	0,129	2,009
1,521	1,683	1,239	0,980	1,115	-2,230	-2,714	-1,703	-3,261	-2,442	-1,111
1,598	2,169	1,495	1,876	2,413	-2,151	-2,648	-2,519	-3,026	-1,660	-1,197
0,378	0,896	0,711	0,685	0,908	-0,748	-0,633	-0,646	-0,718	-0,774	0,806
0,911	0,304	-0,020	0,024	0,705	-0,559	-0,429	-0,381	-1,053	-0,616	0,994
-2,637	-1,553	-1,387	-1,245	-1,697	1,363	1,199	1,297	1,032	1,061	2,211

CD4creGARPfl-fl_stim_25	CD4creGARPfl-fl_stim_18	CD4creGARPfl-fl_stim_19	CD4creGARPfl-fl_stim_20	Refseq	GeneID
3,748	0,298	-1,385	0,988	NM_009139	20305
4,380	4,737	3,797	5,578	NM_008198	14962
-1,914	-1,612	-2,845	-2,432	XM_003085465	
-1,980	-1,617	-2,899	-2,458	XM_001472091	
-1,831	-2,058	-2,949	-2,655		16061
-1,253	-3,103	-0,890	-0,955		
-0,259	-0,158	-1,074	0,687		
2,955	-0,067	0,067	1,134	NM_010370	14938
1,592	1,441	-0,133	0,133	NM_024470	79410
0,281	-1,217	0,503	0,083	NM_001109657	14457
-0,255	-3,085	-1,672	-1,122		
-1,423	-4,072	-4,203	-3,066	NM_001159904	17059
-2,208	-0,565	-0,188	-1,644		
-1,710	-2,761	-2,350	-1,259		
-1,889	-2,309	-1,432	-1,618		
1,358	0,196	0,769	0,681	NM_007640	12480
3,172	0,291	-0,575	1,965	NM_019948	56619
-1,215	-2,801	-0,808	-1,024	XM_904758	
-0,048	-1,985	-1,086	-0,612	NM_153590	243655
-0,413	-1,959	-0,948	-0,646	NM_033078	27007
0,765	0,248	0,457	0,903	NM_008463	16636
-1,809	-2,652	-1,242	-0,645		
1,840	-0,204	0,778	1,246	NM_024470	79410
-3,020	-3,840	-3,413	0,400		
-1,370	-1,600	-1,751	-0,898		
2,114	0,365	0,047	1,569	NM_008361	16176
0,279	-0,636	-0,466	1,180	NM_001077189	14130
-1,288	-2,150	-0,505	-0,221		
0,949	-0,817	-0,118	0,631	NM_011094	18730
-1,994	-2,609	-1,827	-1,163		
0,021	1,107	-2,757	0,086	NM_009369	21810
3,818	2,893	2,957	3,309	NM_010558	16191
1,625	-0,926	-0,316	0,353	NM_010649	16635
1,259	0,101	0,379	1,048	NM_010371	14940
-1,228	-2,146	-1,059	-0,541		
-1,688	-2,233	-1,365	-0,864		
2,970	2,395	1,795	3,209	NM_008392	16365
0,710	-0,452	-0,511	0,481	NM_021334	16411
0,655	2,021	0,151	1,980	NM_011888	24047
-1,927	-1,862	-2,527	-0,959		
-1,949	-1,795	-2,452	1,336		
4,008	3,490	4,213	4,551	NM_008373	16198
1,684	1,404	1,174	0,857	NM_008179	14853
-1,874	-2,323	-1,109	-0,707		
0,838	-0,068	0,068	0,592	NM_146191	233328
-2,171	-2,934	-0,889	-0,789		

-1,796	-2,300	-1,332	-0,846		
0,899	1,185	1,145	1,762	NM_023143	50909
0,476	0,695	0,663	1,065	NM_010736	17000
-1,666	-0,077	-0,743	-1,159		
-1,913	-2,615	-1,480	-0,851		
-1,943	-1,712	-1,312	-1,210		
2,028	0,256	1,410	1,967	NM_007670	12579
-2,292	-3,119	-1,919	-1,946	XM_003086781	100505009
-2,178	-2,384	-1,378	-1,030		
-1,342	-2,157	-1,308	-0,526		
-0,835	-2,066	-0,960	0,147	NM_145141	98752
-2,356	-2,439	-1,499	-1,475		
-1,579	-3,654	-1,128	-1,533		
0,350	0,587	-0,353	0,624	NM_021297	21898
0,756	0,096	-0,033	1,007	NM_011095	18733
-2,368	-2,368	-1,820	-1,241		
-0,859	-2,725	-3,342	-2,080	NM_010737	17057
-2,121	-2,865	-1,665	-1,516		
0,210	-1,532	-0,575	-0,017	NM_007549	12143
0,436	-1,102	-1,201	-0,271	NM_177155	320407
-2,699	-2,674	-1,674	-1,346		
3,007	2,053	2,592	-0,009	NR_045120	100417675
-2,513	-1,834	-2,529	0,481		
0,634	0,125	0,491	1,314		15951
-2,540	-4,087	-2,150	-2,227		
0,156	-1,054	-0,625	0,533	NM_010188	14131
5,624	5,197	4,926	5,605	NM_019577	56221
0,638	0,644	0,198	0,622	NM_007643	12491
-2,330	-2,549	-1,987	-1,148		
-1,821	-2,232	-1,332	-0,921		
-1,139	-2,138	-0,983	-0,638		
0,927	0,975	0,905	0,896	NM_001025602	17082
1,217	-0,728	-0,392	0,359	NM_010650	16639
-0,622	-2,968	-1,271	-0,594		
-2,480	-2,477	-1,832	-1,362		
-1,454	-1,938	-0,644	-1,087		
-0,792	-1,136	-1,938	-1,246	NM_009915	12772
5,060	1,762	1,731	2,447	NM_031168	16193
-1,621	-2,185	-1,167	-1,456		
1,778	1,380	0,863	1,745	NM_026100	67344
-1,619	-2,389	-1,547	-1,262		
-2,468	-2,455	-1,891	-1,315		
-1,994	-2,454	-1,243	-0,986		
-2,296	-3,040	-1,944	-1,653		
-1,277	-2,753	-2,004	-1,512		
-2,142	-2,727	-1,835	-1,588	XM_918237	640979
2,091	1,160	1,167	1,918	NM_013532	14728
2,532	1,915	2,856	2,888	NM_001039701	16181
-1,298	-1,650	-1,056	-1,104		
-2,326	-3,072	-1,949	-1,596		
-1,565	-2,544	-1,921	-1,921		
-2,086	-2,246	-1,627	-0,700		
-2,295	-2,828	-1,515	-1,254		
-1,649	-0,130	-0,609	-1,031		
0,830	-2,074	-0,048	-0,150	NM_010746	17086
1,876	1,046	1,017	1,302	NM_144548	209590

2,177	0,986	1,404	1,954	NM_013532	14728
-1,751	-2,307	-1,228	-0,949		
-1,112	-1,364	-1,644	-0,263	NM_028728	74039
-3,331	-2,902	-1,544	-0,742		
-2,216	-2,311	-1,527	-1,326		
-1,977	-3,782	-0,951	-1,221		
-2,117	-3,417	-1,662	-1,510		16061
1,582	1,488	1,675	2,401	NM_009877	12578
0,165	-0,011	0,613	1,809	NM_009844	12478
-1,544	-2,382	-1,265	-0,911		
-2,311	-2,563	-1,834	-1,363		
2,139	0,930	1,384	2,179	NM_007670	12579
-2,309	-2,296	-1,474	-1,009		
-1,987	-3,384	-1,286	-0,645		
-2,080	*-1,626*	-0,536	-0,344		
2,620	1,858	1,556	1,823	NM_016971	50929
-2,497	-2,339	-2,164	-1,156	NM_152839	16069
-2,323	-2,012	-1,682	0,245		
4,728	4,000	4,589	4,713	NM_008355	16163
-2,590	-2,250	-1,786	-1,427		
-2,206	-2,363	-1,346	-0,830		
-2,488	-2,553	-1,946	-0,966		
-2,431	-2,500	-1,650	-1,024		
-2,512	-2,331	-1,935	-0,814		
3,768	3,935	3,683	4,673	NM_008599	17329
-1,671	-1,590	-1,451	0,589		
1,762	1,014	1,231	1,710	NM_011617	21948
-2,431	-2,571	-1,457	-1,312		
2,643	2,925	2,289	2,202	NM_203320	330122
1,225	-0,753	0,361	0,681	NM_001110323	16638
0,079	-0,252	-0,931	0,570	NM_009396	21928
2,264	1,893	1,980	2,690	NM_011611	21939
-2,637	-3,200	-2,271	-1,435		
1,093	-0,691	-0,280	-0,029	NM_010650	16639
-2,584	-3,366	-2,101	-1,854		
-2,390	-2,293	-2,008	-0,785		
-1,714	-2,491	-1,198	-0,976		
0,129	-0,614	-0,367	0,152	NM_022021	63955
2,371	1,655	1,691	2,242	NM_010215	14204
3,069	2,552	2,639	2,721	NM_011332	20295
4,489	3,224	3,205	3,724	NM_010556	16187
2,667	0,804	1,535	1,464	NM_011338	20308
-1,375	-1,607	-1,976	-1,475	NM_015790	50723
0,492	0,323	-0,074	0,760	NM_020044	56743
-0,522	-1,530	-0,781	-1,222	NM_007640	12480
-2,496	-2,683	-2,105	-0,467		
-2,095	-3,140	-1,894	-1,869		
1,673	1,275	1,196	1,951	NM_010555	16178
-0,845	-0,566	-0,558	*-0,866*	NM_001134458	16199
1,489	0,982	1,268	1,344	NM_008337	15978
-2,254	-1,933	-1,256	-0,716		
1,468	-0,340	0,251	0,414	NM_014194	16638
2,912	1,611	1,954	2,270	NM_013542	14939
-2,813	-3,366	-3,284	-2,926	NM_013653	20304
0,064	-0,149	-0,064	1,060	NM_001042605	16149
0,293	-0,230	-0,489	0,056	NM_010580	16419

-3,210	-3,753	-3,006	0,243	XM_001476673	100046710
-2,078	-2,746	-1,239	-1,612		
0,610	0,330	0,550	1,265	NM_021274	15945
1,108	-0,677	-0,357	-0,059	NM_030599	80782
3,038	2,863	3,007	3,804	NM_008599	17329
0,583	-0,638	-0,513	-0,217	NM_030599	80782
1,845	1,409	1,633	1,784	NM_009401	21941
-1,452	-2,407	-1,035	-0,882		
1,193	-0,143	-0,081	0,394	NM_010185	14127
-2,021	-3,161	-1,875	-1,018		
0,601	-0,039	0,412	0,797	NM_009142	20312
-1,121	-0,725	-0,885	0,325	NM_007655	12518
0,534	0,081	0,337	0,010	NM_023158	66102
0,604	-0,044	0,238	0,632	NM_012057	27056
-0,772	-1,497	-1,564	-1,093	NM_008329	15951
-1,089	-1,162	-1,103	-0,064	NM_008339	15985
1,104	-0,291	-0,005	0,374	NM_010651	16640
-2,214	-1,574	-2,207	-1,038		
-0,024	-0,034	-1,143	0,677	NM_008795	18557
-0,001	-0,519	-0,029	-0,099	NM_010652	16641
-0,088	-0,014	0,585	1,772	NM_001169153	246746
-0,282	0,030	0,111	1,188	NM_028075	72049
1,766	1,100	1,063	1,370	NM_019465	54698
1,776	0,575	0,701	0,692	NM_008362	16177
-0,936	-1,270	-0,755	-0,021	NM_133211	170743
-1,520	-1,550	-1,440	-0,047		12518
0,084	-0,348	-0,321	0,329	NM_024124	79221
1,785	1,564	1,697	1,488	NM_021782	60505
0,141	-0,567	-0,823	-0,758	NM_001034031	57890
0,361	-0,020	0,492	1,849	NM_008370	16192
0,259	-0,503	0,110	0,070	NM_008510	16963
-2,042	-1,529	-1,964	-1,049		
-0,865	-1,970	-1,408	-0,964	NM_010553	16174
3,225	2,416	2,888	2,717		20290
2,003	0,598	1,013	1,228	NM_001079876	237436
-1,395	-1,424	-1,702	-0,429	NM_010871	17952
-1,479	-1,873	-1,803	-0,486	NM_026976	69169
0,312	0,123	0,168	0,635	NM_007609	12363
0,865	0,020	0,130	0,455	NM_001025602	17082
2,341	1,843	2,286	2,210	NM_021283	16189
